# Supplementary material for: PGF2α facilitates pathological retinal angiogenesis by modulating endothelial FOS‐driven ELR + CXC chemokine expression
Source: EMBO Mol Med. 2022 Dec 13;15(1):e16373. doi: 10.15252/emmm.202216373 (PMC9832840; doi:10.15252/emmm.202216373)
Supplement: Supplementary file 1 — Appendix [file EMMM-15-e16373-s009.pdf]

Table of content

Appendix Figure S1. Normal retinal vascular development in endothelial cell-specific PTGFR-deficient mice.

Appendix Figure S2. Expression of *VEGFA* and its receptors in $PGF_{2\alpha}$-treated HRMECs and *Vegfa* and its receptors in retinas from endothelial PTGFR-deficient mice of OIR model.

Appendix Figure S3. Expression of *CXCR2* and *CXCR1* in $PGF_{2\alpha}$-treated HRMECs.

Appendix Figure S4. $PGF_{2\alpha}$ treatment elevates *FOS* expression in HRMECs.

Appendix Figure S5. *FOS* knockdown eliminates the effect of $PGF_{2\alpha}$ on *CXCL2* mRNA expression and secretion by cultured HRMECs.

Appendix Figure S6. Effect of *CAMK2G* or *CAMK2D* knockdown on $PGF_{2\alpha}$-induced *CXCL8* expression in HRMECs.

Appendix Figure S7. Normal retinal vascular development in *Cxcr2*$^{-/-}$ mice.

Appendix Figure S8. Effect of PGTFR inhibitor on body weight in OIR mice (P12-P17)

Appendix Table S1. DESeq2 results for target genes in the GSE94019 dataset

Appendix Table S2. List of primers

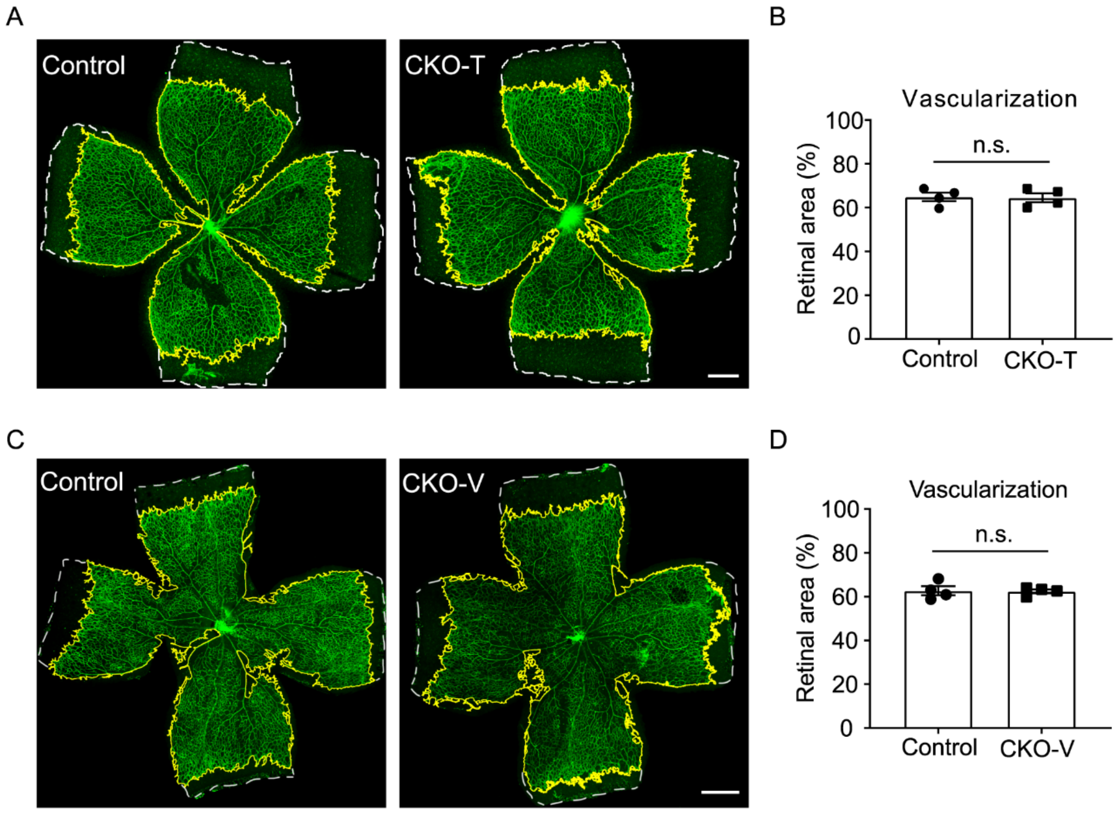

Appendix Figure S1. Normal retinal vascular development in endothelial cell-specific PTGFR-deficient mice.

A．Representative images of retinal vascular staining in CKO-T and control mice on postnatal day 7. The green shows isolectin B_4-stained blood vessels; yellow circles show the area of blood vessels in the retina. B. Quantitation of retinal blood vessel area in A (n=4). C. Representative images of retinal vascular staining in CKO-V and wild-type mice on postnatal day 7. The green shows isolectin B_4-stained blood vessels; yellow circles show the area of blood vessels in the retina. D. Quantitation of retinal blood vessel area in C (n=4). n.s. stands for “not significant.” Data were analyzed by the Mann–Whitney test (B, D). Scale bar: 500 μm (A, C).

PTGFR: $PGF_{2\alpha}$ receptor

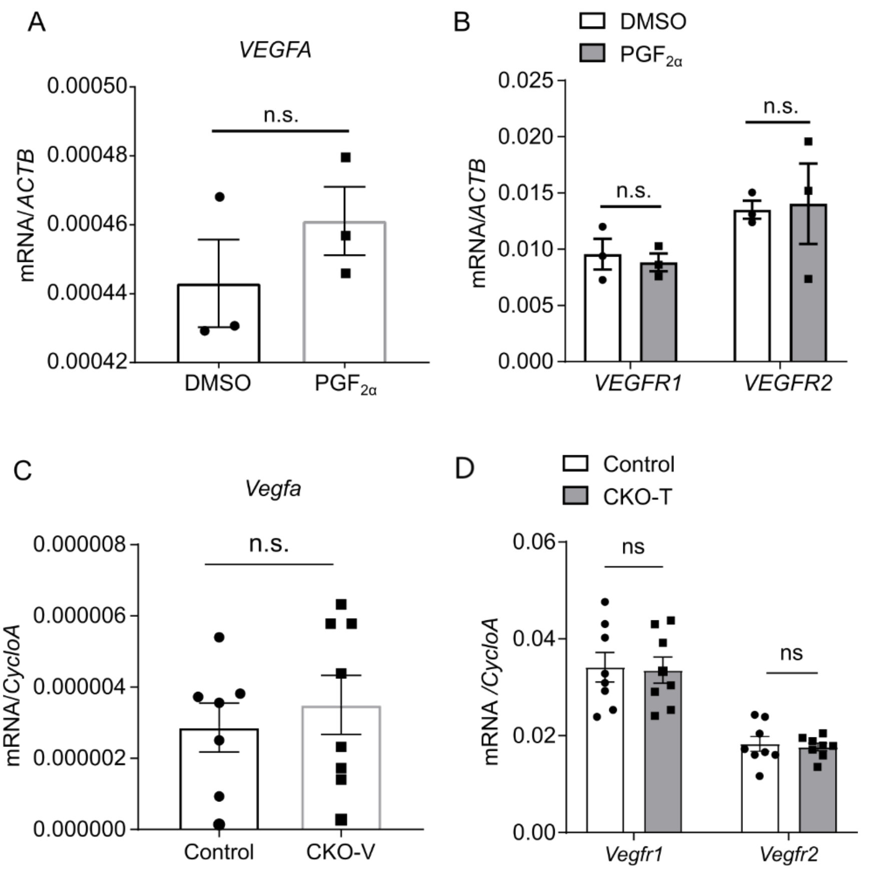

Appendix Figure S2. Expression of *VEGFA* and its receptors in $PGF_{2\alpha}$-treated HRMECs and *Vegfa* and its receptors in retinas from endothelial PTGFR-deficient mice of OIR model.

A. Effect of $PGF_{2\alpha}$ treatment on *VEGFA* expression in HRMECs (n=3). B. Effect of $PGF_{2\alpha}$ treatment on *VEGFR1* and *VEGFR2* expression in HRMECs (n=3). Expression of *Vegfa* and its receptors in retinas from endothelial PTGFR-deficient mice(n=7-8). n.s. stands for "not significant." Data were analyzed by the unpaired student's *t*-test (A, B, C, D).

VEGF: vascular endothelial growth factor; PTGFR: $PGF_{2\alpha}$ receptor; OIR: oxygen-induced retinopathy; HRMEC: human retinal microvascular endothelial cell

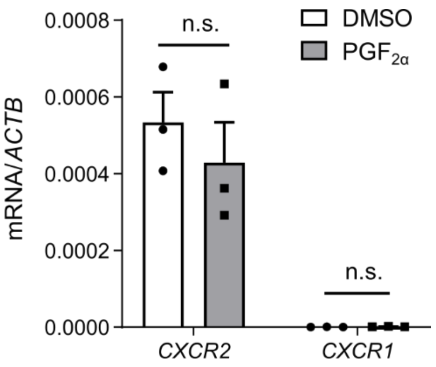

Appendix Figure S3. Expression of *CXCR2* and *CXCR1* in $PGF_{2\alpha}$-treated HRMECs.

Cultured HRMECs were treated with 500 nM $PGF_{2\alpha}$. *CXCR2* and *CXCR1* expression was examined by RT-PCR. Data were analyzed by the unpaired student's *t*-test (n=3). n.s. stands for "not significant."

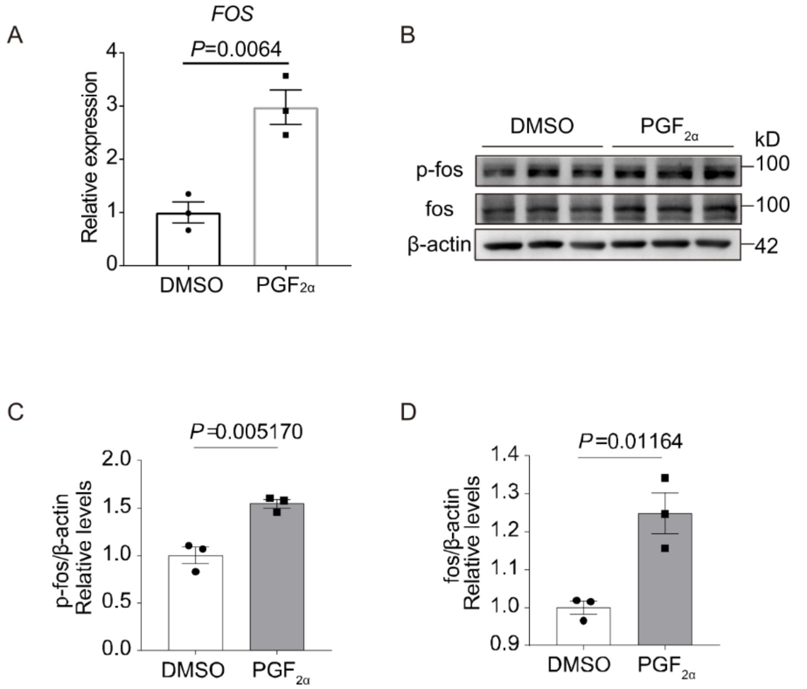

Appendix Figure S4. $PGF_{2\alpha}$ treatment elevates *FOS* expression in HRMECs.

A. Cultured HRMECs were treated with 500 nM $PGF_{2\alpha}$, *FOS* expression was examined by RT-PCR (n=3). B. Representative western blots for treatment with 500 nM $PGF_{2\alpha}$ in HRMECs. C. and D. Quantification of blots shown in B (n=3), Data were analyzed by the unpaired student's *t*-test.

HRMEC: human retinal microvascular endothelial cell

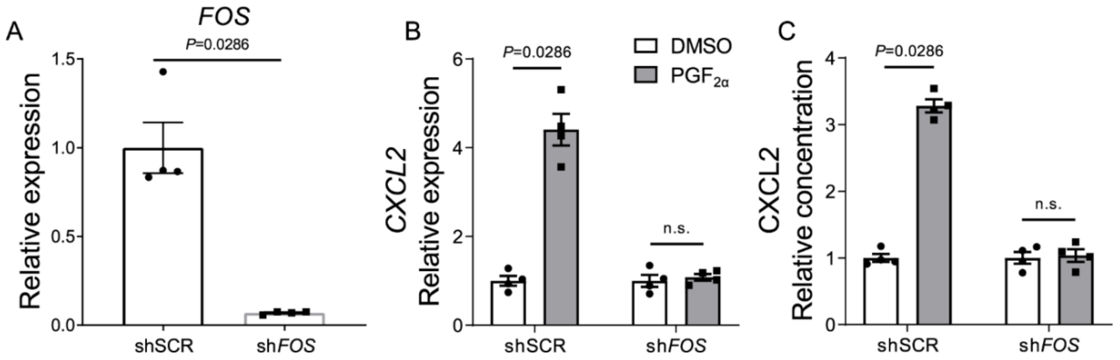

Appendix Figure S5. *FOS* knockdown eliminates the effect of $PGF_{2\alpha}$ on *CXCL2* mRNA expression and secretion by cultured HRMECs.

A. Efficiency of the lentivirus-mediated *FOS* knockdown in HRMECs (n=4). B-C. *CXCL2* mRNA expression and secretion by cultured HRMECs (n=4). n.s. stands for "not significant." Data were analyzed by the Mann–Whitney test (A, B, C).

HRMEC: human retinal microvascular endothelial cell

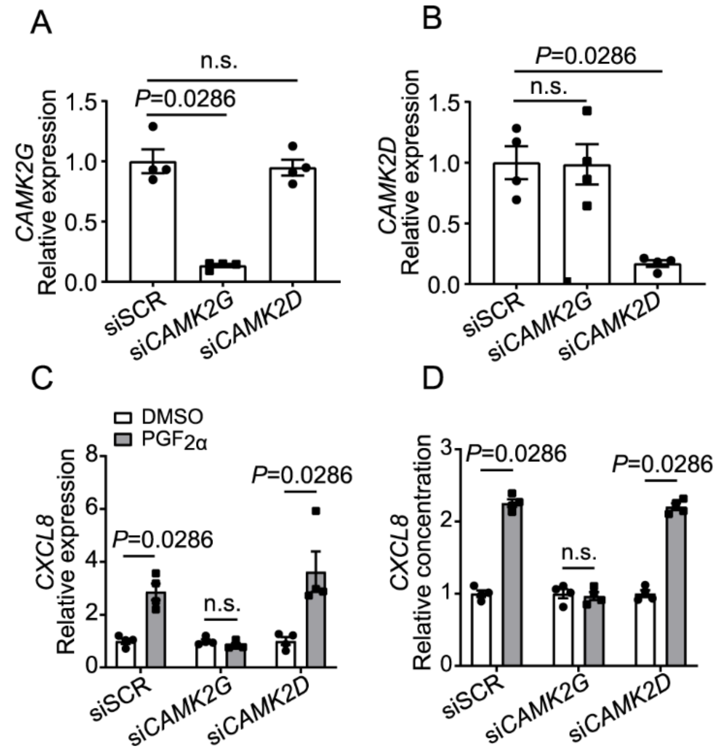

Appendix Figure S6. Effect of *CAMK2G* or *CAMK2D* knockdown on $PGF_{2\alpha}$-induced *CXCL8* expression in HRMECs.

A. *CAMK2G* knockdown efficiency in HRMECs using siRNA (n=4). B. *CAMK2D* knockdown efficiency in HRMECs using siRNA (n=4). C. Effect of *CAMK2G* or *CAMK2D* knockdown on $PGF_{2\alpha}$-induced *CXCL8* mRNA expression in HRMECs (n=4). D. Effect of *CAMK2G* or *CAMK2D* knockdown on $PGF_{2\alpha}$-induced CXCL8 secretion in the culture medium (n=4). n.s. stands for "not significant." Data were analyzed by the Mann–Whitney test (A, B, C, D).

HRMEC: human retinal microvascular endothelial cell

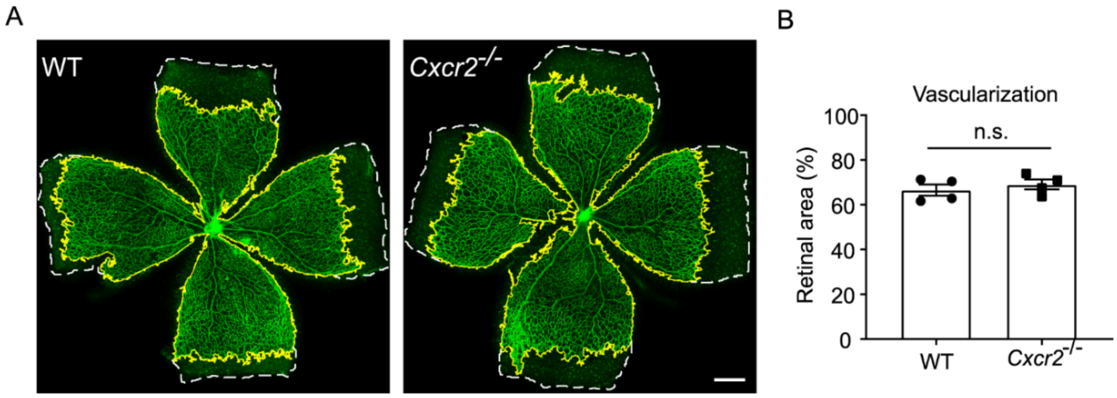

Appendix Figure S7. Normal retinal vascular development in *Cxcr2*$^{-/-}$ mice.

A. Representative images of retinal vascular staining in *Cxcr2*$^{-/-}$ and control mice on postnatal day 7. The green shows isolectin B_4-stained blood vessels; yellow circles show the area of blood vessels in the retina. Scale bar: 500 μm. B. Quantitation of retinal blood vessel area in A (n=4). n.s. stands for “not significant.” Data were analyzed by the Mann–Whitney test (B).

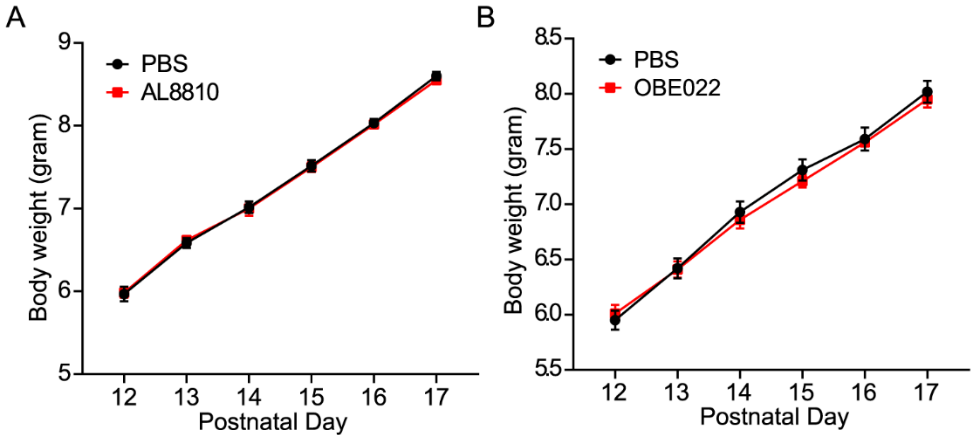

Appendix Figure S8. Effect of PGTFR inhibitor on body weight in OIR mice (P12-P17)

A. Effect of AL8810 on body weight in OIR mice at P12-P17 (n=6); B. Effect of OBE022 on body weight in OIR mice at P12-P17 (n=10). B. PTGFR: $PGF_{2\alpha}$ receptor; OIR: oxygen-induced retinopathy

Appendix Table S1. DESeq2 results for target genes in the GSE94019 dataset

	**log2FoldChange**	**pvalue**	**padj**
CXCL8	5.013933318	1.29E-07	3.54E-05
CXCL2	5.434172376	1.43E-09	1.23E-06
CXCL3	6.04191412	9.04E-08	2.83E-05
CXCL5	2.504343526	0.067207862	0.179902139
FOS	2.305802297	3.15E-05	0.00157818

Appendix Table S2. List of primers

Gene	Forward (5'→3')	Reverse (5'→3')
**Human**		
ACTB	AGAGCTACGAGCTGCCTGAC	AGCACTGTGTTGGCGTACAG
CAMK2D	GGATCTGTCAACGTTCTACT	TGTGGATTACAGTAGTTTGG
CAMK2G	TCCTGTATATCCTCCTGGT	CATCTGGTTGATCAAGTTC
CXCL2	TGCCAGTGCTTGCAGAC	TCTTAACCATGGGCGATGC
CXCL3	ATCCAAAGTGTGAATGTAAGGTC	GCAGGAAGTGTCAATGATACG
CXCL5	CCGCTGCTGTGTTGAGAG	TCTGCTGAAGACTGGGAAAC
CXCL8	CTGGCCGTGGCTCTCTTG	CTTGGCAAAACTGCACCTT
CXCR1	CTGACCCAGAAGCGTCACTTG	CCAGGACCTCATAGCAAACTG
CXCR2	ATTCTGGGCATCCTTCACAG	TGCACTTAGGCAGGAGGTCT
FOS	CATGGGCTCGCCTGTCAACG	AGGGGCTCTGGTCTGCGATG
VEGFA	AGGGCAGAATCATCACGAAGT	AGGGTCTCGATTGGATGGCA
VEGFR1	GAAAACGCATAATCTGGGACAGT	GCGTGGTGTGCTTATTTGGA
VEGFR2	GGCCCAATAATCAGAGTGGCA	CCAGTGTCATTTCCGATCACTTT
**Mouse**		
Akr1a1	AGCCTGGTCAGGTGAAAGC	GGCCTCCCCAATCTCAGTT
Akr1b3	AGGCCGTGAAAGTTGCTATTG	ATGCTCTTGTCATGGAACGTG
Akr1b7	TTGACTGTGCCTATGTGTATCAC	GGTGTTGTCGAAGGCTTTCTT
Cdh5	ATTGGCCTGTGTTTTCGCAC	CACAGTGGGGTCATCTGCAT
Cxcl1	ACCCAAACCGAAGTCATAGCC	TTGTCAGAAGCCAGCGTTCA
Cxcl2	CAGAATTCACTTCAGCCTAGCGCCAT	GCTCTAGAGTCAGTTAGCCTTGCCTTTG
Cxcl3	CAGCCACACTCCAGCCTA	CACAACAGCCCCTGTAGC
Cxcl5	TCCAGCTCGCCATTCATGC	TTGCGGCTATGACTGAGGAAG
CypA	CAGACGCCACTGTCGCTTT	TGTCTTTGGAACTTTGTCTGCAA
Fos	CGGGTTTCAACGCCGACTA	TTGGCACTAGAGACGGACAGA
Ptgfr	CTGGCTTGTGCCCACT	GACGGCATTGCACGAGA
Vegfa	CTGCCGTCCGATTGAGACC	CCCCTCCTTGTACCACTGTC
Vegfr1	CCACCTCTCTATCCGCTGG	ACCAATGTGCTAACCGTCTTATT
Vegfr2	TTTGGCAAATACAACCCTTCAGA	GCAGAAGATACTGTCACCACC
