# Supplementary material for: PGF2α facilitates pathological retinal angiogenesis by modulating endothelial FOS‐driven ELR + CXC chemokine expression
Source: EMBO Mol Med. 2022 Dec 13;15(1):e16373. doi: 10.15252/emmm.202216373 (PMC9832840; doi:10.15252/emmm.202216373)
Supplement: Supplementary file 2 — Expanded View Figures PDF [file EMMM-15-e16373-s006.pdf]

Expanded View Figures

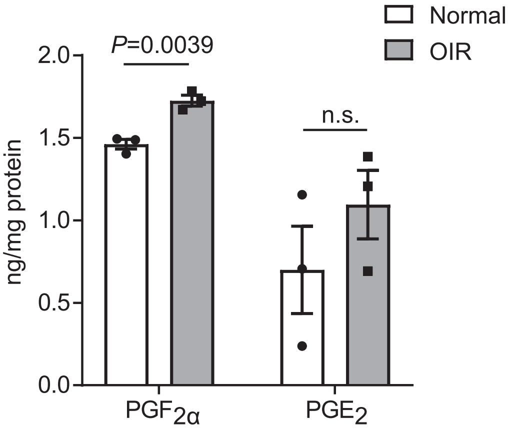

Figure EV1. $PGF_{2\alpha}$ and PGE_2 generation in retinas from OIR mice.

Retinas were pooled from six mice per group and subjected to PG analysis through LC/MS. Data were analyzed by the unpaired Student's *t*-test ($n = 3$). n.s. stands for "not significant." Data are represented as mean ± SEM.

Figure EV2. *Ptgfr* knockout in vascular ECs attenuates angiogenesis in OIR mice.

A Generation of endothelial cell (EC)-specific *Ptgfr* knockout mice. *Ptgfr*-floxed mice were crossed with VE-cadherin-Cre mice to create EC-specific *Ptgfr* knockout mice (CKO-V).

B *Ptgfr* mRNA levels in retinal microvessels of CKO-V and control mice ($n = 4$).

C Representative images of OIR retinas from CKO-V and control mice on postnatal day 17. The green color shows the isolectin B_4-stained vessels. The second row panels are the enlarged images of white boxes from the first row panels. The third row images show neovascular tufts (NV, white) and the fourth row images show the vaso-obliteration (VO, white) area.

D Quantitation of oxygen-induced retinal neovascularization in CKO-V and control mice ($n = 12$).

E Quantitation of retinal vaso-obliteration in CKO-V and control mice ($n = 12$).

Data information: n.s. stands for "not significant." Data were analyzed by unpaired Student's *t*-test (B), Mann–Whitney test (D, E). Scale bars: 500 µm (unmagnified image), 150 µm (magnified image). Data are represented as mean ± SEM.
Source data are available online for this figure.

▶

 © 2022 The Authors

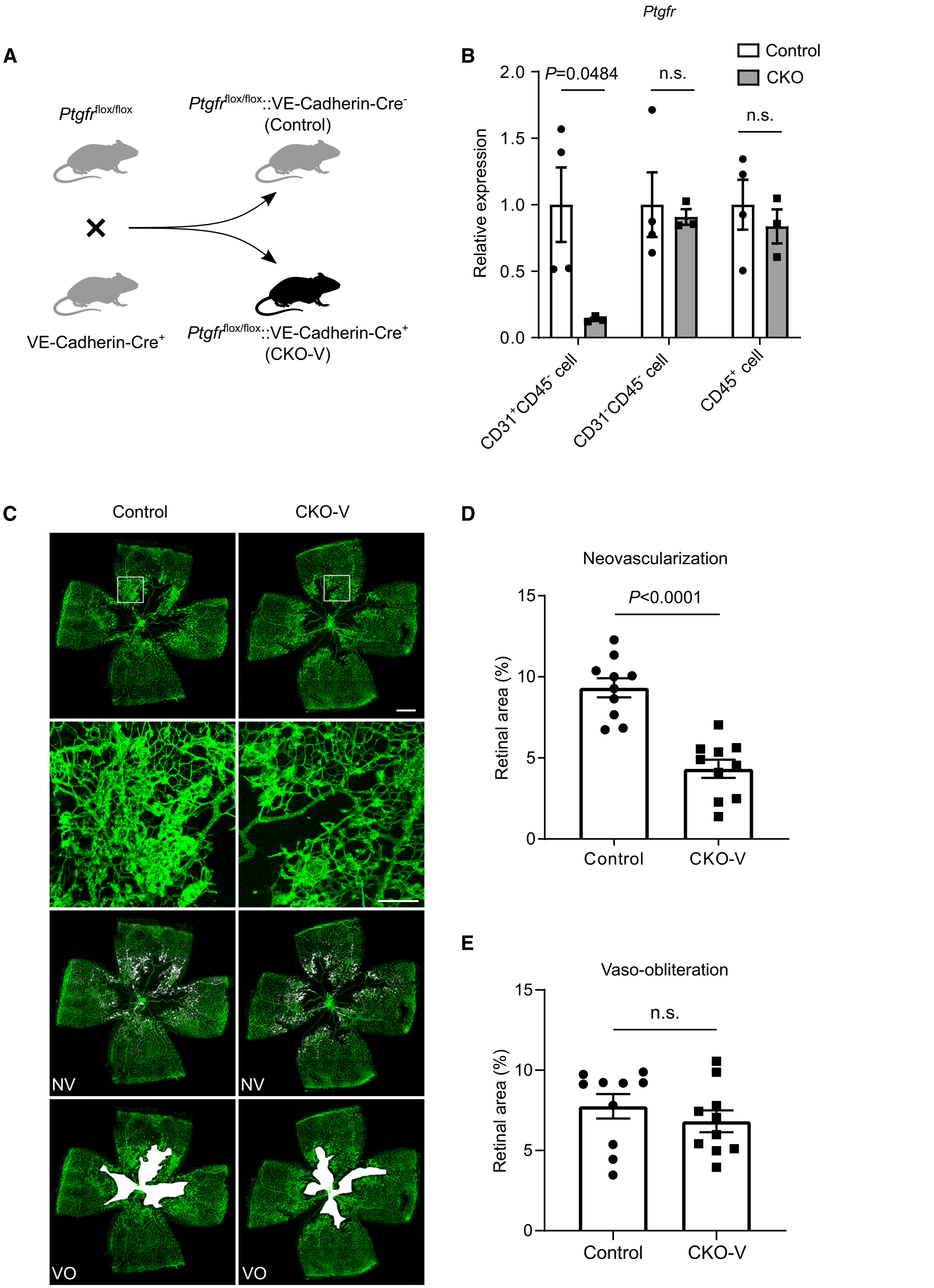
A
Ptgfr flox/flox
Ptgfr flox/flox::VE-Cadherin-Cre-
(Control)
×
VE-Cadherin-Cre+
Ptgfr flox/flox::VE-Cadherin-Cre+
(CKO-V)
B
Ptgfr
Control
CKO
P=0.0484
n.s.
n.s.
Relative expression
2.0
1.5
1.0
0.5
0.0
CD31+CD45- cell
CD31-CD45- cell
CD45+ cell
C
Control
CKO-V
NV
NV
VO
VO
D
Neovascularization
P<0.0001
Retinal area (%)
15
10
5
0
Control
CKO-V
E
Vaso-obliteration
n.s.
Retinal area (%)
15
10
5
0
Control
CKO-V

Figure EV2.

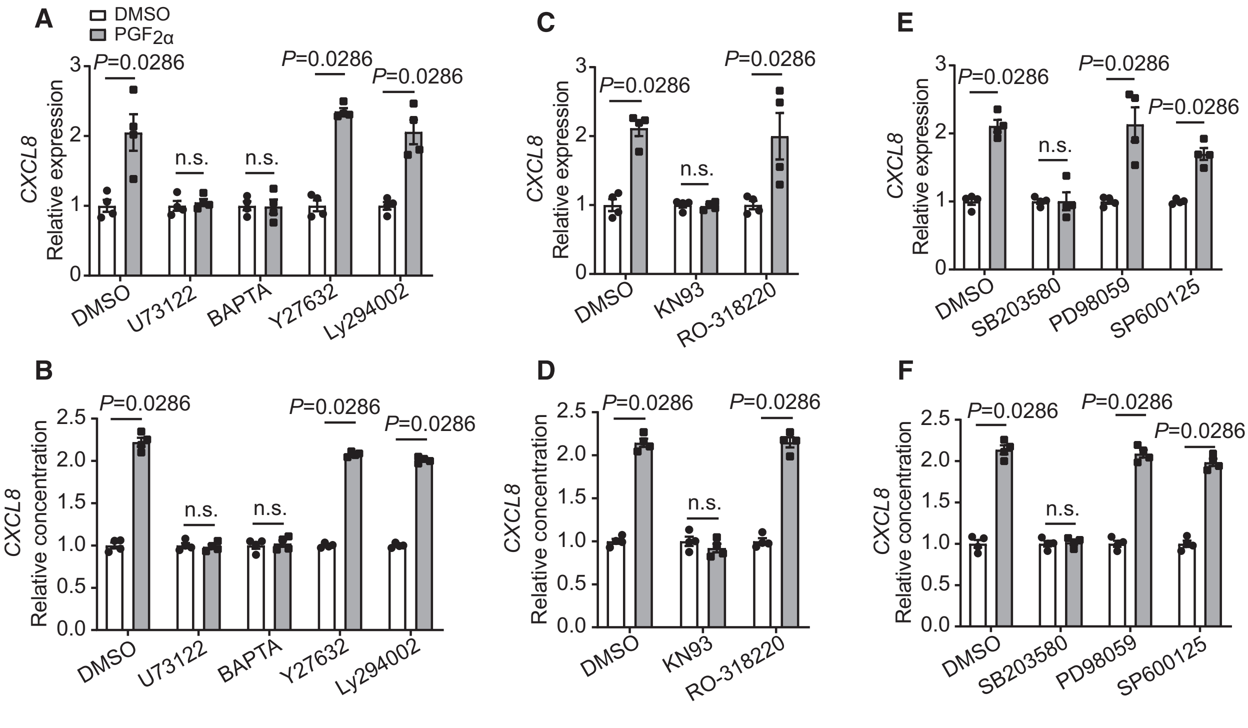

Figure EV3. Effect of different chemical inhibitors on $PGF_{2\alpha}$-induced *CXCL8* expression in HRMECs.

A, B Effect of U73122, BAPTA, Y27632, and Ly294002 treatment on $PGF_{2\alpha}$-induced *CXCL8* gene expression in HRMECs and secretion in culture medium (n = 4).

C, D Effect of KN93 and RO-318220 treatment on $PGF_{2\alpha}$-induced *CXCL8* gene expression in HRMECs and secretion in culture medium (n = 4).

E, F Effect of SB203580, PD98059, and SP600125 treatment on $PGF_{2\alpha}$-induced *CXCL8* gene expression in HRMECs and secretion in culture medium (n = 4).

Data information: n.s. stands for “not significant.” Data were analyzed by the Mann–Whitney test (A, B, C, D, E, F). Data are represented as mean ± SEM.

© 2022 The Authors

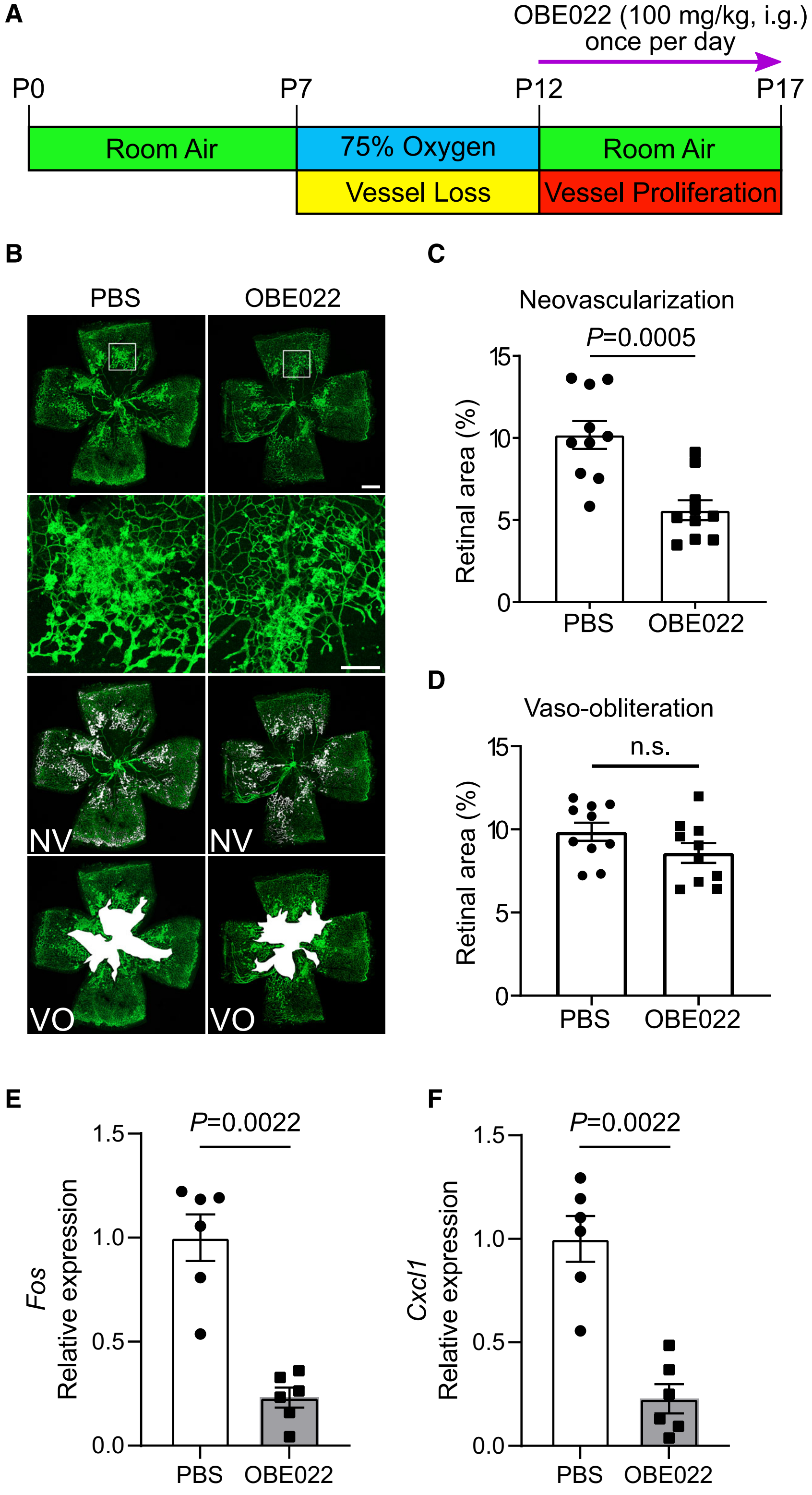

Figure EV4. PGTFR inhibitor OBE022 attenuates retinal angiogenesis in OIR mice.

A Schematic diagram of OBE022 administration in OIR mice. i.p. stands for intraperitoneally injected.

B Representative images of OIR retinas in mice with or without OBE022 treatment. The green color shows the isolectin B_4-stained vessels, the second row panels display the enlarged images of white boxes in the first row panels, the third row images show neovascular tufts (NV, white), and the fourth row images show the vaso-obliteration (VO, white) area.

C Quantitation of oxygen-induced retinal neovascularization with or without OBE022 treatment ($n = 10$).

D Quantitation of retinal vaso-obliteration with or without OBE022 treatment ($n = 10$).

E Effect of OBE022 on retinal *Fos* expression in OIR mice on postnatal day 16 ($n = 6$).

F Effect of OBE022 on retinal *Cxcl1* expression in OIR mice on postnatal day 16 ($n = 6$).

Data information: Data were analyzed by Mann–Whitney test (C, D, E, F). Scale bar: 500 μm (unmagnified image), 150 μm (magnified image). Data are represented as mean ± SEM.
Source data are available online for this figure.
