# Supplementary material for: PGF2α facilitates pathological retinal angiogenesis by modulating endothelial FOS‐driven ELR + CXC chemokine expression
Source: EMBO Mol Med. 2022 Dec 13;15(1):e16373. doi: 10.15252/emmm.202216373 (PMC9832840; doi:10.15252/emmm.202216373)
Supplement: Supplementary file 3 — Table EV1 [file EMMM-15-e16373-s005.docx]

**Table EV1 Patient Characteristics**

|  | **no DR (n=24)** | **NPDR (n=20)** | **PDR (n=24)** | ***P* value** |
| --- | --- | --- | --- | --- |
| Gender (male/female) | 14/10 | 11/9 | 14/10 | 0.9684 |
| Age (years) | 66.49±4.794 | 64.75±3.932 | 63.88±6.278 | 0.1204 |
| BMI (kg/m^2^) | 25.59±2.432 | 26.44±3.509 | 26.11±4.381 | 0.5953 |
| Duration (years) | 18.79±2.226 | 18.1±6.912 | 16.67±4.788 | 0.0642 |
| HbA_1c_ (%) | 8.342±1.368 | 8.235±1.744 | 7.679±1.407 | 0.2455 |
| TXB_2_ (ng/mL) | 18.269±32.177 | 17.024±32.627 | 18.581±20.806 | 0.6614 |

Data are expressed as the mean ± SD. Statistical tests were performed with the Chi-square test for gender and the Kruskal-Wallis test for other characteristics.
