# Supplementary material for: PGF2α facilitates pathological retinal angiogenesis by modulating endothelial FOS‐driven ELR + CXC chemokine expression
Source: EMBO Mol Med. 2022 Dec 13;15(1):e16373. doi: 10.15252/emmm.202216373 (PMC9832840; doi:10.15252/emmm.202216373)
Supplement: Supplementary file 5 — PDF+ [file EMMM-15-e16373-s002.pdf]

Article

SOURCE DATA | TRANSPARENT PROCESS | OPEN ACCESS

EMBO Molecular Medicine

$PGF_{2\alpha}$ facilitates pathological retinal angiogenesis by modulating endothelial FOS-driven ELR^+ CXC chemokine expression

Yan Zhao[1,2], Yi Lei[3,†], Huying Ning[1,†], Yaqiang Zhang[4], Guilin Chen[1], Chenchen Wang[1,2], Qiangyou Wan[2], Shumin Guo[1], Qian Liu[1], Ruotian Xie[1], Yujuan Zhuo[1], Shuai Yan[2], Jing Zhao[5], Fengjiang Wei[5], Lu Wang[1], Xiaohong Wang[1], Weidong Li[5], Hua Yan[3,*] & Ying Yu[1,**]

Abstract

The pathological retinal angiogenesis often causes blindness. Current anti-angiogenic therapy for proliferative retinopathy targets the vascular endothelial growth factor (VEGF), but many patients do not radically benefit from this therapy. Herein, we report that circulating prostaglandin (PG) $F_{2\alpha}$ metabolites were increased in type 2 diabetic patients with proliferative retinopathy, and the $PGF_{2\alpha}$ receptor (*Ptgfr*) was upregulated in retinal endothelial cells (ECs) from a mouse model of oxygen-induced retinopathy (OIR). Further, disruption of the PTGFR receptor in ECs attenuated OIR in mice. $PGF_{2\alpha}$ promoted the proliferation and tube formation of human retinal microvascular endothelial cells (HRMECs) via the release of ELR^+ CXC chemokines, such as CXCL8 and CXCL2. Mechanistically, the $PGF_{2\alpha}$/PTGFR axis potentiated ELR^+ CXC chemokine expression in HRMECs through the G_q/CAMK2G/p38/ELK-1/FOS pathway. Upregulated FOS-mediated ELR^+ CXC chemokine expression was observed in retinal ECs from PDR patients. Moreover, treatment with PTGFR inhibitor lessened the development of OIR in mice in a CXCR2-dependent manner. Therefore, inhibition of PTGFR may represent a new avenue for the treatment of retinal neovascularization, particularly in PDR.

Keywords anti-angiogenic therapy; pathological retinal angiogenesis; $PGF_{2\alpha}$
Subject Categories Neuroscience; Vascular Biology & Angiogenesis
DOI 10.15252/emmm.202216373 | Received 26 May 2022 | Revised 11 November 2022 | Accepted 15 November 2022
EMBO Mol Med (2022) e16373

Introduction

Pathological retinal neovascularization, characterized by an aberrantly proliferating vascular tuft structure, is the hallmark of retinopathy of prematurity and proliferative diabetic retinopathy, which are the primary causes of severe vision loss in children and adults in developed countries (Lee *et al*, 2015; Seery *et al*, 2020). Vascular endothelial growth factor (VEGF), a pleiotropic proangiogenic factor, is believed to facilitate the development of these conditions, rendering it a valuable therapeutic target (Penn *et al*, 2008; Seery *et al*, 2020). Although anti-VEGF therapy is beneficial in proliferative retinopathy (Mintz-Hittner *et al*, 2011; Wells *et al*, 2015), the short-lived effects of anti-VEGF agents require frequent intervention (Rodrigues *et al*, 2009). Moreover, a considerable number of patients exhibit poor/no response to VEGF-targeting drugs (Yang *et al*, 2016). These clinical observations implicate other signaling pathways in the promotion of retinal angiogenesis through the increased local production of various pro-angiogenic factors or pro-inflammatory lipid mediators (Rezzola *et al*, 2020; Wang *et al*, 2020). Therefore, it is essential to identify additional factors involved in the pathogenesis of proliferative retinal neovascularization.

Prostaglandins (PGs), a group of inflammatory lipid mediators, play a crucial role in inflammation and the maintenance of tissue homeostasis. They are enzymatically derived from arachidonic acid through cyclooxygenases (COXs) and downstream PG synthases. Nonsteroidal anti-inflammatory drugs (NSAIDs) exert analgesic and fever-reducing effects by inhibiting COX activity and decreasing PG production. The COX-2 isoform is expressed in retinal blood vessels and astrocytes of human diabetic retinopathy patients, as well as in rodent models of ischemic proliferative retinopathy; inhibition of COX-2 suppresses retinal angiogenesis in experimental rodent

1 Department of Pharmacology, Tianjin Key Laboratory of Inflammatory Biology, Center for Cardiovascular Diseases, Key Laboratory of Immune Microenvironment and Disease (Ministry of Education), The Province and Ministry Co-sponsored Collaborative Innovation Center for Medical Epigenetics, Tianjin Medical University, Tianjin, China
2 CAS Key Laboratory of Nutrition, Metabolism and Food Safety, Shanghai Institute of Nutrition and Health, University of Chinese Academy of Sciences, Chinese Academy of Sciences, Shanghai, China
3 Department of Ophthalmology, Tianjin Medical University General Hospital, Tianjin, China
4 Key Laboratory of Brain Functional Genomics, Ministry of Education and Shanghai, School of Life Science, East China Normal University, Shanghai, China
5 Department of Genetics, School of Basic Medical Sciences, Tianjin Medical University, Tianjin, China
*Corresponding author. Tel: +86 22 83336627; E-mail: yuying@tmu.edu.cn
**Corresponding author. Tel: +86 22 60817010; E-mail: zyyyanhua@tmu.edu.cn
†These authors contributed equally to this work as first authors

© 2022 The Authors. Published under the terms of the CC BY 4.0 license.

models (Sennlaub et al, 2003; Wang et al, 2020). Moreover, topical administration of penetration-prone nepafenac alleviates oxygen-induced retinopathy (OIR) in rats more effectively than other injectable NSAIDs (Yanni et al, 2010). These observations suggest that COX-produced PGs may be involved in the progression of proliferative retinopathy. PGs exert their physiological effects by binding to specific G protein-coupled receptor(s). $PGF_{2\alpha}$ facilitates both labor onset and accomplishment (Li et al, 2020) and decreases intraocular pressure by regulating ocular uveoscleral outflow (Klimko & Sharif, 2019) through the F-prostanoid receptor (PTGFR). Therefore, $PGF_{2\alpha}$ analogs are widely employed for glaucoma therapy (Klimko & Sharif, 2019). Interestingly, circulating $PGF_{2\alpha}$ levels are markedly higher in diabetic patients than in healthy controls (Helmersson et al, 2004; Basu et al, 2005). Furthermore, $PGF_{2\alpha}$ stimulates fasting-induced hepatic glucose production in fasting and diet-induced diabetic mice. Polymorphisms in the promoter region of aldose reductase-one endogenous $PGF_{2\alpha}$ synthases (Bresson et al, 2012) are associated with susceptibility to diabetic microvascular complications, including retinopathy (Demaine, 2003). However, the exact role of $PGF_{2\alpha}$ in proliferative diabetic retinopathy (PDR) remains to be determined.

In the present study, we found that $PGF_{2\alpha}$ generation was markedly increased in PDR patients as well as in a mouse OIR model. The PTGFR receptor is expressed in retinal endothelial cells (ECs) during the vessel proliferative stage in OIR mice. *PTGFR* deletion in ECs mitigated oxygen-induced retinopathy in mice mainly by suppressing EC proliferation. Mechanistically, the $PGF_{2\alpha}$/PTGFR axis promoted HRMEC proliferation through CAMK2G/p38/ELK-1/FOS pathway-mediated ELR^+ CXC chemokine secretion. Blockade of the PTGFR receptor by AL8810 attenuated retinal neovascularization in OIR mice. Therefore, inhibition of the PTGFR receptor may be a novel approach for the treatment of PDR.

Results

$PGF_{2\alpha}$/PTGFR axis found to be upregulated in pathological neovessels in OIR

Circulating $PGF_{2\alpha}$ levels, as measured by its stable metabolite 15-keto-dihydro-$PGF_{2\alpha}$, are increased in patients with type 2 diabetes mellitus (Helmersson et al, 2004). To assess the role of the $PGF_{2\alpha}$/PTGFR axis in pathological retinal vascular proliferation, we quantified serum 15-keto-dihydro-$PGF_{2\alpha}$ in type 2 diabetic patients without diabetic retinopathy (no DR), with non-proliferative diabetic retinopathy (NPDR), and with proliferative diabetic retinopathy (PDR). To exclude the potential effect of aspirin and other NSAIDs, only serum samples with thromboxane $(TX)B_2$ levels greater than 1,000 pg/ml were further analyzed (Good et al, 2015). A total of 68 samples were obtained: 24 from patients with no DR, 20 from NPDR patients, and 24 from PDR patients, with no substantial differences in sex, age, BMI, duration of diabetes, percentage of glycated hemoglobin, or serum TXB_2 levels between the three groups (Table EV1). As shown in Fig 1A, serum 15-keto-dihydro-$PGF_{2\alpha}$ levels were markedly higher in patients with PDR than in those without retinopathy and those with NPDR. No statistical difference in serum 15-keto-dihydro-$PGF_{2\alpha}$ levels was detected between patients with NPDR and those without DR. In OIR mice with proliferative retinal neovascularization (Connor et al, 2009; Fig 1B), $PGF_{2\alpha}$ production was mildly increased in retinal tissues in the vessel proliferation stage during postnatal days 12–17 (Figs 1C and EV1), along with elevated expression of the main retinal $PGF_{2\alpha}$ synthase, *Akr1a1* (Fig 1D), when compared with the corresponding levels in normoxic littermates. Interestingly, PTGFR expression was increased in OIR retinas, by more than 11-fold on postnatal day 16, compared with the corresponding expression level in controls (Fig 1E). We then explored PTGFR distribution in the retinal vascular and neuronal layers by laser capture microdissection on postnatal day 16 and magnetic-activated cell sorting (Fig 1F and G). *Ptgfr* expression was relatively abundant in retinal vessels and $CD31^+CD45^-$ endothelial cells and drastically increased in OIR retinas compared with the corresponding expression level in normal retinas (Fig 1F and G); this consequently highlighted the role of the $PGF_{2\alpha}$/PTGFR axis of retinal ECs in the development of pathological retinal angiogenesis.

Ablation of PTGFR in vascular ECs attenuated pathological angiogenesis in OIR mice

To investigate the role of the $PGF_{2\alpha}$/PTGFR axis in proliferative retinopathy, we created EC-specific *PTGFR* knockout mice (CKO-T) by crossing *Ptgfr*-floxed mice (Wang et al, 2018) with Tie2-Cre transgenic mice (Kisanuki et al, 2001; Fig 2A). Quantitative PCR confirmed *Ptgfr* deletion in the retinal ECs of CKO-T mice (Fig 2B). CKO-T mice exhibited a significant reduction of oxygen-induced

Figure 1. Activation of the $PGF_{2\alpha}$-PTGFR axis in pathological retinal angiogenesis. ▶

A Serum levels of 15-keto-dihydro-$PGF_{2\alpha}$ in type 2 diabetic patients without diabetic retinopathy (no DR), with non-proliferative diabetic retinopathy (NPDR), and with proliferative diabetic retinopathy (PDR; n = 20–24).
B Schematic diagram of the OIR mouse model. Mice on postnatal day 7 were placed in a 75% oxygen container for 5 days and were then transferred to a normal environment on postnatal day 12 for another 5 days to induce retinal vessel proliferation.
C Retinal $PGF_{2\alpha}$ production in OIR mice during postnatal days 12–17 (n = 4).
D mRNA expression of $PGF_{2\alpha}$ synthases in the retinas of OIR mice during postnatal days 12–17 (n = 4).
E mRNA expression of *Ptgfr* in the retinas of OIR mice during postnatal days 12–17 (n = 6–8).
F mRNA expression of *Ptgfr* in each retinal layer from normoxic and OIR mice on postnatal day 16 (n = 6). The left-hand image displays representative cross-sections from OIR and normoxic retinas, with isolectin B_4-stained vessels in green, DAPI-stained nuclei in blue, and LCM isolation locations circled by white dashed circles. INL, inner nuclear layer; ONL, outer nuclear layer; RGC, retinal ganglion cells.
G *Ptgfr* mRNA expression in retinal endothelial cells ($CD31^+CD45^-$) sorted by MACS from normoxic and OIR mice on postnatal day 16 (n = 5).

Data information: n.s. stands for "not significant." Data were analyzed by Kruskal–Wallis test with Dunn's multiple comparisons test (A), two-way ANOVA with Tukey's multiple comparisons test (C, D, E, F), or Mann–Whitney test (G). Scale bar: 20 μm. Data are represented as mean ± SEM.
Source data are available online for this figure.

 © 2022 The Authors

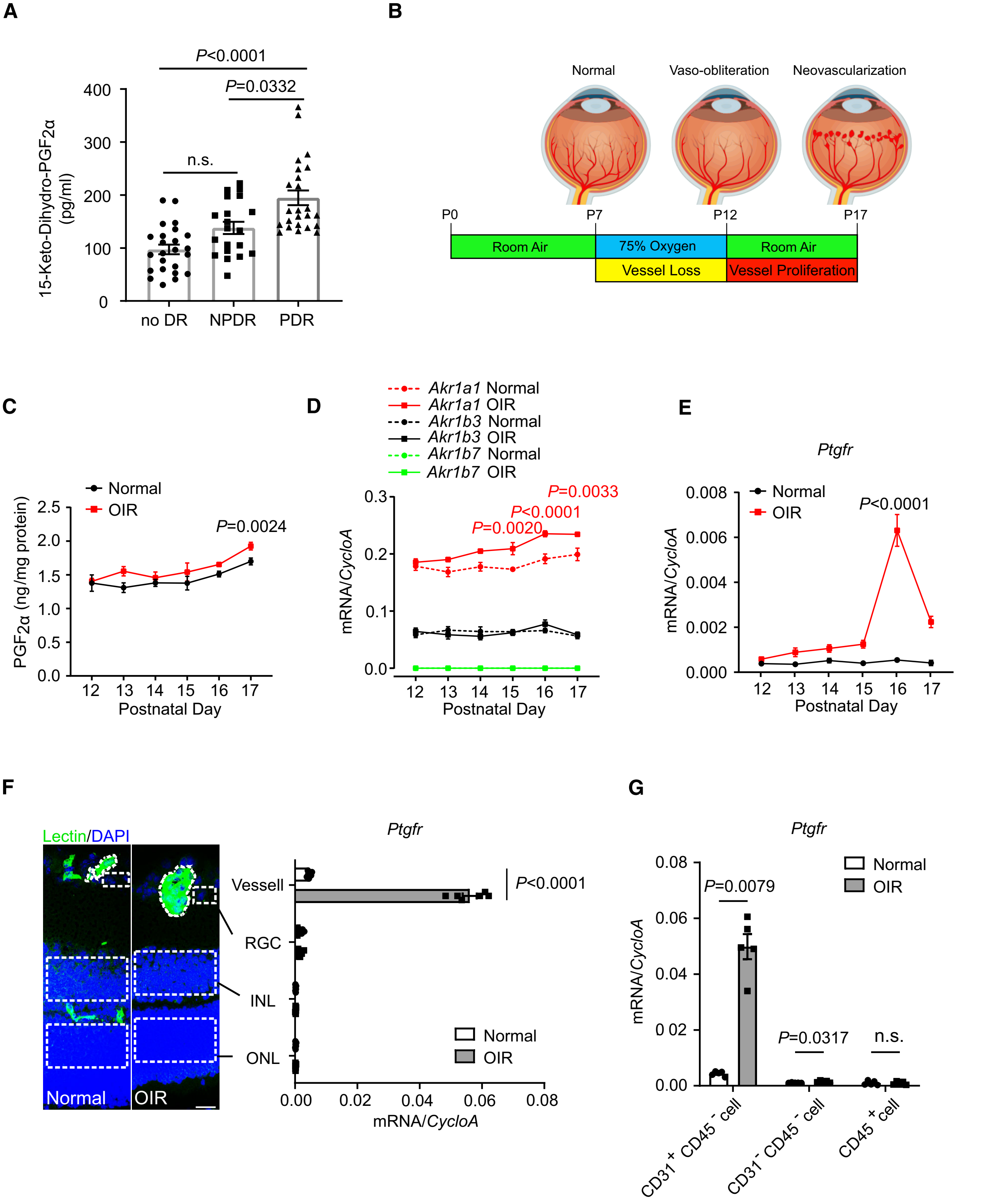

Figure 1.

neovascularization in retinas on postnatal day 17 as compared with their littermate controls (Control: 10.96 ± 0.90%; CKO-T: 4.98 ± 0.57%; $P < 0.0001$, Fig 2C and D), with comparable vaso-obliteration (Fig 2E). To rule out non-endothelial gene excision mediated by Tie2 promoter-driven Cre recombinase, VE-Cadherin-Cre transgenic mice (Alva *et al*, 2006) were crossed to *Ptgfr*-floxed

mice and subjected to phenotypic characterization (Fig EV2A). Similarly, more than 50% reduction of neovascularization was observed in VE-Cadherin-Cre-derived endothelial *PTGFR*-deficient mice (CKO-V), compared with that in control mice (Fig EV2B–E). *PTGFR* deficiency had no notable influence on retinal vascular development in neonatal mice (Appendix Fig S1A–D). Therefore, these results

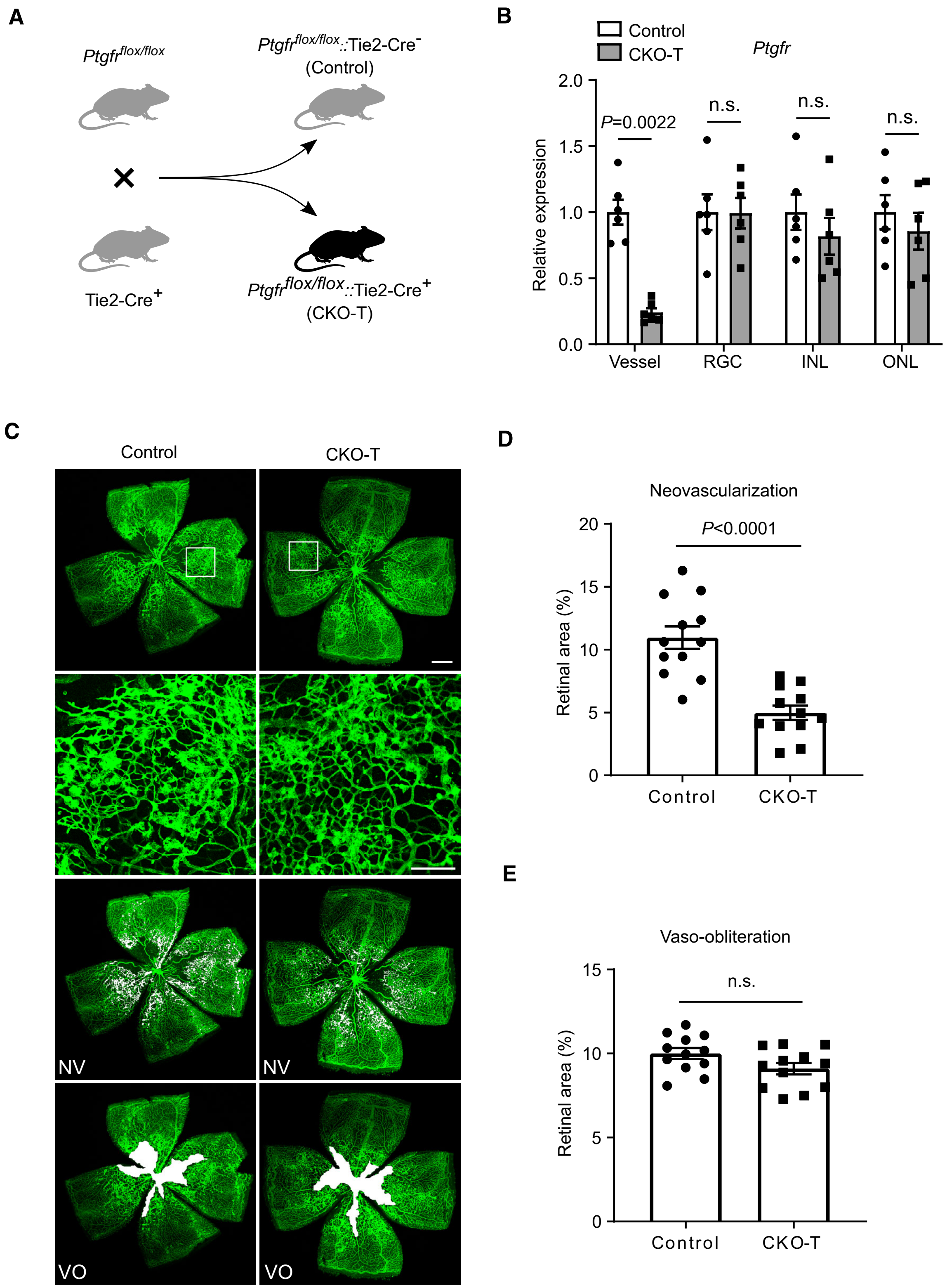

Figure 2.

 © 2022 The Authors

Figure 2. *Ptgfr* knockout in vascular ECs attenuates angiogenesis in OIR mice.

A Generation of endothelial cell (EC)-specific *Ptgfr* knockout mice. *Ptgfr*-floxed mice were crossed with Tie2-Cre mice to create EC-specific *Ptgfr* knockout mice (CKO-T).
B *Ptgfr* mRNA levels in retinal microvessels of CKO-T and control mice ($n = 6$). INL, inner nuclear layer; ONL, outer nuclear layer; RGC, retinal ganglion cells.
C Representative images of OIR retinas from CKO-T and control mice on postnatal day 17. The green color shows the isolectin B_4-stained vessels. The second row panels are the enlarged images of white boxes from the first row panels. The third row images show neovascular tufts (NV, white) and the fourth row images show the vaso-obliteration (VO, white) area.
D Quantitation of oxygen-induced retinal neovascularization in CKO-T and control mice ($n = 12$).
E Quantitation of retinal vaso-obliteration in CKO-T and control mice ($n = 12$).

Data information: n.s. stands for "not significant." Data were analyzed by unpaired Student's *t*-test (B), Mann–Whitney test (D, E). Scale bars: 500 μm (unmagnified image), 150 μm (magnified image). Data are represented as mean ± SEM.
Source data are available online for this figure.

indicated that the $PGF_{2\alpha}$/PTGFR axis mediates oxygen-induced proliferative retinopathy in mice.

$PGF_{2\alpha}$ promoted HRMEC proliferation *in vitro* as well as vascular sprouting *ex vivo*

To further define the involvement of $PGF_{2\alpha}$ in proliferative retinopathy, we examined the migration, proliferation, and tube formation of human retinal microvascular endothelial cells (HRMECs) *in vitro*, as well as vascular sprouting using mouse aortic rings *ex vivo*. We found that $PGF_{2\alpha}$ treatment facilitated the migration of HRMECs toward wound scratches (Fig 3A and B) and promoted the proliferation of HRMECs in a dose-dependent manner (Fig 3C). The BrdU incorporation assay further confirmed markedly increased proliferation of $PGF_{2\alpha}$-treated HRMECs (Fig 3D and E). $PGF_{2\alpha}$ also substantially enhanced HRMEC tube formation, with a 1.2-fold increase in tube length, a 1.3-fold increase in the number of junctions, as well as a 1.6-fold and 1.2-fold increase in the number of meshes and percentage of mesh area (Fig 3F–J). Similarly, $PGF_{2\alpha}$ stimulated mouse aortic ring sprouting *ex vivo* (Fig 3K and L, over 3-fold increase at day 5).

$PGF_{2\alpha}$ promoted HRMEC proliferation, as well as retinal angiogenesis, in mice through the release of ELR^+ CXC chemokines

To investigate the molecular mechanism through which $PGF_{2\alpha}$ promotes the proliferation of retinal microvascular ECs, we analyzed the gene-expression profile of $PGF_{2\alpha}$-treated HRMECs using RNA-seq. As shown in Fig 4A, a total of 67 genes were significantly upregulated in HRMECs after $PGF_{2\alpha}$ treatment. Gene Ontology (GO) enrichment of biological processes revealed that these upregulated genes in $PGF_{2\alpha}$-treated HRMECs were most significantly enriched in chemokine-related signaling pathways (Fig 4B). Chemokines are mediators of neovascularization, with their angiogenic or angiostatic function, and all CXC chemokines with the glutamic acid-leucine-arginine (ELR) motif are potent promoters of angiogenesis (Keeley *et al*, 2008). Interestingly, four ELR^+ CXC chemokines, including *CXCL8*, *CXCL2*, *CXCL3*, and *CXCL5*, were significantly upregulated in $PGF_{2\alpha}$-treated HRMECs (Fig 4A and C). Among them, *CXCL8* and *CXCL2* were highly expressed in HRMECs and markedly upregulated in response to $PGF_{2\alpha}$ treatment (Fig 4C). We failed to detect marked changes in the expression of *VEGFA* and its receptors in $PGF_{2\alpha}$-treated HRMECs (Fig 4A, Appendix Fig S2A and B) and in retinas from endothelial *PTGFR*-deficient mice (Appendix Fig S2C and D). Bioinformatics analyses of published transcriptome profiles (Gene Expression Omnibus Database dataset GSE94019; Lam *et al*, 2017) revealed that *CXCL8*, *CXCL2*, and *CXCL3* expression levels were dramatically increased in retinal microvascular ECs from patients with PDR compared with the corresponding levels in normal subjects (Fig 4D–G). Again, *CXCL8* and *CXCL2* were the primary upregulated ELR^+ CXC chemokines in human diabetic retinal ECs, with more than 32-fold and 43-fold increases in average mRNA expression, respectively (Appendix Table S1). In humans, ELR^+ CXC chemokines (i.e., CXCL8, CXCL2, and CXCL3) exert their functions through two receptors, namely CXCR1 and CXCR2. However, only *CXCR2*

Figure 3. $PGF_{2\alpha}$ promotes the migration, proliferation, and tube formation of human retinal microvascular endothelial cells (HRMECs) as well as aortic ring sprouting in culture.

A Representative images of scratch-induced migration of $PGF_{2\alpha}$-treated HRMECs. Yellow solid lines represent scratch locations, dashed lines represent post-migration locations, and arrows indicate migration direction.
B Quantitation of the cell migration distance in A ($n = 4$).
C Effect of $PGF_{2\alpha}$ treatment on HRMEC proliferation in a dose-dependent manner ($n = 6$).
D Representative images of staining for BrdU incorporation in $PGF_{2\alpha}$-treated HRMECs.
E Quantification of $BrdU^+$ cells in D ($n = 4$).
F Representative tube formation images of $PGF_{2\alpha}$-or vehicle (DMSO)-treated HRMECs.
G–J Quantitation of total tubule length, number of junctions, number of meshes, and percentage of mesh area in F ($n = 4$).
K Representative images of endothelial cell sprouting of $PGF_{2\alpha}$-treated mouse aortic rings. Yellow lines indicate the EC sprouting area.
L Quantification of the EC sprouting area in K ($n = 6$).

Data information: One data point represented the mean value of three technical replicates from one independent biological replicate. Data were analyzed by Mann–Whitney test (B, E, L), unpaired Student's *t*-test (G, H, I, J) or Kruskal–Wallis test with Dunn's multiple comparisons test (C). Scale bars: 100 μm (A) and 200 μm (F, K). Data are represented as mean ± SEM.
Source data are available online for this figure.

© 2022 The Authors

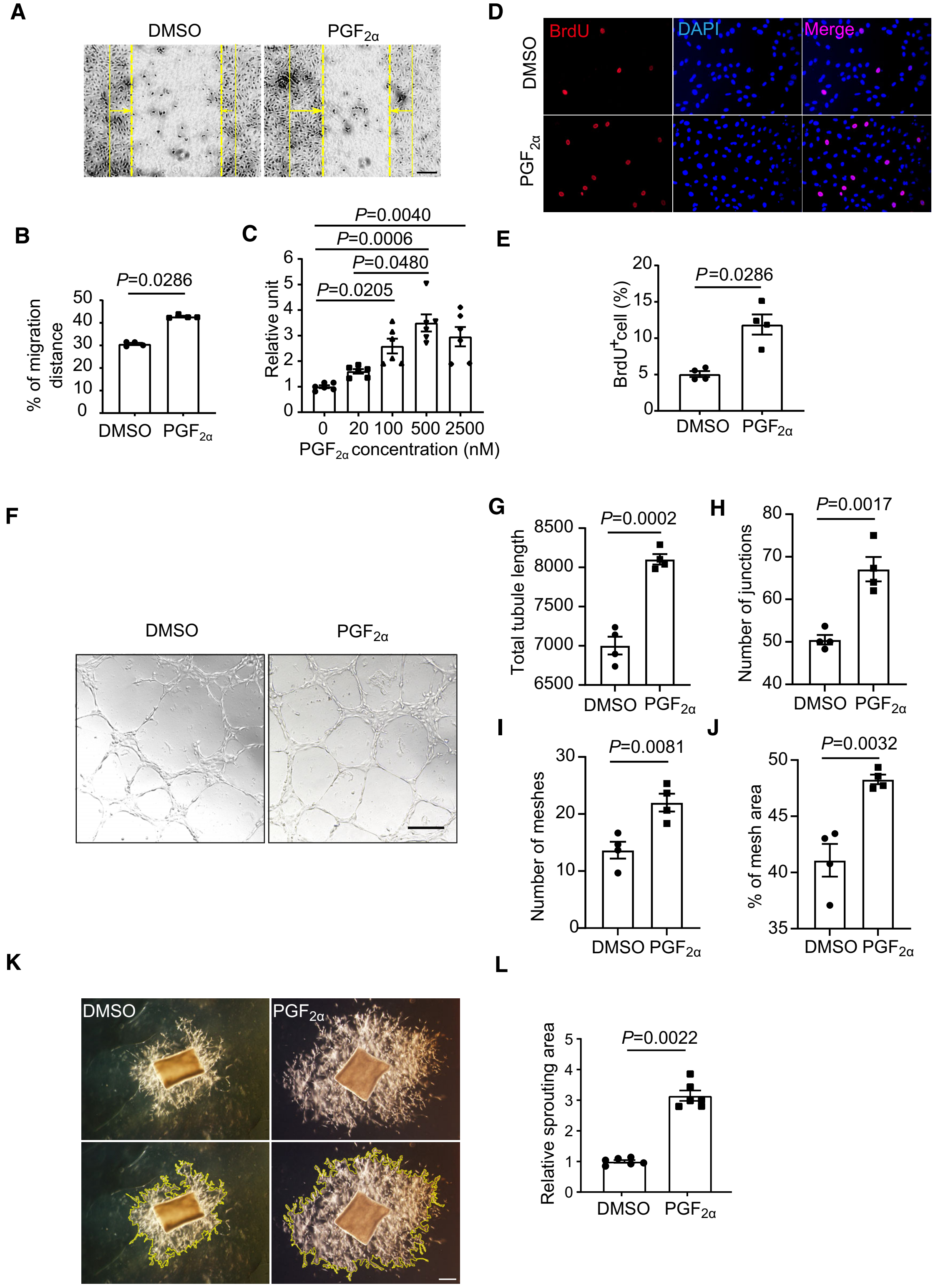

Figure 3.

was expressed in retinal ECs (Appendix Fig S3). We then sought to explore whether $PGF_{2\alpha}$-triggered retinal EC migration and proliferation is mediated via the ELR^+ CXC chemokine receptor CXCR2. As shown in Fig 4H–J, CXCR2 antagonist SB265610 attenuated the $PGF_{2\alpha}$-stimulated migration and proliferation of HRMECs. SB265610 also blunted the increased tube formation of HRMECs induced by $PGF_{2\alpha}$ treatment (Fig 4K and L). In mice, all ELR^+ CXC chemokine signaling occurs via CXCR2 (Keeley *et al*, 2008). CXCR2 antagonist

© 2022 The Authors

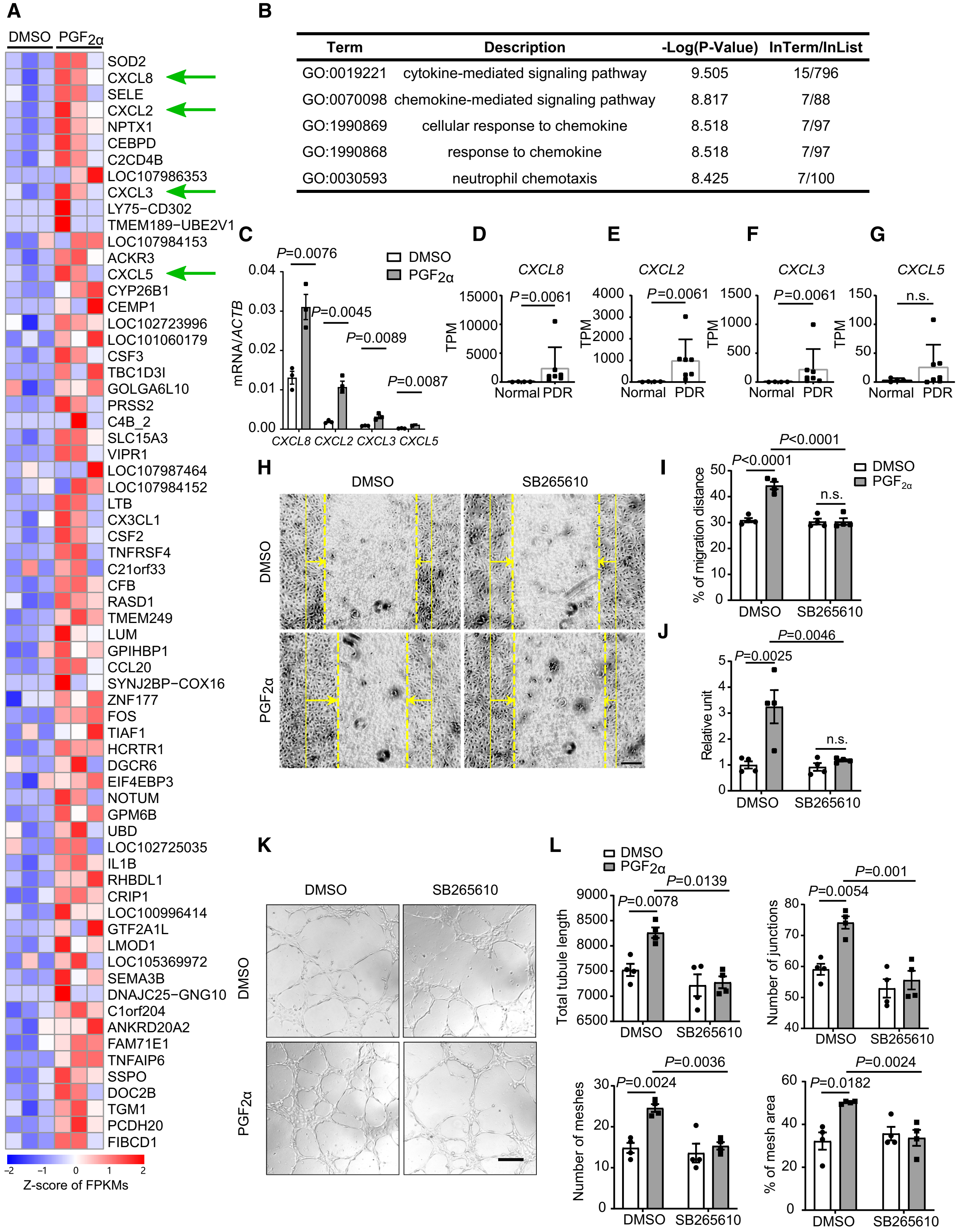

Term	Description	-Log(P-Value)	InTerm/InList
GO:0019221	cytokine-mediated signaling pathway	9.505	15/796
GO:0070098	chemokine-mediated signaling pathway	8.817	7/88
GO:1990869	cellular response to chemokine	8.518	7/97
GO:1990868	response to chemokine	8.518	7/97
GO:0030593	neutrophil chemotaxis	8.425	7/100

Figure 4.

Figure 4. $PGF_{2\alpha}$ promotes proliferation and tube formation of HRMECs through the upregulation of ELR^+ CXC chemokines.

A Heat map of upregulated genes in $PGF_{2\alpha}$-treated HRMECs ($n = 3$ samples).
B GO enrichment of the top five biological processes involving the upregulated genes in $PGF_{2\alpha}$-treated HRMECs.
C Effect of $PGF_{2\alpha}$ treatment on ELR^+ CXC chemokine expression in HRMECs ($n = 3$).
D–G Relative expression levels of enriched ELR^+ CXC chemokines in retinal microvascular ECs from patients with PDR ($n = 4$) compared with those from normal subjects ($n = 7$; Gene Expression Omnibus Database dataset GSE94019). TPM stands for "Transcripts per kilobase million."
H Effect of CXCR2 inhibitor SB265610 (1 μM) on $PGF_{2\alpha}$-stimulated HRMEC migration. The yellow solid line indicates the original position of the scratch, the dashed line indicates the position after cell migration, and the arrows indicate the migratory direction.
I Quantitation of scratch-induced HRMEC migration in H ($n = 4$).
J Effect of SB265610 (1 μM) on $PGF_{2\alpha}$-stimulated HRMEC proliferation ($n = 4$).
K Effect of CXCR2 inhibitor SB265610 (1 μM) on tube formation in $PGF_{2\alpha}$-treated HRMECs.
L Statistical graph of tube formation experiments in (K), comparing the total tubule length, number of junctions, number of meshes, and percentage of mesh area ($n = 4$).

Data information: n.s. stands for "not significant." Data were analyzed by unpaired Student's *t*-test (C), Mann–Whitney test (D, E, F, G), or two-way ANOVA with Tukey's multiple comparisons test (I, J, L). Data in (C, I, J, L) are represented as mean ± SEM. Data in (D–G) are presented as the mean + SD. Scale bar: 100 μm (H) and 200 μm (K).
Source data are available online for this figure.

SB265610 abolished $PGF_{2\alpha}$-induced EC sprouting of mouse aortic rings *ex vivo* (Fig 5A and B). There is no CXCL8 in mice, and mouse CXCL1 is the functional homolog of human CXCL8 (Hol *et al*, 2010; Grbic *et al*, 2012). We observed that *Cxcl1*, *Cxcl2*, *Cxcl3*, and *Cxcl5* were drastically upregulated in retinas from OIR mice during the proliferative stage during postnatal days 12–17 when compared with the corresponding expression levels in normoxic mice (Fig 5C). Remarkably, the expression of *Cxcl1* was significantly elevated more than 41-fold on postnatal day 16, with *Cxcl2* and *Cxcl3* also elevated, which coincided with the peak expression of *Ptgfr* during the vascular proliferation stage (Figs 1E and 5C). *PTGFR* deletion in retinal ECs markedly reduced the expression of these chemokines in retinas from OIR mice on postnatal day 16 (Fig 5D). We also tested whether forced expression of *Cxcl1* in retinas diminished the protective effect against OIR in CKO-T mice (Fig 5E). Indeed, intravitreal injection of lentivirus harboring *Cxcl1* restored retinal CXCL1 expression in CKO-T mice (Fig 5F and G), eliminating their resistance to oxygen-induced retinal neovascularization without affecting vaso-obliteration (Fig 5H–J). It was therefore determined that the $PGF_{2\alpha}$/PTGFR axis promotes retinal EC proliferation and angiogenesis in mice through the activation of ELR^+ CXC chemokines/CXCR2 signaling.

$PGF_{2\alpha}$/PTGFR axis found to drive ELR^+ CXC chemokine expression and tube formation of HRMECs through FOS

Whole-transcriptome analysis revealed five upregulated transcriptional factors in $PGF_{2\alpha}$-treated HRMECs (Fig 6A). Among these, FOS is known to regulate *CXCL8* expression by forming the activating protein-1 (AP-1) heterodimer with JUN (Bobrovnikova-Marjon *et al*, 2004). RT–PCR and Western blot confirmed FOS upregulation in $PGF_{2\alpha}$-treated HRMECs (Appendix Fig S4A–D). Our analysis of the GSE94019 dataset (Lam *et al*, 2017) revealed that FOS expression was also significantly elevated in the retinal ECs of PDR patients compared with the FOS expression in healthy controls (~ 5-fold induction, Fig 6B and Appendix Table S1). Furthermore, genetic knockdown of FOS (Appendix Fig S5A) or pharmacological inhibition of FOS/JUN dimerization attenuated $PGF_{2\alpha}$-induced expression and secretion of CXCL8 and CXCL2 in HRMECs (Fig 6C–F, Appendix Fig S5B and C). Inhibition of NF-κB and NFAT had no notable influence on $PGF_{2\alpha}$-induced CXCL8 expression in HRMECs (Fig 6E and F). Moreover, *FOS* knockdown abolished $PGF_{2\alpha}$-induced HRMEC migration and proliferation (Fig 6G–I), as well as tube formation (Fig 6J and K). As anticipated, the time window of *Fos* expression mirrored that of *Ptgfr* and *Cxcl1* in the retinas from OIR

Figure 5. Retinal *Cxcl1* overexpression abolishes decreased neovascularization in OIR retinas from CKO mice.

A Effect of CXCR2 inhibitor SB265610 (1 μM) on $PGF_{2\alpha}$-promoted sprouting of aortic rings. Yellow lines show the sprouting area.
B Quantitation of the sprouting areas in A ($n = 6$).
C mRNA expression of ELR^+ CXC chemokines in retinas from OIR mice during postnatal days 12–17 ($n = 4$–6).
D Effect of *PTGFR* deficiency in ECs on ELR^+ CXC chemokine expression in retinas from OIR mice on postnatal day 16 ($n = 6$).
E Schematic diagram of *Cxcl1*-expressing lentivirus transfection in OIR mice. Neonatal mice were injected with lentivirus on postnatal day 5, followed by oxygen treatment on day 7.
F Retinal CXCL1 protein-expression levels in *Cxcl1*-expressing lentivirus-injected CKO mice ($n = 3$).
G Quantitation of retinal CXCL1 protein in F ($n = 3$).
H Representative images of oxygen-induced retinal angiogenesis in *Cxcl1*-expressing lentivirus-injected CKO mice on postnatal day 17. Green represents the isolectin B_4-stained vessels, the second row panels display the enlarged images of white boxes in the first row panels, the third row images show neovascular tufts (NV, white), and the fourth row images show the vaso-obliteration (VO, white) area.
I Quantitation of oxygen-induced retinal neovascularization in H ($n = 8$).
J Quantitation of retinal vaso-obliteration in H ($n = 8$).

Data information: n.s. stands for "not significant." Data were analyzed by two-way ANOVA with Tukey's multiple comparisons test (B, C, D, G, I, J). Scale bar: 200 μm (A), 500 μm (H, unmagnified image), 150 μm (H, magnified image). Data are represented as mean ± SEM.
Source data are available online for this figure.

© 2022 The Authors

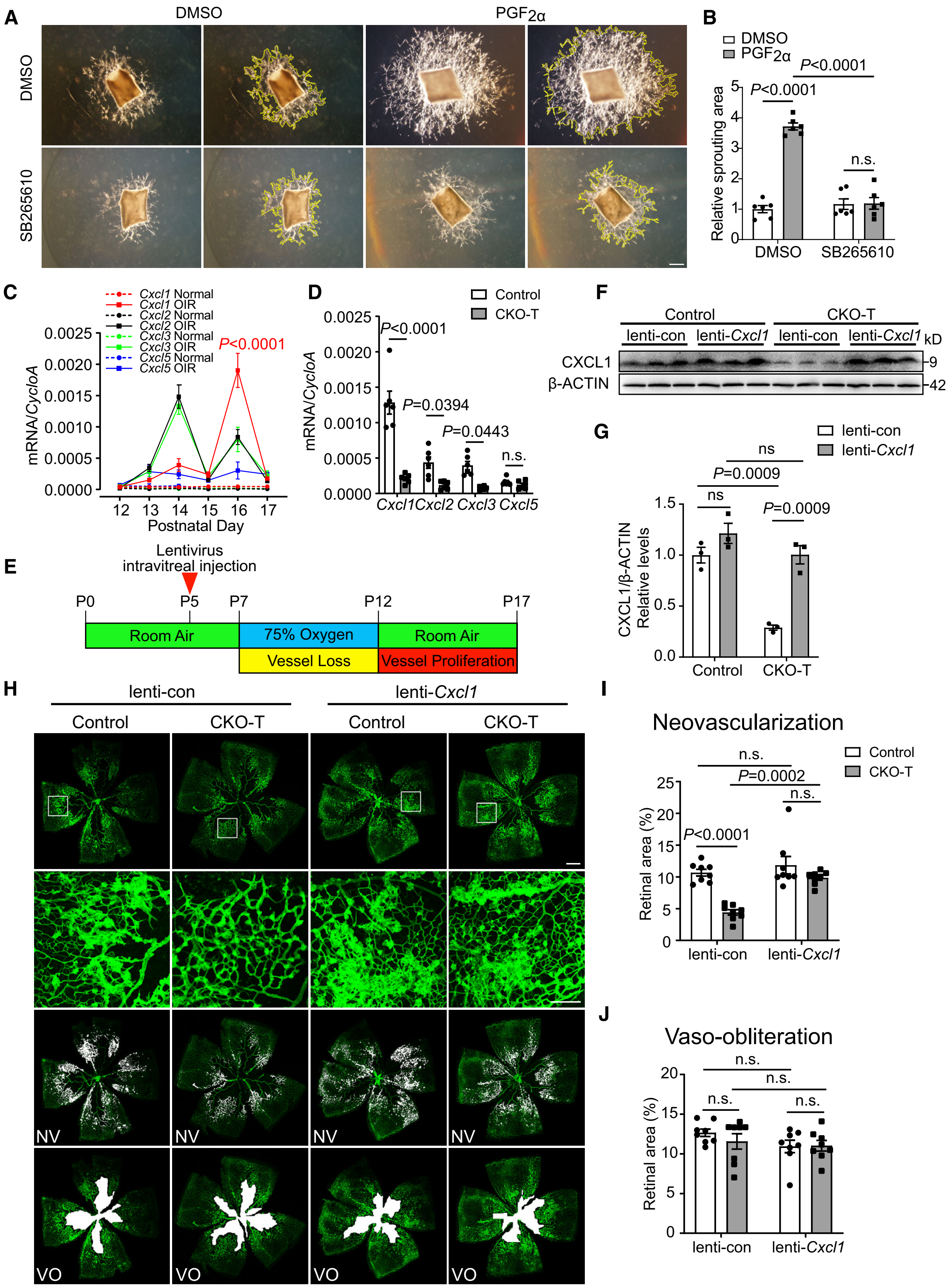
A
DMSO
PGF2α
DMSO
SB265610
B
DMSO
PGF2α
P<0.0001
P<0.0001
n.s.
Relative sprouting area
DMSO
SB265610
C
Cxcl1 Normal
Cxcl1 OIR
Cxcl2 Normal
Cxcl2 OIR
Cxcl3 Normal
Cxcl3 OIR
Cxcl5 Normal
Cxcl5 OIR
P<0.0001
mRNA/CycloA
Postnatal Day
D
Control
CKO-T
P<0.0001
P=0.0394
P=0.0443
n.s.
mRNA/CycloA
Cxcl1 Cxcl2 Cxcl3 Cxcl5
E
Lentivirus intravitreal injection
P0
P5
P7
P12
P17
Room Air
75% Oxygen
Room Air
Vessel Loss
Vessel Proliferation
F
Control
CKO-T
lenti-con
lenti-Cxcl1
lenti-con
lenti-Cxcl1
kD
CXCL1
β-ACTIN
9
42
G
lenti-con
lenti-Cxcl1
ns
P=0.0009
ns
P=0.0009
CXCL1/β-ACTIN Relative levels
Control
CKO-T
H
lenti-con
lenti-Cxcl1
Control
CKO-T
Control
CKO-T
NV
VO
I
Neovascularization
Control
CKO-T
n.s.
P=0.0002
n.s.
P<0.0001
Retinal area (%)
lenti-con
lenti-Cxcl1
J
Vaso-obliteration
n.s.
n.s.
n.s.
n.s.
Retinal area (%)
lenti-con
lenti-Cxcl1

Figure 5.

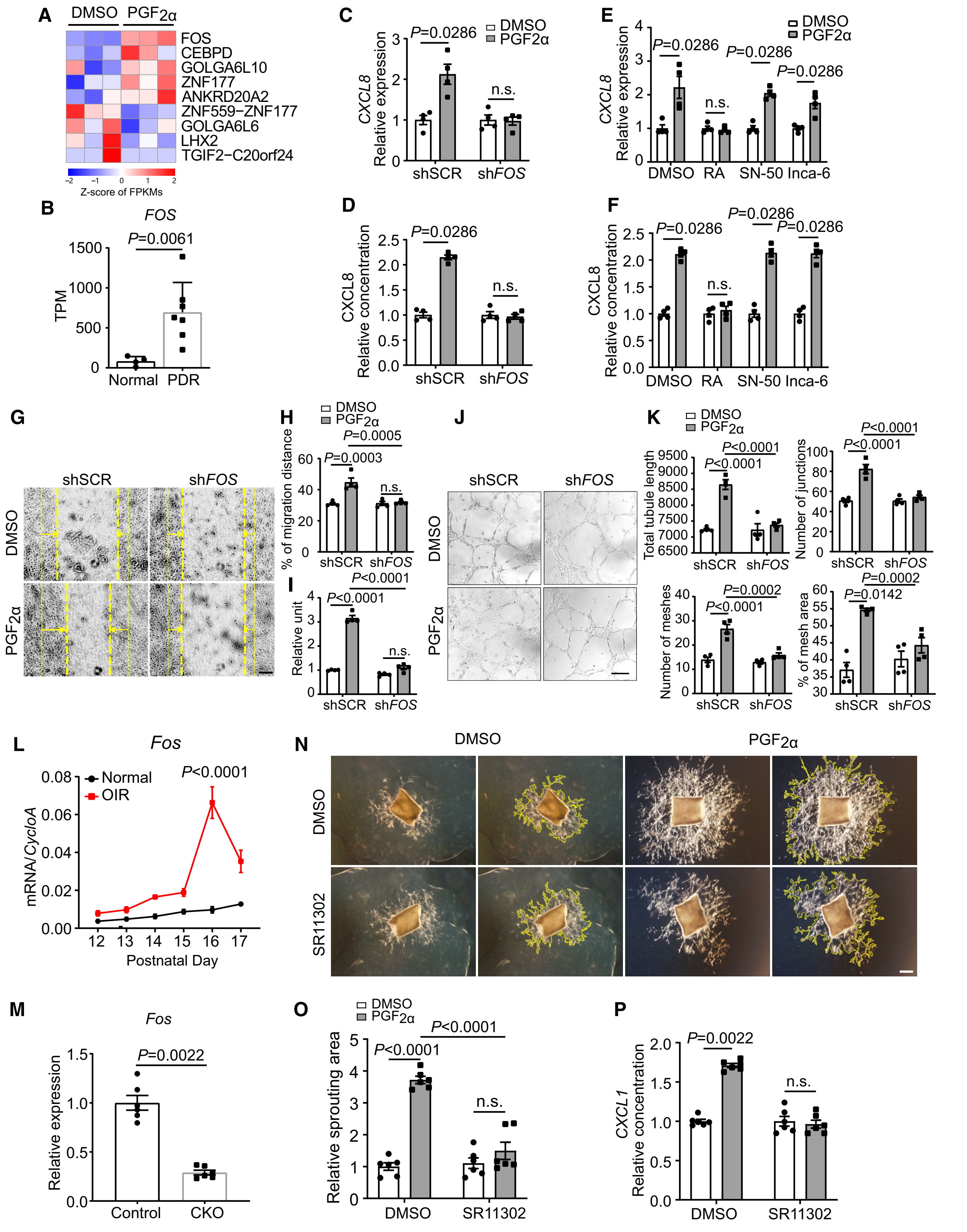

Figure 6.

 © 2022 The Authors

Figure 6. $PGF_{2\alpha}$ induces expression of ELR^+ CXC chemokines in HRMECs through transcription factor FOS.

A Heat map of differentially expressed transcription factors in HRMECs in response to 500 nM $PGF_{2\alpha}$ (*n* = 3).
B Relative expression levels of *FOS* in retinal microvascular ECs from patients with PDR (*n* = 4) compared with the corresponding expression levels from normal subjects (*n* = 7; Gene Expression Omnibus Database dataset GSE94019). TPM stands for "Transcripts per kilobase million."
C Effect of *FOS* knockdown on 500 nM $PGF_{2\alpha}$-induced *CXCL8* expression in HRMECs (*n* = 4).
D Effect of *FOS* knockdown on 500 nM $PGF_{2\alpha}$-induced HRMEC CXCL8 secretion in the culture medium (*n* = 4).
E, F Effect of RA (1 μM, AP-1 dimer inhibitor), SN-50 (100 μg/ml, NFκB inhibitor), and Inca-6 (2.5 μM, NFAT inhibitor) on 500 nM $PGF_{2\alpha}$-induced *CXCL8* expression in HRMECs and HRMEC CXCL8 secretion in the culture medium (*n* = 4).
G Representative images of scratch-induced migration of 500 nM $PGF_{2\alpha}$-treated HRMECs with or without *FOS* knockdown. The yellow solid line indicates the original position of the scratch, the dashed line indicates the position of the cells after migration, and the arrows represent the migratory direction.
H Quantitation of the cell-migration distance in H (*n* = 4).
I Effect of *FOS* knockdown on 500 nM $PGF_{2\alpha}$-induced HRMEC proliferation (*n* = 4).
J Representative images of tube formation by 500 nM $PGF_{2\alpha}$-treated HRMECs with or without *FOS* knockdown.
K Quantitation of total tube length, number of junctions, number of meshes, and percentage of mesh area in J (*n* = 4).
L *Fos* mRNA levels in retinas from OIR mice during postnatal days 12–17 (*n* = 3–4).
M Effect of *PTGFR* deficiency in ECs on *Fos* expression in the retina from OIR mice on postnatal day 16 (*n* = 6).
N Effect of FOS inhibitor SR11302 (1 μM) on 500 nM $PGF_{2\alpha}$-promoted sprouting of mouse aortic rings.
O Quantitation of sprouting areas in N (*n* = 6).
P Effect of FOS inhibitor SR11302 (1 μM) on 500 nM $PGF_{2\alpha}$-promoted CXCL1 secretion in mouse aortic ring culture medium (*n* = 6).

Data information: n.s. stands for "not significant." Data were analyzed by Mann–Whitney test (B, C, D, E, F, M, P) or two-way ANOVA with Tukey's multiple comparisons test (H, I, K, L, O). Scale bar: 100 μm (G) and 200 μm (J, N). Data in (B) are expressed as the mean + SD. Data in C, D, E, F, H, I, K, L, M, O, P are represented as mean ± SEM.
Source data are available online for this figure.

mice, peaking on postnatal day 16 (~ 7-fold compared with those from normoxic mice) during the vascular proliferation stage (Figs 1E, 5C and 6L). *PTGFR* deletion in ECs reduced retinal *Fos* expression in OIR mice on postnatal day 16 (Fig 6M). Consistently, inhibition of FOS by SR11302 diminished the $PGF_{2\alpha}$-mediated sprouting of mouse aortic rings (Fig 6N and O) and attenuated $PGF_{2\alpha}$-stimulated CXCL1 secretion in the culture medium (Fig 6P).

$PGF_{2\alpha}$/PTGFR axis found to regulate *FOS* expression in HRMECs through G_q/CAMK2G/p38/ELK-1 signaling

The PTGFR receptor may couple with G_q to activate phospholipase C (PLC) and boost intracellular Ca^{2+} (Ricciotti & FitzGerald, 2011), with G_{12} to activate Rho/ROCK signaling (Pierce *et al*, 1999; Goupil *et al*, 2010), or may activate PI3K/AKT signaling (Zheng *et al*, 2011). We then sought to determine how the $PGF_{2\alpha}$/PTGFR axis modulates *FOS* expression in HRMECs using conventional pharmacological approaches (Fig 7A). Treatment with PLC inhibitor U73122 and selective Ca^{2+} chelator BAPTA, but not ROCK or PI3K inhibitor, attenuated $PGF_{2\alpha}$-induced *FOS* expression, as well as downstream CXCL8 expression and secretion by HRMECs (Figs 7B and EV3A and B), indicating that $PGF_{2\alpha}$ regulates *FOS* expression in a Ca^{2+}-dependent manner by coupling with G_q. Ca^{2+} influx activates both PKC and CAMK2 (De, 2011), with the latter directly phosphorylating and activating MAPK (Wang *et al*, 2018). We observed that CAMK2 inhibitor KN93 and MAPK p38 inhibitor SB203580, but not PKC inhibitor RO318220, ERK inhibitor PD98059, or JNK inhibitor SP600125 (Fig 7C and D), blunted $PGF_{2\alpha}$-induced *FOS* expression as well as CXCL8 expression/secretion in HRMECs (Fig EV3C–F). This implied that the $PGF_{2\alpha}$/PTGFR axis regulates *FOS* expression, probably via the G_q/CAMK2/p38 pathway. As anticipated, $PGF_{2\alpha}$ stimulation activated p38 by enhancing its phosphorylation in HRMECs;

Figure 7. $PGF_{2\alpha}$ upregulates *FOS* expression in HRMECs through the G_q/CAMK2G/p38/ELK-1 signaling pathway.

A Schematic diagram of the potential PTGFR-mediated signaling pathways. All inhibitors of targeted molecules are shown in red.
B Effect of U73122 (10 μM), BAPTA (50 μM), Y27632 (5 μM), and Ly294002 (15 μM) treatment on 500 nM $PGF_{2\alpha}$-induced *FOS* gene expression in HRMECs (*n* = 4).
C Effect of KN93 (10 μM) and RO-318220 (250 nM) treatment on 500 nM $PGF_{2\alpha}$-induced *FOS* expression in HRMECs (*n* = 4).
D Effect of SB203580 (2 μM), PD98059 (20 μM), and SP600125 (60 nM) treatment on 500 nM $PGF_{2\alpha}$-induced *FOS* gene expression in HRMECs (*n* = 4).
E Effect of U73122 (10 μM), BAPTA (50 μM), and KN93 (10 μM) treatment on 500 nM $PGF_{2\alpha}$-induced p38 phosphorylation in HRMECs.
F Quantitation of the ratio of p38 phosphorylation to total p38 protein in E (*n* = 3).
G Effect of U73122 (10 μM), BAPTA (50 μM), and SB203580 (2 μM) on the phosphorylation of CAMK2 isoforms in 500 nM $PGF_{2\alpha}$-treated HRMECs.
H Quantitation of the ratio of CAMK2G phosphorylation to total CAMK2G in G (*n* = 3).
I Quantitation of the ratio of phosphorylated CAMK2D to total CAMK2D in G (*n* = 3).
J Effect of *CAMK2G* or *CAMK2D* knockdown on 500 nM $PGF_{2\alpha}$-triggered p38 phosphorylation in HRMECs.
K Quantitation of the ratio of p38 phosphorylation to total p38 protein in J (*n* = 3).
L Effect of *CAMK2G* or *CAMK2D* knockdown on 500 nM $PGF_{2\alpha}$-induced *FOS* expression in HRMECs (*n* = 4).
M Effect of U73122 (10 μM), BAPTA (50 μM), KN93 (10 μM), and SB203580 (2 μM) on ELK-1 phosphorylation in 500 nM $PGF_{2\alpha}$-treated HRMECs.
N Quantitation of the ratio of phosphorylated ELK-1 to total ELK-1 in M (*n* = 3).
O Schematic diagram of the $PGF_{2\alpha}$/PTGFR axis-mediated angiogenic signaling in retinal ECs.

Data information: n.s. stands for "not significant." Data were analyzed by Mann–Whitney test (B, C, D, and L) or unpaired Student's *t*-test (F, H, I, K, and N). Data are represented as mean ± SEM.
Source data are available online for this figure.

© 2022 The Authors

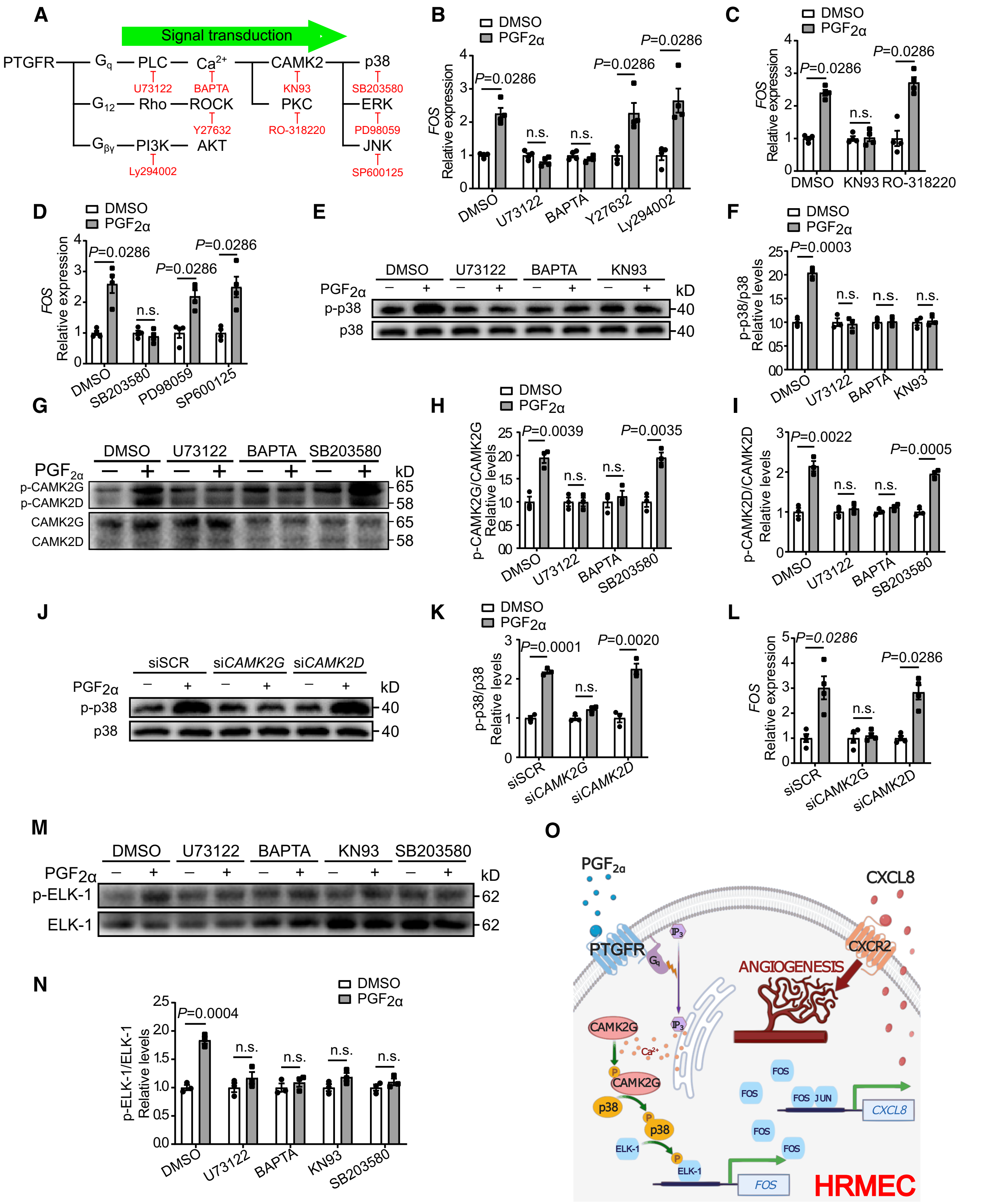

Figure 7.

this effect was attenuated by treatment with the PLC inhibitor U73122, selective Ca^{2+} chelator BAPTA, as well as CAMK2 inhibitor KN93 (Fig 7E and F). Two CAMK2 isoforms, CAMK2G and CAMK2D, are expressed in HRMECs (Ashraf *et al*, 2019), both of which are activated by $PGF_{2\alpha}$ treatment (Fig 7G–I). This effect was blocked by PLC inhibitor U73122 and Ca^{2+} chelator BAPTA, but not

 © 2022 The Authors

through inhibition of its downstream molecule p38 (Fig 7G–I). Interestingly, knockdown of *CAMK2G*, but not *CAMK2D* (Appendix Fig S6A and B), diminished $PGF_{2\alpha}$-induced p38 phosphorylation (Fig 7J and K), *FOS* upregulation (Fig 7L), as well as CXCL8 expression and secretion by HRMECs (Appendix Fig S6C and D), probably due to CAMK2G being the abundant isoform in retinal ECs (Fig 7G). The ELK-1 transcription factor is an important component of the ternary complex that controls *FOS* expression by binding to the serum response element (Marais *et al*, 1993). Its transcriptional activity can be modulated through Ser383 phosphorylation mediated by MAPKs, including p38 (Whitmarsh *et al*, 1995; Kenzel *et al*, 2009). Therefore, we proposed that the $PGF_{2\alpha}$/PTGFR axis induces *FOS* expression via p38-mediated ELK-1 activation. Indeed, $PGF_{2\alpha}$ treatment enhanced p-ELK-1 levels in HRMECs, and this effect was diminished by p38, PLC, as well as CAMK2 inhibition (Fig 7M and N). Taken together, the $PGF_{2\alpha}$/PTGFR axis facilitated FOS-driven CXCL8 expression in HRMECs through the G_q/CAMK2G/p38/ELK-1 signaling pathway (Fig 7O).

PTGFR inhibitor suppressed pathological retinal neovascularization in OIR mice

To determine the potential therapeutic effect of targeting the $PGF_{2\alpha}$/PTGFR axis in proliferative retinopathy, AL8810, a selective inhibitor of PTGFR, was administered to OIR mice at the vessel proliferation stage (days 12–17), twice per day (Fig 8A). As shown in Fig 8B and C, AL8810 administration attenuated oxygen-induced retinal microvascular proliferation in OIR mice by 60% but had no significant effect in *Cxcr2*$^{-/-}$ mice. However, *CXCR2* deletion suppressed proliferative retinopathy in OIR mice. There was no significant difference in retinal vaso-obliteration between these groups, indicating that OIR was induced successfully (Fig 8D). As anticipated, AL8810 treatment effectively suppressed *Fos* and *Cxcl1* expression in the retinas from both WT and *Cxcr2*$^{-/-}$ mice on postnatal day 16 (Fig 8E and F). In addition, we did not observe developmental defects of the retinal blood vessels in *Cxcr2*$^{-/-}$ mice (Appendix Fig S7A and B), just like in EC-specific *Ptgfr*-deficient mice (Appendix Fig S1). To further confirm the therapeutic effect by targeting PTGFR, we also tested another structurally different PTGFR antagonist OBE022 in OIR mice and also observed marked reduction of oxygen-induced retinal neovascularization in OBE022-treated mice (Fig EV4A–F). We did not observe any detectable signs of toxicity in AL8810- and OBE022-treated mice, including body weight changes (Appendix Fig S8A and B).

Discussion

$PGF_{2\alpha}$ generation is increased in inflammatory diseases, including diabetes mellitus (Zhang *et al*, 2010). Herein, we observed that $PGF_{2\alpha}$ generation was markedly increased in PDR patients and the $PGF_{2\alpha}$/PTGFR axis was activated in retinal ECs from OIR mice. Furthermore, *PTGFR* deletion in ECs attenuated pathological neovascularization in OIR mice by suppressing EC proliferation. Activation of the $PGF_{2\alpha}$/PTGFR axis promoted the FOS-mediated expression of ELR^+ CXC chemokines and tube formation by HRMECs. Administration of PTGFR inhibitor AL8810 mitigated retinal angiogenesis in OIR mice in a CXCR2-dependent manner. Mechanistically, PTGFR regulated *FOS* expression in HRMECs through the G_q/CAMK2G/p38/ELK-1 pathway. An induction of FOS-mediated ELR^+ CXC chemokine expression was observed in retinal ECs from diabetic patients with proliferative retinopathy. Therefore, the $PGF_{2\alpha}$/PTGFR axis may represent a therapeutic target for pathological retinal angiogenesis.

$PGF_{2\alpha}$ is a bioactive lipid mediator that regulates various physiological processes, such as blood pressure homeostasis (Yu *et al*, 2009) and female reproduction (Sugimoto *et al*, 2015). Its biosynthesis is markedly induced under oxidative stress, hypercholesterolemia, smoking, or inflammation, as observed during rheumatic disease (Basu *et al*, 2001) and type 1 or 2 diabetes mellitus (Helmersson *et al*, 2004; Basu *et al*, 2005). Activation of the $PGF_{2\alpha}$/PTGFR axis also impairs insulin sensitivity in diet-induced diabetic mice by promoting hepatic gluconeogenesis (Wang *et al*, 2018). Interestingly, we observed that circulating $PGF_{2\alpha}$ metabolites were even higher in diabetic patients with PDR than in those without DR or with NPDR. Likewise, $PGF_{2\alpha}$ generation and $PGF_{2\alpha}$ synthase expression were increased in retinas from OIR mice. We also detected a significant induction of *Ptgfr* expression in retinal tissue and ECs isolated from OIR mice during the vessel proliferation stage, peaking on postnatal day 16. Moreover, ablation of the PTGFR receptor in ECs alleviated oxygen-induced retinopathy in mice. Therefore, the activated $PGF_{2\alpha}$/PTGFR axis in ECs may directly contribute to the pathogenesis of proliferative retinopathy. Since diabetic nephropathy is also a microvascular complication of diabetes as retinopathy (Beckman & Creager, 2016) and excessive angiogenesis also occurs in diabetic nephropathy (Nakagawa *et al*, 2009), it is possible that the $PGF_{2\alpha}$/PTGFR axis may be involved in the pathogenesis of diabetic retinopathy. In agreement with our observations, the $PGF_{2\alpha}$/PTGFR axis exerted angiogenic effects in ectopic stromal cells during peritoneal endometriosis (Rakhila *et al*, 2016).

Figure 8. Administration of PTGFR inhibitor AL8810 attenuates retinal angiogenesis in OIR mice.

A Schematic diagram of AL8810 administration in OIR mice. i.p. stands for "intraperitoneally injected".
B Representative images of OIR retinas in WT and *Cxcr2*$^{-/-}$ mice with or without AL8810 treatment. The green color shows the isolectin B_4-stained vessels, the second row panels display the enlarged images of white boxes in the first row panels, the third row images show neovascular tufts (NV, white), and the fourth row images show the vaso-obliteration (VO, white) area.
C Quantitation of oxygen-induced retinal neovascularization in WT and *Cxcr2*$^{-/-}$ mice with or without AL8810 treatment ($n = 12$).
D Quantitation of retinal vaso-obliteration in WT and *Cxcr2*$^{-/-}$ mice with or without AL8810 treatment ($n = 12$).
E Effect of AL8810 on retinal *Fos* expression in WT and *Cxcr2*$^{-/-}$ OIR mice on postnatal day 16 ($n = 6$).
F Effect of AL8810 on retinal *Cxcl1* expression in WT and *Cxcr2*$^{-/-}$ OIR mice on postnatal day 16 ($n = 6$).

Data information: n.s. stands for "not significant." Data were analyzed by two-way ANOVA with Tukey's multiple comparisons test (C, D) or Mann–Whitney test (E, F). Scale bar: 500 μm (unmagnified image), 150 μm (magnified image). Data are represented as mean ± SEM.
Source data are available online for this figure.

© 2022 The Authors

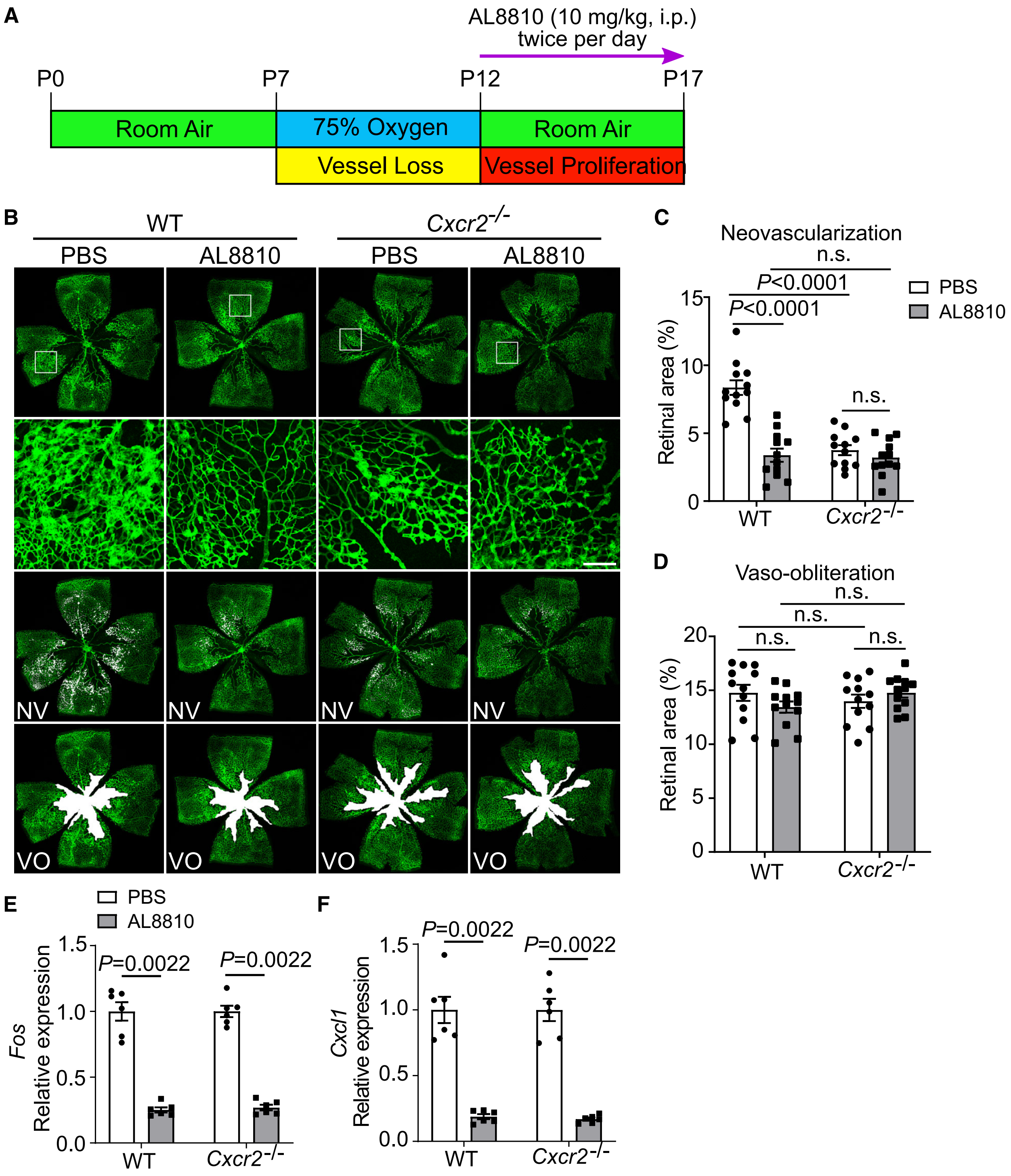

Figure 8.

Therefore, it may be worthwhile to monitor $PGF_{2\alpha}$ generation in vitreous humor from diabetic patients with PDR.

COX-2 expression is induced in retinal vessels and astrocytes in OIR mice; furthermore, a specific COX-2, but not COX-1, inhibitor attenuates OIR-induced retinal angiogenesis in mice (Sennlaub *et al*, 2003; Wilkinson-Berka *et al*, 2003). Interestingly, PGE_2 receptor EP2 agonist butaprost, and EP3 agonist M&B27767 partially rescue the inhibitory effects of COX-2 inhibitors on retinal neovascularization (Sennlaub *et al*, 2003), and EP4 inhibitor L-161982 reduces pathological neovascularization in OIR mice (Yanni *et al*, 2009), suggesting that COX-2-derived PGE_2 may promote pathological neovascularization through EP2-4 receptors. We found that genetic deficiency in ECs or pharmacological inhibition (AL8810 and OBE022) of PTGFR attenuates pathological neovascularization in

 © 2022 The Authors

OIR mice. These observations suggest that COX-2-drived $PGF_{2\alpha}$ may contribute to hypoxia-induced neovascularization in OIR mice. Circulating $PGF_{2\alpha}$ production is dramatically increased in patients with PDR compared with the production level in those with NPDR. It is possible that the intrinsic microenvironment to activate the $PGF_{2\alpha}$/PTGFR axis in PDR differs from that in OIR mice. ELR^+ CXC chemokines with a glutamic acid-leucine-arginine (ELR) amino acid sequence before the first cysteine of the CXC motif include CXCL1-3, CXCL5-6, and CXCL8 in humans, all of which are encoded by genes located on chromosome 4q12-q13 and function by specifically interacting with CXCR2 and CXCR1 receptors (Keeley *et al*, 2008). ELR^+ CXC chemokines play an important role in the initiation of inflammation and protection against bacterial infection (Tsai *et al*, 2000; Gregson *et al*, 2013). Cumulative evidence shows that ELR^+ CXC chemokines promote angiogenesis by binding and activating CXCR receptors in tumors (Strieter, 2005), and this angiogenic property is dependent on the tripeptide ELR motif (Keeley *et al*, 2008). ELR^+ CXC chemokines, including CXCL8 and CXCL2, are elevated in the vitreous body of diabetic patients with proliferative retinopathy (Lange *et al*, 2011; Koskela *et al*, 2013; Ghodasra *et al*, 2016). Bioinformatics analyses of a Gene Expression Omnibus Database data set (GSE94019) (Lam *et al*, 2017) revealed that the predominant ELR^+ CXC chemokines (*CXCL8* and *CXCL2*) expressed in retinal ECs were greatly upregulated in PDR patients (Fig 4D and E). $PGF_{2\alpha}$ treatment induced their expression in cultured HRMECs. In OIR mice, *CXCL8* functional homolog gene *Cxcl1* and *Cxcl2* were also markedly induced in retinas along with *Ptgfr* in mice on postnatal day 16. *PTGFR* deletion in retinal ECs reduced the expression of ELR^+ CXC chemokines in the retinas from OIR mice and diminished oxygen-triggered retinal neovascularization in mice. Additionally, *Cxcl2* and *Cxcl3* expression levels were highly upregulated in mouse retinas on postnatal day 14 following oxygen treatment, indicating the involvement of additional regulatory factors in the modulation of ELR^+ CXC chemokine expression in the OIR model. CXCR2 was the dominant ELR^+ CXC chemokine receptor expressed in retinal ECs, and *CXCR2* deficiency abolished the therapeutic effect of PTGFR inhibition on retinopathy in OIR mice. Indeed, the activation of CXCR2 signaling promotes angiogenesis in cancer (Dufies *et al*, 2019; Acker *et al*, 2020), as well as in chronic ischemic and inflammatory diseases (Bertini *et al*, 2012). Therefore, the $PGF_{2\alpha}$-mediated expression of ELR^+ CXC chemokines in ECs contributes to the pathogenesis of diabetic proliferative retinopathy via CXCR2. Notably, ELR^+ CXC chemokine-mediated infiltration of inflammatory cells, such as neutrophils (Barliya *et al*, 2017) and macrophages (Chaurasia *et al*, 2018), may also be involved in the development of diabetic proliferative retinopathy.

FOS, a proto-oncogene, binds JUN to form a heterodimer, resulting in the formation of transcriptional complex AP-1. The AP-1 complex controls cell differentiation and transformation, also regulating inflammatory processes, including the secretion of cytokines and chemokines (Schonthaler *et al*, 2011). Furthermore, AP-1 promotes the progression of inflammatory conditions, such as skin inflammation (Uluçkan *et al*, 2015) and rheumatoid arthritis (Shiozawa & Tsumiyama, 2009). We found that *FOS* expression is upregulated in the retinal ECs of PDR patients, reaching a peak on the same day as *Ptgfr* and *Cxcl1* in the retina from OIR mice during the angiogenesis stage (postnatal day 16). EC-specific genetic disruption of PTGFR suppressed oxygen-induced *Fos* expression in the retina. AP-1 inhibitor treatment or shRNA-mediated *FOS* silencing attenuated the $PGF_{2\alpha}$-induced upregulation of CXCL8 and CXCL2 in HRMECs, in turn suppressing proliferation and tube formation. These results indicate that increased FOS activity facilitates the $PGF_{2\alpha}$/PTGFR axis-driven HRMEC proliferation by upregulating the expression and secretion of the predominant ELR^+ CXC chemokines (CXCL8 and CXCL2). We did not detect any notable alterations in JUN expression in $PGF_{2\alpha}$-treated HRMECs. The *Vldlr*-deficient mouse is an established pathological retinal angiogenesis model, characterized by retinal neovascularization with invasion of the photoreceptor layer. Similarly, photoreceptor FOS-mediated inflammatory signals control blood vessel growth into the avascular photoreceptor layer in $Vldlr^{-/-}$ mice (Sun *et al*, 2017). Therefore, targeting FOS or AP-1 may prevent the progression of retinal proliferative vascular diseases.

The PTGFR couples G_q to activate Ca^{2+}-dependent signaling pathways (Zhang *et al*, 2010). We previously reported that PTGFR activates Ca^{2+}-dependent CAMK2G in hepatocytes and promotes fasting-induced hepatic gluconeogenesis by phosphorylating p38 (Wang *et al*, 2018). Both CAMK2G and CAMK2D isoforms are expressed in vascular endothelial cells (Cai *et al*, 2008). Using multiple screening approaches, we identified that CAMK2G mediated the $PGF_{2\alpha}$/PTGFR axis-triggered upregulation of *FOS* and *CXCL8* in HRMECs. Indeed, CAMK2 mediates the angiogenic activity of a range of growth factors in culture (Banumathi *et al*, 2011; Ashraf *et al*, 2019). Pharmacological blockade or genetic deletion of the CAMK2G or D isoforms suppresses pathological angiogenesis in ischemic retinas in mice (Ashraf *et al*, 2019). Interestingly, CAMK2 isoforms regulated angiogenic effects in response to different growth factors to varying extents (Ashraf *et al*, 2019). For instance, CAMK2G facilitates HGF- and IGF-1-induced tube formation but not that mediated via VEGF and bFGF. In contrast, CAMK2D mediates VEGF- and bFGF-induced HRMEC tube formation. Consistently, the $PGF_{2\alpha}$/PTGFR axis promoted *FOS* expression in HRMECs through the CAMK2G/p38 pathway without influencing *VEGF* expression. In bovine endometrial epithelial cells, however, PTGFR activation augments VEGF expression (Gao *et al*, 2018).

In summary, our study demonstrated that activation of the $PGF_{2\alpha}$/PTGFR axis accelerates experimental proliferative retinopathy through the secretion of ELR^+ CXC chemokines via FOS-mediated transcriptional regulation. Targeting the PTGFR and its downstream pathway may therefore serve as a novel approach for proliferative retinal disease management, independent of VEGF.

Materials and Methods

Patient serum sample collection

Patients with type 2 diabetes mellitus (T2DM) were recruited, from 2011 to 2014, at the Metabolic Disease Hospital of Tianjin Medical University, Tianjin Medical University General Hospital, the Tianjin People's Hospital, and the Eye Hospital of Tianjin Medical University (Wei *et al*, 2016), and their retinal complications were independently diagnosed by two experienced ophthalmologists. T2DM was defined as fasting plasma glucose $\geq$ 7.0 mmol/L, and/or 2-h oral glucose tolerance tests (OGTT) $\geq$ 11.1 mmol/L, or the use of anti-diabetic medicine. Serum samples were collected for medical tests

and written consent was obtained from all patients. The procedure was approved by the Human Ethics Committee of Tianjin Medical University (TMUhMEC2020034) in accordance with the Helsinki declaration and the principles set out in the Department of Health and Human Services Belmont Report. Serum levels of 15-keto-dihydro-$PGF_{2\alpha}$ and TXB_2 were determined using the 13, 14-dihydro-15-keto $PGF_{2\alpha}$ ELISA Kit (Cayman, 516671) and TXB_2 ELISA Kit (Cayman, 501020), respectively, according to the manufacturer's instructions.

Mice

All mice were of C57/BL6 background and were housed in an environment with controlled temperature (22°C ± 1°C) and relative humidity (50% ± 5%) on a 12-h:12-h light/dark cycle, with free access to sterile food and water. All animal care and experimental procedures were approved by the Institutional Animal Care and Use Committee of Tianjin Medical University (TMUaMEC 2020020) and complied with the National Institutes of Health (NIH) Guidelines for the care and use of laboratory animals. *Ptgfr*-floxed mice were crossed with Tie2-Cre mice (Kisanuki *et al*, 2001) or VE-Cadherin-Cre mice (Alva *et al*, 2006) to obtain endothelial cell-specific *Ptgfr* knockout mice. $Cxcr2^{-/-}$ mice [B6.129S2(C)-$Cxcr2^{tm1Mwm}$/J, Stock No: 006848] were obtained from Jackson Laboratory. For therapeutic experiments, mice were anesthetized with 5% isoflurane in an induction chamber and intraperitoneally injected with PTGFR receptor inhibitor AL8810 (10 mg/kg, Cayman, 16735) twice per day or administered orally with OBE022 (100 mg/kg, MCE, HY-112284) once per day for postnatal days 12–17. The administration method and dose of AL8810 and OBE022 are based on previous experimental methods on mouse models (Glushakov *et al*, 2013; Ahmad *et al*, 2015; Pohl *et al*, 2018), The animals were randomized into different treatments. The wild-type or knockout mice were randomly allocated to experimental groups.

Oxygen-induced retinopathy (OIR) in mice

Neonatal mice and their nursing mothers were exposed to 75% oxygen from postnatal day 7 to 12 and then bred in normal air until execution on postnatal day 17. Euthanasia was performed by exposure to excess CO_2 over 7 min. The retinas of the pups were isolated and stained with isolectin B_4 (Sigma, L2895) overnight. Four corners of the retinas were cut with spring scissors to flatten the whole-mount retinas, which were then photographed using a Leica TCS SP5 confocal microscope. The retinal neovascularization area was quantified using the SWIFT_NV (Stahl *et al*, 2009) plugin in ImageJ.

Laser capture microdissection (LCM)

The eyeballs of 16-day-old mice were removed and embedded in O.C.T. Compound (Sakura, 4583). Frozen sections (8 μm thick) were cut and placed on slides with PEN film (Carl Zeiss, 415190-9041-000), fixed sequentially in 70, 90, and 100% ethanol for 30 s, and washed with diethyl pyrocarbonate-treated water for 15 s. Sections were stained with isolectin B_4 (Sigma, L2895) at 25°C for 3 min in RNase inhibitor solution (Roche, 3335399001). Different layers of retinal tissues were separated using PALM MicroBeam (Carl Zeiss) and then collected directly in a collection tube containing RNeasy Micro Kit lysate (QIAGEN, 74004).

Prostanoid quantitation in retinas

Retinas were harvested and rinsed in ice-cold PBS containing indomethacin (5.6 μg/ml, Cayman, 70270) and snap-frozen in liquid nitrogen. Quick-frozen samples were lysed using a lysis solution (50 mM Tris–HCl, pH 7.5, 150 mM NaCl, 4 mM $CaCl_2$, 1.5% Triton X-100, protease inhibitors, and micrococcal nuclease) and processed using a tissue homogenizer. $PGF_{2\alpha}$ levels in the supernatant were measured using a $PGF_{2\alpha}$ high-sensitivity ELISA kit (ENZO, ADI-930-069). For the determination of $PGF_{2\alpha}$ and PGE_2 by liquid chromatography–tandem mass spectrometry (LCMS), UPLC BEHC18 column (1.7 μm, 100 × 2.1 mm i.d.) consisting of ethylene-bridged hybrid particles (Waters) was used for chromatographic separations. The isolates were analyzed using a 5500 QTRAP hybrid triple quadrupole linear ion trap mass spectrometer (AB Sciex) equipped with a turbo ion spray electrospray ionization source, as previously described (Zhu *et al*, 2019). $PGF_{2\alpha}$ and PGE_2 levels were normalized to the total protein concentration of the sample.

Intravitreal injection of lentivirus

Cxcl1 overexpression lentivirus was produced by Applied Biological Materials, and the target gene fragment was cloned into the pLenti-GIII-EF1a vector, with the empty vector used as a control. The viral titer was 4×10^9 TU/ml. Overexpression lentivirus (0.5 μl) was injected into the vitreous cavity of 5-day-old mice anesthetized with 5% isoflurane in an induction chamber, and an equal amount of control virus was injected into the contralateral vitreous cavity, followed by a regular OIR procedure. The injections reached the vitreous 1 mm posterior to the corneal limbus using a microinjector (Hamilton, 7632-01) fitted with a 33-gauge needle (Sun *et al*, 2015); the mice were treated with antibiotics to prevent infection. Those mice with eyeball infections after injection were excluded.

Cell culture

Human retinal microvascular endothelial cells (HRMECs, Cell Biologics, H-6065) were cultured in complete endothelial cell medium (ScienCell, SC-1001) under a humidified environment at 37°C and 5% CO_2.

RNA-seq analysis

HRMECs were cultured in endothelial cell medium (ScienCell, SC-1001) without the addition of endothelial cell-growth supplement (ECGS, ScienCell, 1052) and were treated with $PGF_{2\alpha}$ (500 nM, Cayman, 16010) or equivalent amounts of DMSO for 6 h. TRIzol reagent (Invitrogen, 15596018) was then added, and RNA was extracted according to the manufacturer's instructions. RNA-seq was performed using the BGISEQ-500 system. The results were compared to the NCBI GRCh38 reference genome using HISAT version 2.0.4 (Kim *et al*, 2015). Gene-expression levels were quantified using RSEM (Li & Dewey, 2011). Differentially expressed genes were detected using the DEGseq method (Wang *et al*, 2010). After correcting the *P* values of the assay results to Q (Storey & Tibshirani,

 © 2022 The Authors

2003), genes with a two-fold or greater differential expression and a Q value less than or equal to 0.001 were screened as differentially expressed genes. GO enrichment of genes upregulated in $PGF_{2\alpha}$-treated HRMECs was performed using Metascape (Zhou *et al*, 2019).

GEO data analysis

The Fastq file (SRP097696) was downloaded using the SRA Toolkit, version 2.9.2. The HISAT version 2.1.0 (Kim *et al*, 2015) was used for comparison to the reference genome UCSC hg38. Gene expression was calculated using featureCounts version 1.6.0 (Liao *et al*, 2014). Differential gene expression was calculated using the DESeq2 version 1.26.0 (Love *et al*, 2014). Two outlier samples (SRR5197978 and SRR5197982) were removed by principal component analysis prior to differential gene expression analysis, as described in a previous study (Gau *et al*, 2020).

Lentivirus-mediated *FOS* knockdown

The shRNA sequence targeting the human *FOS* gene (5′-GCGGAGACAGACCAACTAG'A-3′) and the scramble sequence (5′-TTCTCCGAACGTGTCAC'T-3′) used as a control were inserted into vector GV248. Cloning constructs and lentivirus packaging were performed by Genechem. The viral titer was 1×10^9 TU/ml. HRMECs were lentivirally transduced at 10 MOI using HitransG A (Genechem, REVG004) for 20 h, according to the manufacturer's instruction.

Cell-migration assay

HRMECs were spread into culture dishes at a concentration of 2×10^5 cells/ml, and the cells were treated with 10 μg/ml mitomycin C (Selleck, S8146) for 20 min at 37°C after the cells had grown to confluence. The cell monolayers were wounded by scraping. $PGF_{2\alpha}$ (500 nM, Cayman, 16010), SB265610 (1 μM, Sigma, SML0421), or equal volumes of DMSO were added to the endothelial cell medium, depending on the treatment group. Cells were then incubated at 37°C for an additional 15 h. Photographs of the cultured cells were taken after scratching and after cell migration using an IX71 microscope (Olympus). The distance between cells before and after migration was measured in each photograph.

Cell-proliferation assay

HRMECs were spread into 96-well plates at a concentration of 2×10^5 cells/ml, and $PGF_{2\alpha}$ [20–2,500 nM (Goupil *et al*, 2010), Cayman, 16010], SB265610 (1 μM, Sigma, SML0421), or equal volumes of DMSO were added to each well, depending on the treatment group. Cells were then incubated at 37°C for 24 h. Cell proliferation was assessed using the Cell Counting Kit-8 (Dojindo, CK04), according to the manufacturer's instructions.

Cell tube formation assay

HRMECs were spread at a density of 1×10^5 cells per well in 24-well plates coated with low growth factor Matrigel (Corning, 356230). $PGF_{2\alpha}$ (500 nM, Cayman, 16010), SB265610 (1 μM, Sigma, SML0421), or equal volumes of DMSO were added to each well, depending on the treatment group. Cells were then incubated at 37°C for 12 h. Cell tube formation in each well was photographed using an IX71 microscope (Olympus). The total tubule length, number of junctions, number of meshes, and percentage of mesh area were analyzed using the Angiogenesis Analyzer plugin in ImageJ.

Aortic ring sprouting assay

The 4-week-old mice were anesthetized with 5% isoflurane in an induction chamber and euthanized with cervical dislocation. Aortas were isolated from the mice, stripped of surrounding adipose tissue, cut into small rings, embedded in 30 μl of low growth factor Matrigel (Corning, 356230), and placed in 24-well plates. $PGF_{2\alpha}$ (500 nM, Cayman, 16010), SB265610 (1 μM, Sigma, SML0421), SR11302 (1 μM, APEXBIO, A8185), or equal volumes of DMSO were added to the endothelial cell medium, depending on the treatment group. Aortic rings were cultured at 37°C for 5 days, and the medium was changed every 2 days. Aortic rings were photographed after 5 days using an IX71 microscope (Olympus). The sprouting area was quantified using ImageJ software.

Drug treatment of cells

HRMECs were treated with $PGF_{2\alpha}$ (500 nM, Cayman, 16010) or an equivalent volume of DMSO and were collected 30 min later for protein extraction before western blot, 6 h later for RNA extraction, or 12 h later to detect chemokines secreted in the cell-culture medium using ELISA. For signaling pathway analysis, cells were pretreated with different inhibitors or an equivalent volume of DMSO for 30 min prior to $PGF_{2\alpha}$ administration. The inhibitors and concentrations used in the experiments were as follows: retinoic acid (RA) (1 μM, MCE, HY-14649), SN-50 (100 μg/ml, MCE, HY-P0151), Inca-6 (2.5 μM, Abcam, ab145864), U73122 (10 μM, MCE, HY-13419), BAPTA (50 μM, MCE, HY-100545), Y27632 (5 μM, MCE, HY-10071), Ly294002 (15 μM, MCE, HY-10108), KN93 (10 μM, MCE, HY-15465), RO-318220 (250 nM, MCE, HY-13866), SB203580 (2 μM, MCE, HY-10256), PD98059 (20 μM, MCE, HY-12028), and SP600125 (60 nM, MCE, HY-12041).

siRNA transfection

Human CAMK2G (horizon, L-004536-00) and CAMK2D (horizon, L-004042-00) ON-TARGETplus SMARTpool siRNAs were used to knockdown gene expression in HRMECs. ON-TARGETplus siCONTROL non-targeting siRNA (horizon, D-001810-10) was used as a control siRNA. Transfections were performed using the RNAiMAX Transfection Reagent (Thermo Fisher Scientific, 13778100) according to the manufacturer's instructions.

RNA extraction and quantitative real-time PCR

RNA was extracted from retinal tissues and cells using TRIzol reagent (Invitrogen, 15596018). RNA extraction from retinal layers isolated by LCM was performed using the RNeasy Micro Kit (QIAGEN, 74004). The extracted RNA was reverse-transcribed to generate cDNA using an RT reagent Kit (Takara, RR047A). Quantitative analysis of gene expression was performed on a LightCycler 480

© 2022 The Authors

Instrument II (Roche) with TB Green Premix Ex Taq (Takara, RR420A) and specific primers. RNA expression was normalized to *CypA* for mouse retinal tissue or *ACTB* for HRMECs. The primer sequences used are shown in Appendix Table S2.

Western blots

Cells or tissues were lysed using RIPA buffer (Cell Signaling Technology, 9806). Protease Inhibitor Cocktail (Cell Signaling Technology, 5871) was added to the lysates. Proteins were separated by SDS–PAGE and transferred to nitrocellulose membranes to assess abundance by primary antibody detection. The primary antibodies used were Phospho-CaMKII (Thr286) Rabbit mAb (1:1,000; Cell Signaling Technology, cat# 12716), CaMKII (pan) Rabbit mAb (1:1,000; Cell Signaling Technology, cat# 4436), Phospho-Elk-1 (Ser383) Antibody (1:1,000; Cell Signaling Technology, 9181), Elk-1 Antibody (1:1000; Cell Signaling Technology, cat# 9182), p38 MAPK Antibody (1:1,000; Cell Signaling Technology, cat# 9212), Phospho-p38 MAPK (Thr180/Tyr182) Antibody (1:1,000; Cell Signaling Technology, cat# 9211), Anti-c-Fos (phosphor T232) antibody (1 μg/ml; Abcam, cat# ab43175) and Anti-c-Fos (phosphor T232) antibody (1 μg/ml; Abcam, cat# ab90289), Anti-GRO alpha antibody (4 μg/ml Abcam, cat# ab86436), and Monoclonal Anti-β-Actin (1 μg/ml; Sigma, cat# A1978). Signals were visualized using ECL Western Blotting Substrate (Thermo Fisher Scientific, 32106). Band intensities were quantified using the ImageJ software for grayscale statistics.

ELISA

The culture medium of HRMECs or mouse aortic rings was collected for chemokine level detection. The Human IL-8 ELISA Kit (Abcam, ab214030), Human CXCL2 ELISA Kit (Multisciences, EK1264), and Mouse CXCL1 ELISA Kit (BOSTER, EK0723) were used for chemokine detection, according to the manufacturer's instructions.

Retinal endothelial cell separation

Retinas were isolated from postnatal day 16 mice with the OIR model, then digested with 1 mg/ml Collagenase/Dispase (Roche, 10269638001) and 60 U/ml DNase I (Roche, 4716728001) in endothelial cell medium (ScienCell, SC-1001) at 37°C for 15 min. The single-cell suspension was strained through a 70 μm cell strainer and washed with PBS containing 2% FBS and 2 mM EDTA. Cells were then incubated with CD45 MicroBeads (Miltenyi, 130-052-301) to exclude the $CD45^+$ cells. Thereafter, the cells were incubated with CD31 MicroBeads (Miltenyi, 130-097-418) to obtain the endothelial cells.

Statistical analysis

Statistical analyses were performed using GraphPad Prism version 8. All data are expressed as the mean ± SEM, unless stated otherwise. All data sets were tested for normality of distribution using the Shapiro–Wilk test. Comparisons between two groups were analyzed using Mann–Whitney test or unpaired Student's *t*-test. Multiple groups were compared by two-way ANOVA with Tukey's post-hoc test or Kruskal–Wallis test with Dunn's post hoc test. The experimenters were blinded to animal genotype and grouping information and all data were derived from biological replicates as indicated. *P*-values less than 0.05 were considered statistically significant.

The paper explained

Problem

The pathological retinal angiogenesis, characterized by an aberrantly proliferating vascular tuft structure, is the hallmark of retinopathy of prematurity and proliferative diabetic retinopathy (PDR). Current anti-angiogenic therapy for proliferative retinopathy targets the vascular endothelial growth factor, but many patients do not radically benefit from this therapy. Therefore, there is an urgent need to identify novel angiogenic factors implicated in proliferative retinopathy and develop respective therapeutics.

Results

Here, we observed $PGF_{2\alpha}$ generation is markedly increased in PDR patients as well as in an oxygen-induced retinopathy (OIR) mouse model. The $PGF_{2\alpha}$ receptor (PTGFR) is induced in retinal endothelial cells (ECs) during the vessel proliferative stage in OIR mice. The $PGF_{2\alpha}$/PTGFR axis promotes retinal EC proliferation through CAMK2G/p38/ELK-1/FOS pathway-mediated ELR^+ CXC chemokine secretion. Treatment with PTGFR inhibitor AL8810 attenuates retinal neovascularization in OIR mice.

Impact

These results demonstrate that $PGF_{2\alpha}$ is a proliferative lipid mediator of pathologic retinal neovascularization, and blockade of PTGFR receptor may represent a new avenue for the treatment of retinal neovascularization, particularly in PDR.

Data availability

Data (RNA sequencing) of HRMECs treated with $PGF_{2\alpha}$ (500 nM, Cayman, 16010) or equivalent amounts of DMSO for 6 h in this study has been deposited in the GEO database (https://www.ncbi.nlm.nih.gov/geo) with the accession number GSE164199 (http://www.ncbi.nlm.nih.gov/geo/query/acc.cgi?acc=GSE164199), and all datasets were made freely available upon acceptance, without restriction.

Expanded View for this article is available online.

Acknowledgements

The authors thank Prof. Dr. Andreas Stahl for providing SWIFT_NV macros and BioRender.com for schematic illustration created. The present study was supported by the National Natural Science Foundation of China (grants 81790623 and 82030015), Ministry of Science and Technology of the people's republic of China (2021YFC2701104). Dr. Yu is a fellow at the Jiangsu Collaborative Innovation Center for Cardiovascular Disease Translational Medicine.

Author contributions

Ying Yu: Conceptualization; resources; data curation; supervision; funding acquisition; writing – original draft; project administration; writing – review and editing. **Yan Zhao:** Conceptualization; data curation; formal analysis; validation; investigation; visualization; methodology; writing – original draft; project administration. **Yi Lei:** Data curation; formal analysis; validation; investigation; visualization; methodology; writing – review and editing.

© 2022 The Authors

Huying Ning: Data curation; formal analysis; investigation; visualization; methodology. **Yaqiang Zhang:** Investigation; methodology. **Guilin Chen:** Methodology. **Chenchen Wang:** Methodology. **Qiangyou Wan:** Methodology. **Shumin Guo:** Methodology. **Qian Liu:** Methodology. **Ruotian Xie:** Methodology. **Yujuan Zhuo:** Methodology. **Shuai Yan:** Methodology. **Jing Zhao:** Resources. **Fengjiang Wei:** Resources. **Lu Wang:** Methodology. **Xiaohong Wang:** Supervision; writing – review and editing. **Weidong Li:** Resources; supervision. **Hua Yan:** Supervision; writing – review and editing.

Disclosure and competing interests statement
The authors declare that they have no conflict of interest.

References

Acker G, Zollfrank J, Jelgersma C, Nieminen-Kelhä M, Kremenetskaia I, Mueller S, Ghori A, Vajkoczy P, Brandenburg S (2020) The CXCR2/CXCL2 signalling pathway – an alternative therapeutic approach in high-grade glioma. *Eur J Cancer* 126: 106 – 115

Ahmad SF, Akoum A, Horne AW (2015) Selective modulation of the prostaglandin F2α pathway markedly impacts on endometriosis progression in a xenograft mouse model. *Mol Hum Reprod* 21: 905 – 916

Alva JA, Zovein AC, Monvoisin A, Murphy T, Salazar A, Harvey NL, Carmeliet P, Iruela-Arispe ML (2006) VE-cadherin-Cre-recombinase transgenic mouse: a tool for lineage analysis and gene deletion in endothelial cells. *Dev Dyn* 235: 759 – 767

Ashraf S, Bell S, O'Leary C, Canning P, Micu I, Fernandez JA, O'Hare M, Barabas P, McCauley H, Brazil DP *et al* (2019) CAMKII as a therapeutic target for growth factor-induced retinal and choroidal neovascularization. *JCI Insight* 4: e122442

Banumathi E, O'Connor A, Gurunathan S, Simpson DA, McGeown JG, Curtis TM (2011) VEGF-induced retinal angiogenic signaling is critically dependent on Ca^{2+} signaling by Ca^{2+}/calmodulin-dependent protein kinase II. *Invest Ophthalmol Vis Sci* 52: 3103 – 3111

Barliya T, Dardik R, Nisgav Y, Dachbash M, Gaton D, Kenet G, Ehrlich R, Weinberger D, Livnat T (2017) Possible involvement of NETosis in inflammatory processes in the eye: evidence from a small cohort of patients. *Mol Vis* 23: 922 – 932

Basu S, Whiteman M, Mattey DL, Halliwell B (2001) Raised levels of F(2)-isoprostanes and prostaglandin F(2alpha) in different rheumatic diseases. *Ann Rheum Dis* 60: 627 – 631

Basu S, Larsson A, Vessby J, Vessby B, Berne C (2005) Type 1 diabetes is associated with increased cyclooxygenase- and cytokine-mediated inflammation. *Diabetes Care* 28: 1371 – 1375

Beckman JA, Creager MA (2016) Vascular complications of diabetes. *Circ Res* 118: 1771 – 1785

Bertini R, Barcelos LS, Beccari AR, Cavalieri B, Moriconi A, Bizzarri C, Di Benedetto P, Di Giacinto C, Gloaguen I, Galliera E *et al* (2012) Receptor binding mode and pharmacological characterization of a potent and selective dual CXCR1/CXCR2 non-competitive allosteric inhibitor. *Br J Pharmacol* 165: 436 – 454

Bobrovnikova-Marjon EV, Marjon PL, Barbash O, Vander Jagt DL, Abcouwer SF (2004) Expression of angiogenic factors vascular endothelial growth factor and interleukin-8/CXCL8 is highly responsive to ambient glutamine availability: Role of nuclear factor-kappaB and activating protein-1. *Cancer Res* 64: 4858 – 4869

Bresson E, Lacroix-Pepin N, Boucher-Kovalik S, Chapdelaine P, Fortier MA (2012) The prostaglandin F synthase activity of the human aldose reductase AKR1B1 brings new lenses to look at pathologic conditions. *Front Pharmacol* 3: 98

Cai H, Liu D, Garcia JG (2008) CaM kinase II-dependent pathophysiological signalling in endothelial cells. *Cardiovasc Res* 77: 30 – 34

Chaurasia SS, Lim RR, Parikh BH, Wey YS, Tun BB, Wong TY, Luu CD, Agrawal R, Ghosh A, Mortellaro A *et al* (2018) The NLRP3 inflammasome may contribute to pathologic neovascularization in the advanced stages of diabetic retinopathy. *Sci Rep* 8: 2847

Connor KM, Krah NM, Dennison RJ, Aderman CM, Chen J, Guerin KI, Sapieha P, Stahl A, Willett KL, Smith LE (2009) Quantification of oxygen-induced retinopathy in the mouse: a model of vessel loss, vessel regrowth and pathological angiogenesis. *Nat Protoc* 4: 1565 – 1573

De A (2011) Wnt/Ca^{2+} signaling pathway: a brief overview. *Acta Biochim Biophys Sin (Shanghai)* 43: 745 – 756

Demaine AG (2003) Polymorphisms of the aldose reductase gene and susceptibility to diabetic microvascular complications. *Curr Med Chem* 10: 1389 – 1398

Dufies M, Grytsai O, Ronco C, Camara O, Ambrosetti D, Hagege A, Parola J, Mateo L, Ayrault M, Giuliano S *et al* (2019) New CXCR1/CXCR2 inhibitors represent an effective treatment for kidney or head and neck cancers sensitive or refractory to reference treatments. *Theranostics* 9: 5332 – 5346

Gao L, Gao R, Mao W, Liu B, Zhang S, Tahala D, Fu C, Shen Y, Wu J, Deng Y *et al* (2018) PTGFR activation promotes the expression of PTGS-2 and growth factors via activation of the PKC signaling pathway in bovine endometrial epithelial cells. *Anim Reprod Sci* 199: 30 – 39

Gau D, Vignaud L, Allen A, Guo Z, Sahel J, Boone D, Koes D, Guillonneau X, Roy P (2020) Disruption of profilin1 function suppresses developmental and pathological retinal neovascularization. *J Biol Chem* 295: 9618 – 9629

Ghodasra DH, Fante R, Gardner TW, Langue M, Niziol LM, Besirli C, Cohen SR, Dedania VS, Demirci H, Jain N *et al* (2016) Safety and feasibility of quantitative multiplexed cytokine analysis from office-based vitreous aspiration. *Invest Ophthalmol Vis Sci* 57: 3017 – 3023

Glushakov AV, Robbins SW, Bracy CL, Narumiya S, Doré S (2013) Prostaglandin F2α FP receptor antagonist improves outcomes after experimental traumatic brain injury. *J Neuroinflammation* 10: 132

Good RI, McGarrity A, Sheehan R, James TE, Miller H, Stephens J, Watkins S, McConnachie A, Goodall AH, Oldroyd KG (2015) Variation in thromboxane B2 concentrations in serum and plasma in patients taking regular aspirin before and after clopidogrel therapy. *Platelets* 26: 17 – 24

Goupil E, Tassy D, Bourguet C, Quiniou C, Wisehart V, Petrin D, Le Gouill C, Devost D, Zingg HH, Bouvier M *et al* (2010) A novel biased allosteric compound inhibitor of parturition selectively impedes the prostaglandin F2alpha-mediated rho/ROCK signaling pathway. *J Biol Chem* 285: 25624 – 25636

Grbic DM, Degagne E, Larrivee JF, Bilodeau MS, Vinette V, Arguin G, Stankova J, Gendron FP (2012) P2Y6 receptor contributes to neutrophil recruitment to inflamed intestinal mucosa by increasing CXC chemokine ligand 8 expression in an AP-1-dependent manner in epithelial cells. *Inflamm Bowel Dis* 18: 1456 – 1469

Gregson AL, Wang X, Weigt SS, Palchevskiy V, Lynch JP 3rd, Ross DJ, Kubak BM, Saggar R, Fishbein MC, Ardehali A *et al* (2013) Interaction between pseudomonas and CXC chemokines increases risk of bronchiolitis obliterans syndrome and death in lung transplantation. *Am J Respir Crit Care Med* 187: 518 – 526

Helmersson J, Vessby B, Larsson A, Basu S (2004) Association of type 2 diabetes with cyclooxygenase-mediated inflammation and oxidative stress in an elderly population. *Circulation* 109: 1729 – 1734

© 2022 The Authors

Hol J, Wilhelmsen L, Haraldsen G (2010) The murine IL-8 homologues KC, MIP-2, and LIX are found in endothelial cytoplasmic granules but not in Weibel-Palade bodies. *J Leukoc Biol* 87: 501 – 508

Keeley EC, Mehrad B, Strieter RM (2008) Chemokines as mediators of neovascularization. *Arterioscler Thromb Vasc Biol* 28: 1928 – 1936

Kenzel S, Santos-Sierra S, Deshmukh SD, Moeller I, Ergin B, Fitzgerald KA, Lien E, Akira S, Golenbock DT, Henneke P (2009) Role of p38 and early growth response factor 1 in the macrophage response to group B streptococcus. *Infect Immun* 77: 2474 – 2481

Kim D, Langmead B, Salzberg SL (2015) HISAT: a fast spliced aligner with low memory requirements. *Nat Methods* 12: 357 – 360

Kisanuki YY, Hammer RE, Miyazaki J, Williams SC, Richardson JA, Yanagisawa M (2001) Tie2-Cre transgenic mice: a new model for endothelial cell-lineage analysis *in vivo*. *Dev Biol* 230: 230 – 242

Klimko PG, Sharif NA (2019) Discovery, characterization and clinical utility of prostaglandin agonists for the treatment of glaucoma. *Br J Pharmacol* 176: 1051 – 1058

Koskela UE, Kuusisto SM, Nissinen AE, Savolainen MJ, Liinamaa MJ (2013) High vitreous concentration of IL-6 and IL-8, but not of adhesion molecules in relation to plasma concentrations in proliferative diabetic retinopathy. *Ophthalmic Res* 49: 108 – 114

Lam JD, Oh DJ, Wong LL, Amarnani D, Park-Windhol C, Sanchez AV, Cardona-Velez J, McGuone D, Stemmer-Rachamimov AO, Eliott D *et al* (2017) Identification of RUNX1 as a mediator of aberrant retinal angiogenesis. *Diabetes* 66: 1950 – 1956

Lange CAK, Stavrakas P, Luhmann UFO, de Silva DJ, Ali RR, Gregor ZJ, Bainbridge JWB (2011) Intraocular oxygen distribution in advanced proliferative diabetic retinopathy. *Am J Ophthalmol* 152: 406 – 412

Lee R, Wong TY, Sabanayagam C (2015) Epidemiology of diabetic retinopathy, diabetic macular edema and related vision loss. *Eye Vis (Lond)* 2: 17

Li B, Dewey CN (2011) RSEM: accurate transcript quantification from RNA-seq data with or without a reference genome. *BMC Bioinformatics* 12: 323

Li WJ, Lu JW, Zhang CY, Wang WS, Ying H, Myatt L, Sun K (2020) PGE2 vs PGF2α in human parturition. *Placenta* 104: 208 – 219

Liao Y, Smyth GK, Shi W (2014) featureCounts: an efficient general purpose program for assigning sequence reads to genomic features. *Bioinformatics* 30: 923 – 930

Love MI, Huber W, Anders S (2014) Moderated estimation of fold change and dispersion for RNA-seq data with DESeq2. *Genome Biol* 15: 550

Marais R, Wynne J, Treisman R (1993) The SRF accessory protein elk-1 contains a growth factor-regulated transcriptional activation domain. *Cell* 73: 381 – 393

Mintz-Hittner HA, Kennedy KA, Chuang AZ (2011) Efficacy of intravitreal bevacizumab for stage 3+ retinopathy of prematurity. *N Engl J Med* 364: 603 – 615

Nakagawa T, Kosugi T, Haneda M, Rivard CJ, Long DA (2009) Abnormal angiogenesis in diabetic nephropathy. *Diabetes* 58: 1471 – 1478

Penn JS, Madan A, Caldwell RB, Bartoli M, Caldwell RW, Hartnett ME (2008) Vascular endothelial growth factor in eye disease. *Prog Retin Eye Res* 27: 331 – 371

Pierce KL, Fujino H, Srinivasan D, Regan JW (1999) Activation of FP prostanoid receptor isoforms leads to rho-mediated changes in cell morphology and in the cell cytoskeleton. *J Biol Chem* 274: 35944 – 35949

Pohl O, Chollet A, Kim SH, Riaposova L, Spezia F, Gervais F, Guillaume P, Lluel P, Meen M, Lemaux F *et al* (2018) OBE022, an oral and selective prostaglandin F2alpha receptor antagonist as an effective and safe modality for the treatment of preterm labor. *J Pharmacol Exp Ther* 366: 349 – 364

Rakhila H, Al-Akoum M, Doillon C, Lacroix-Pepin N, Leboeuf M, Lemyre M, Akoum A, Pouliot M (2016) Augmented angiogenic factors expression via FP signaling pathways in peritoneal endometriosis. *J Clin Endocrinol Metab* 101: 4752 – 4763

Rezzola S, Loda A, Corsini M, Semeraro F, Annese T, Presta M, Ribatti D (2020) Angiogenesis-inflammation cross talk in diabetic retinopathy: novel insights from the chick embryo chorioallantoic membrane/human vitreous platform. *Front Immunol* 11: 581288

Ricciotti E, FitzGerald GA (2011) Prostaglandins and inflammation. *Arterioscler Thromb Vasc Biol* 31: 986 – 1000

Rodrigues EB, Farah ME, Maia M, Penha FM, Regatieri C, Melo GB, Pinheiro MM, Zanetti CR (2009) Therapeutic monoclonal antibodies in ophthalmology. *Prog Retin Eye Res* 28: 117 – 144

Schonthaler HB, Guinea-Viniegra J, Wagner EF (2011) Targeting inflammation by modulating the Jun/AP-1 pathway. *Ann Rheum Dis* 70: i109 – i112

Seery CW, Betesh S, Guo S, Zarbin MA, Bhagat N, Wagner RS (2020) Update on the use of anti-VEGF drugs in the treatment of retinopathy of prematurity. *J Pediatr Ophthalmol Strabismus* 57: 351 – 362

Sennlaub F, Valamanesh F, Vazquez-Tello A, El-Asrar AM, Checchin D, Brault S, Gobeil F, Beauchamp MH, Mwaikambo B, Courtois Y *et al* (2003) Cyclooxygenase-2 in human and experimental ischemic proliferative retinopathy. *Circulation* 108: 198 – 204

Shiozawa S, Tsumiyama K (2009) Pathogenesis of rheumatoid arthritis and c-Fos/AP-1. *Cell Cycle* 8: 1539 – 1543

Stahl A, Connor KM, Sapieha P, Willett KL, Krah NM, Dennison RJ, Chen J, Guerin KI, Smith LE (2009) Computer-aided quantification of retinal neovascularization. *Angiogenesis* 12: 297 – 301

Storey JD, Tibshirani R (2003) Statistical significance for genomewide studies. *Proc Natl Acad Sci U S A* 100: 9440 – 9445

Strieter RM (2005) Masters of angiogenesis. *Nat Med* 11: 925 – 927

Sugimoto Y, Inazumi T, Tsuchiya S (2015) Roles of prostaglandin receptors in female reproduction. *J Biochem* 157: 73 – 80

Sun Y, Liu CH, SanGiovanni JP, Evans LP, Tian KT, Zhang B, Stahl A, Pu WT, Kamenecka TM, Solt LA *et al* (2015) Nuclear receptor RORalpha regulates pathologic retinal angiogenesis by modulating SOCS3-dependent inflammation. *Proc Natl Acad Sci U S A* 112: 10401 – 10406

Sun Y, Lin Z, Liu CH, Gong Y, Liegl R, Fredrick TW, Meng SS, Burnim SB, Wang Z, Akula JD *et al* (2017) Inflammatory signals from photoreceptor modulate pathological retinal angiogenesis via c-Fos. *J Exp Med* 214: 1753 – 1767

Tsai WC, Strieter RM, Mehrad B, Newstead MW, Zeng X, Standiford TJ (2000) CXC chemokine receptor CXCR2 is essential for protective innate host response in murine *Pseudomonas aeruginosa* pneumonia. *Infect Immun* 68: 4289 – 4296

Uluçkan Ö, Guinea-Viniegra J, Jimenez M, Wagner EF (2015) Signalling in inflammatory skin disease by AP-1 (Fos/Jun). *Clin Exp Rheumatol* 33: S44 – S49

Wang L, Feng Z, Wang X, Wang X, Zhang X (2010) DEGseq: an R package for identifying differentially expressed genes from RNA-seq data. *Bioinformatics* 26: 136 – 138

Wang Y, Yan S, Xiao B, Zuo S, Zhang Q, Chen G, Yu Y, Chen D, Liu Q, Liu Y *et al* (2018) Prostaglandin F(2α) facilitates hepatic glucose production through CaMKIIγ/p38/FOXO1 signaling pathway in fasting and obesity. *Diabetes* 67: 1748 – 1760

Wang MH, Hsiao G, Al-Shabrawey M (2020) Eicosanoids and oxidative stress in diabetic retinopathy. *Antioxidants (Basel)* 9: 520

 © 2022 The Authors

Wei F, Chang B, Yang X, Wang Y, Chen L, Li WD (2016) Serum uric acid levels were dynamically coupled with hemoglobin A1c in the development of type 2 diabetes. *Sci Rep* 6: 28549

Wells JA, Glassman AR, Ayala AR, Jampol LM, Aiello LP, Antoszyk AN, Arnold-Bush B, Baker CW, Bressler NM, Browning DJ *et al* (2015) Aflibercept, bevacizumab, or ranibizumab for diabetic macular edema. *N Engl J Med* 372: 1193 – 1203

Whitmarsh AJ, Shore P, Sharrocks AD, Davis RJ (1995) Integration of MAP kinase signal transduction pathways at the serum response element. *Science* 269: 403 – 407

Wilkinson-Berka JL, Alousis NS, Kelly DJ, Gilbert RE (2003) COX-2 inhibition and retinal angiogenesis in a mouse model of retinopathy of prematurity. *Invest Ophthalmol Vis Sci* 44: 974 – 979

Yang S, Zhao J, Sun X (2016) Resistance to anti-VEGF therapy in neovascular age-related macular degeneration: a comprehensive review. *Drug Des Devel Ther* 10: 1857 – 1867

Yanni SE, Barnett JM, Clark ML, Penn JS (2009) The role of PGE2 receptor EP4 in pathologic ocular angiogenesis. *Invest Ophthalmol Vis Sci* 50: 5479 – 5486

Yanni SE, Clark ML, Yang R, Bingaman DP, Penn JS (2010) The effects of nepafenac and amfenac on retinal angiogenesis. *Brain Res Bull* 81: 310 – 319

Yu Y, Lucitt MB, Stubbe J, Cheng Y, Friis UG, Hansen PB, Jensen BL, Smyth EM, FitzGerald GA (2009) Prostaglandin F2alpha elevates blood pressure and promotes atherosclerosis. *Proc Natl Acad Sci U S A* 106: 7985 – 7990

Zhang J, Gong Y, Yu Y (2010) PG F(2α) receptor: a promising therapeutic target for cardiovascular disease. *Front Pharmacol* 1: 116

Zheng J, Feng X, Hou L, Cui Y, Zhu L, Ma J, Xia Z, Zhou W, Chen H (2011) Latanoprost promotes neurite outgrowth in differentiated RGC-5 cells via the PI3K-Akt-mTOR signaling pathway. *Cell Mol Neurobiol* 31: 597 – 604

Zhou Y, Zhou B, Pache L, Chang M, Khodabakhshi AH, Tanaseichuk O, Benner C, Chanda SK (2019) Metascape provides a biologist-oriented resource for the analysis of systems-level datasets. *Nat Commun* 10: 1523

Zhu L, Xu C, Huo X, Hao H, Wan Q, Chen H, Zhang X, Breyer RM, Huang Y, Cao X *et al* (2019) The cyclooxygenase-1/mPGES-1/endothelial prostaglandin EP4 receptor pathway constrains myocardial ischemia-reperfusion injury. *Nat Commun* 10: 1888

License: This is an open access article under the terms of the Creative Commons Attribution License, which permits use, distribution and reproduction in any medium, provided the original work is properly cited.
