# Supplementary figures and images for: PGF2α facilitates pathological retinal angiogenesis by modulating endothelial FOS‐driven ELR + CXC chemokine expression
Source: EMBO Mol Med. 2022 Dec 13;15(1):e16373. doi: 10.15252/emmm.202216373 (PMC9832840; doi:10.15252/emmm.202216373)

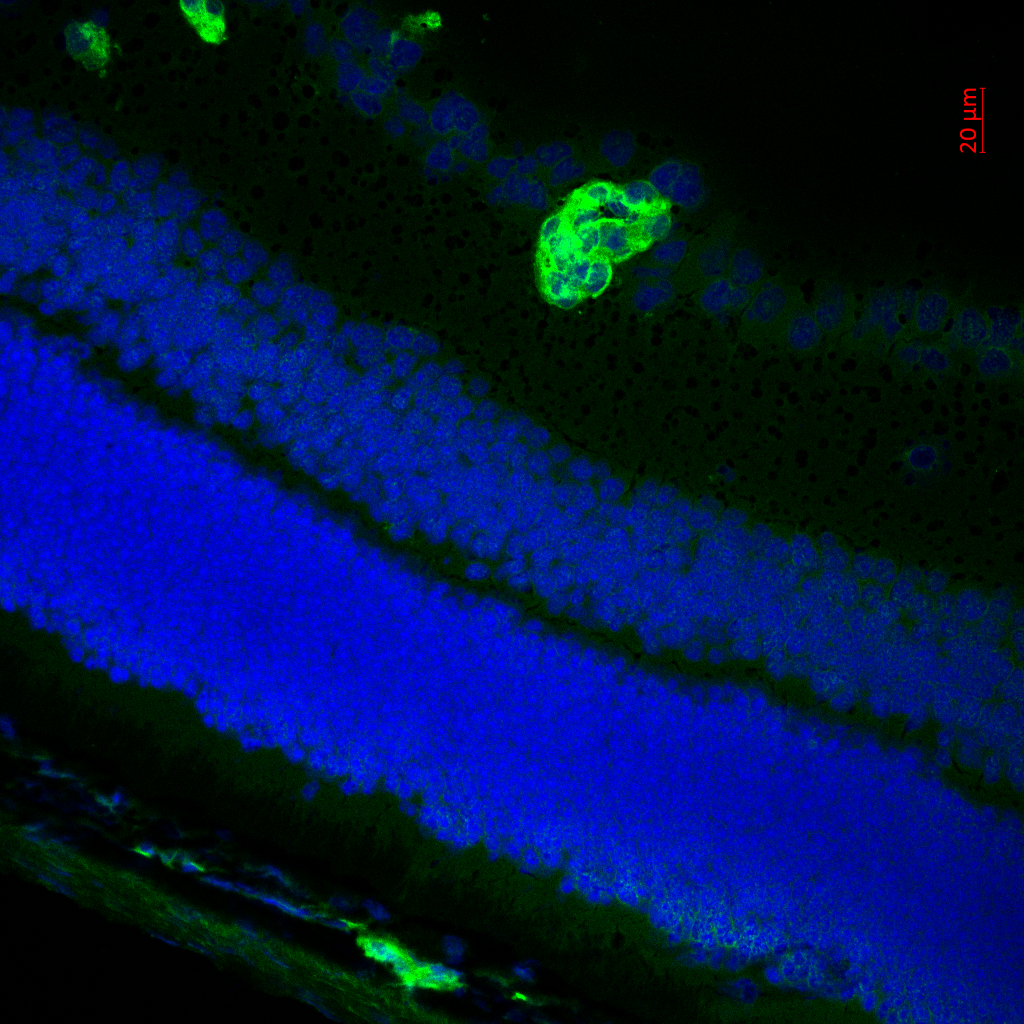

Supplement: Supplementary file 6 — Source Data for Figure 1 [file EMMM-15-e16373-s011.zip › Figure 1/1F/OIR-show.tif]

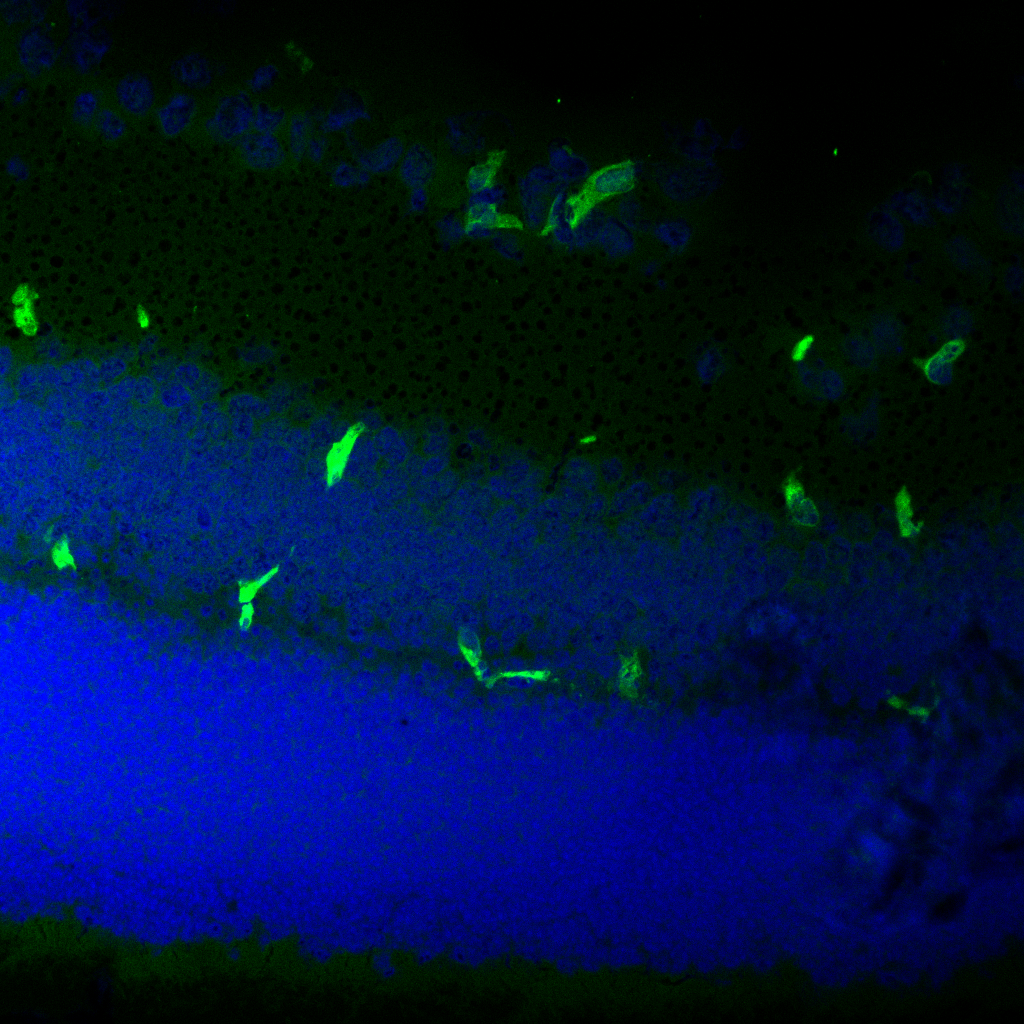

Supplement: Supplementary file 6 — Source Data for Figure 1 [file EMMM-15-e16373-s011.zip › Figure 1/1F/normal-show.tif]

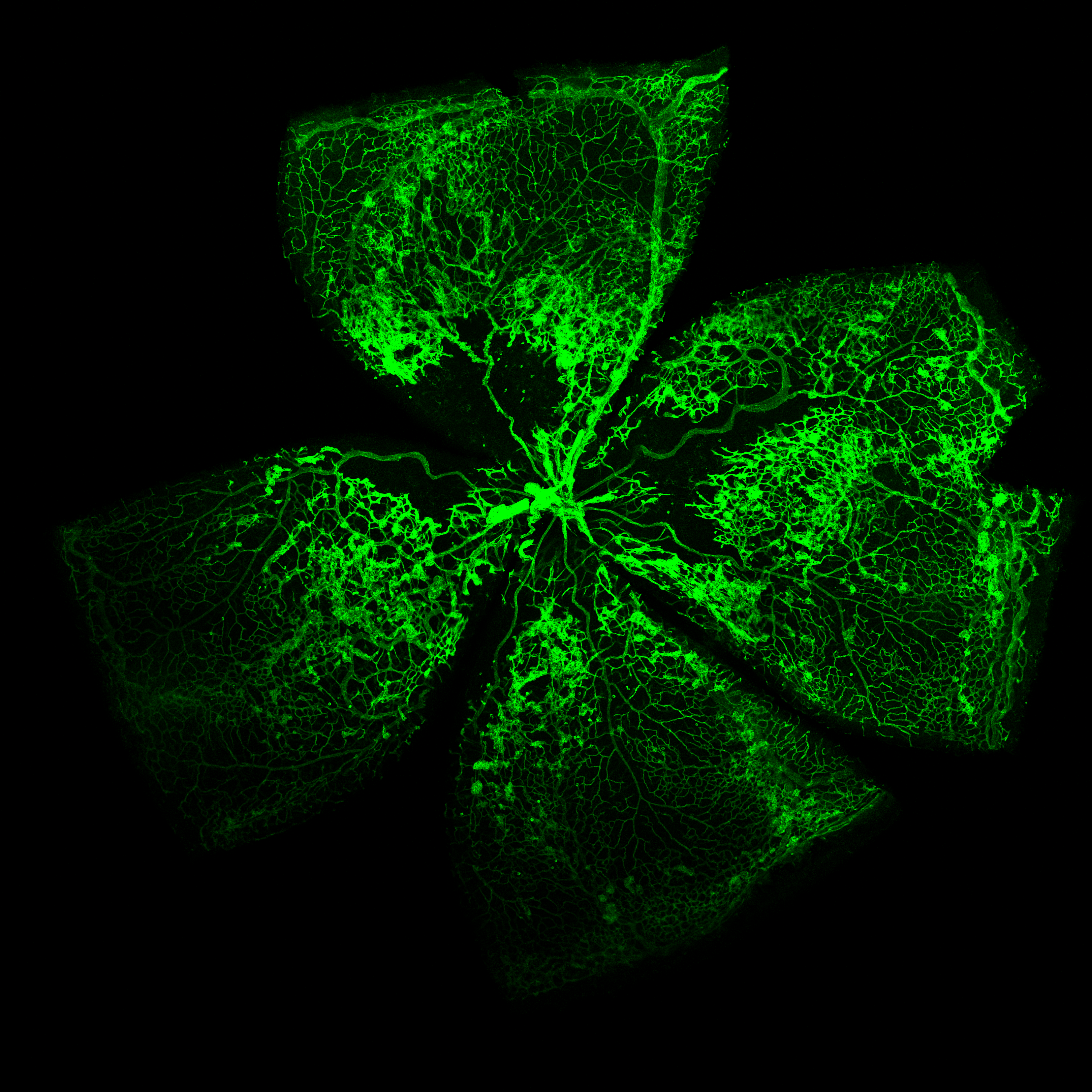

Supplement: Supplementary file 7 — Source Data for Figure 2 [file EMMM-15-e16373-s013.zip › Figure 2/2C/control.tif]

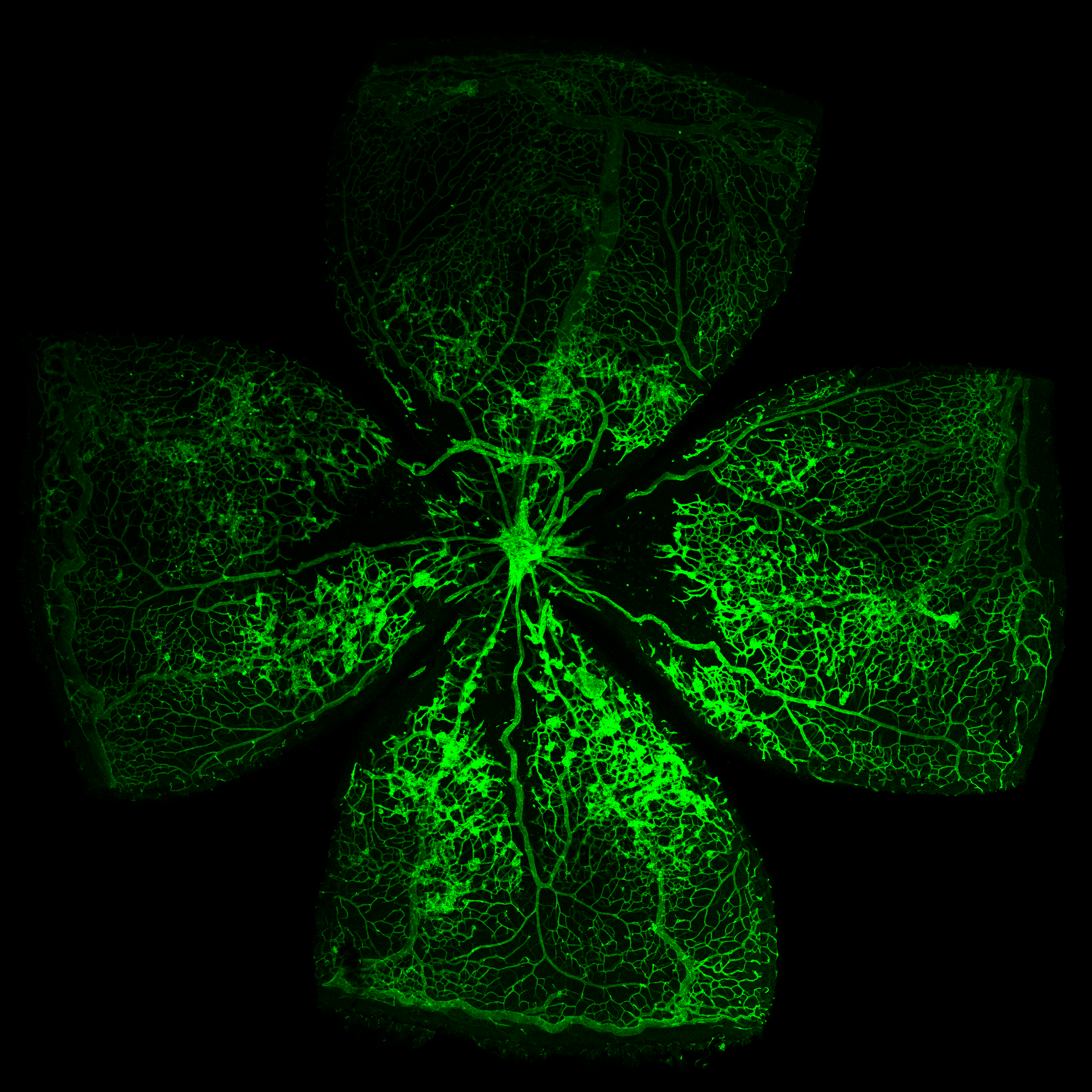

Supplement: Supplementary file 7 — Source Data for Figure 2 [file EMMM-15-e16373-s013.zip › Figure 2/2C/CKO.tif]

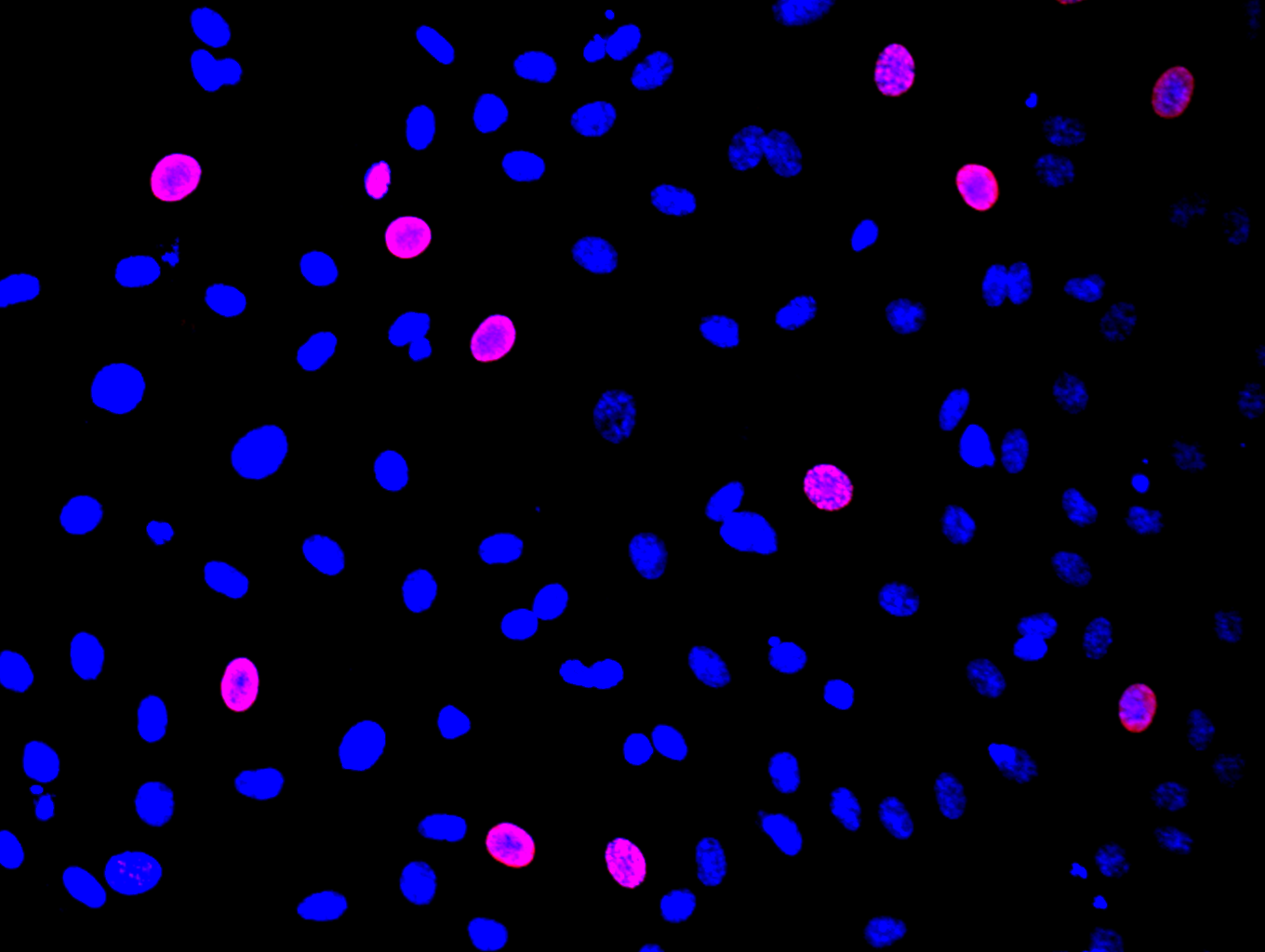

Supplement: Supplementary file 8 — Source Data for Figure 3 [file EMMM-15-e16373-s004.zip › Figure 3/3D.E/3D/PG-3.tif]

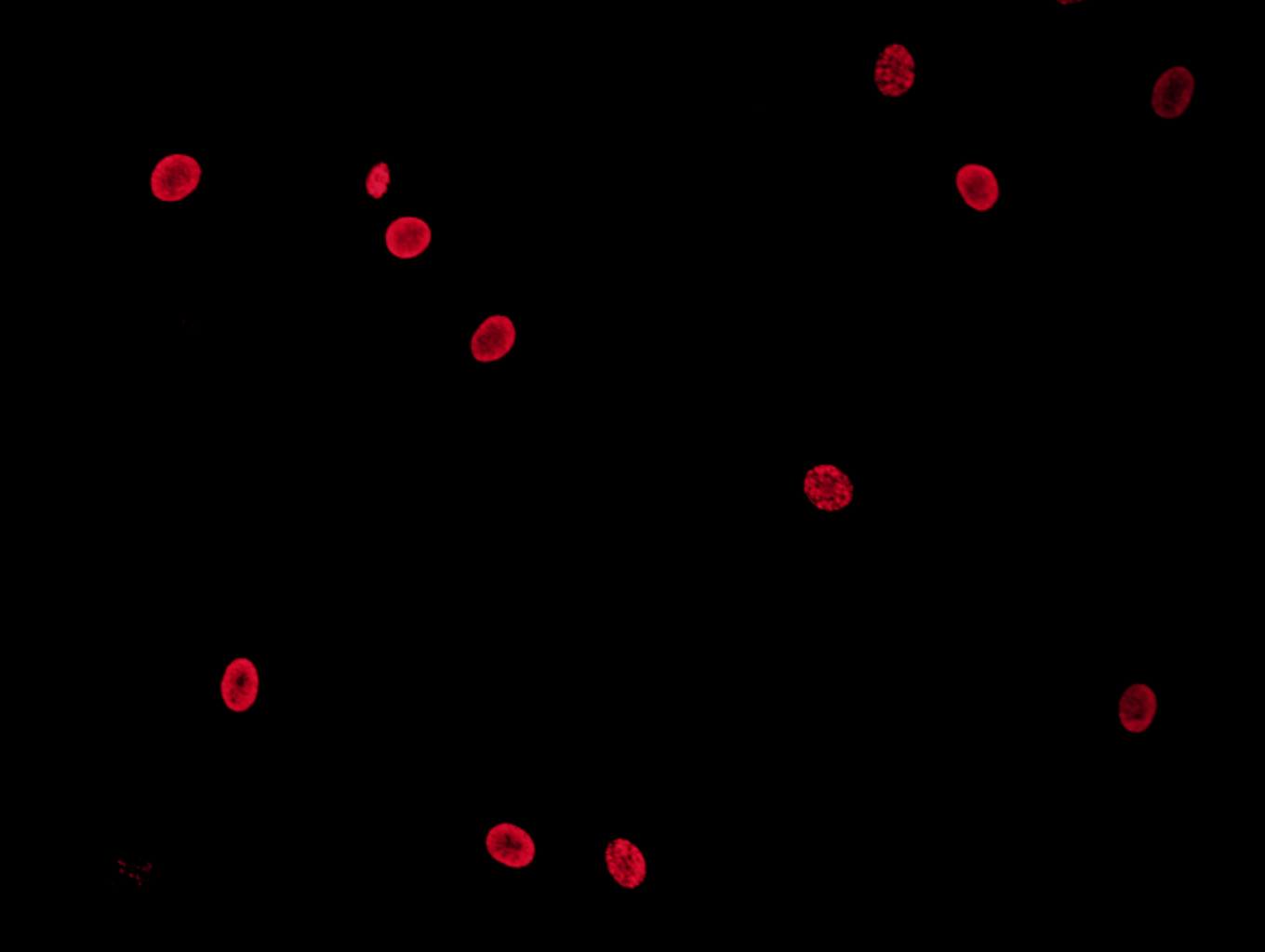

Supplement: Supplementary file 8 — Source Data for Figure 3 [file EMMM-15-e16373-s004.zip › Figure 3/3D.E/3D/PG-2.tif]

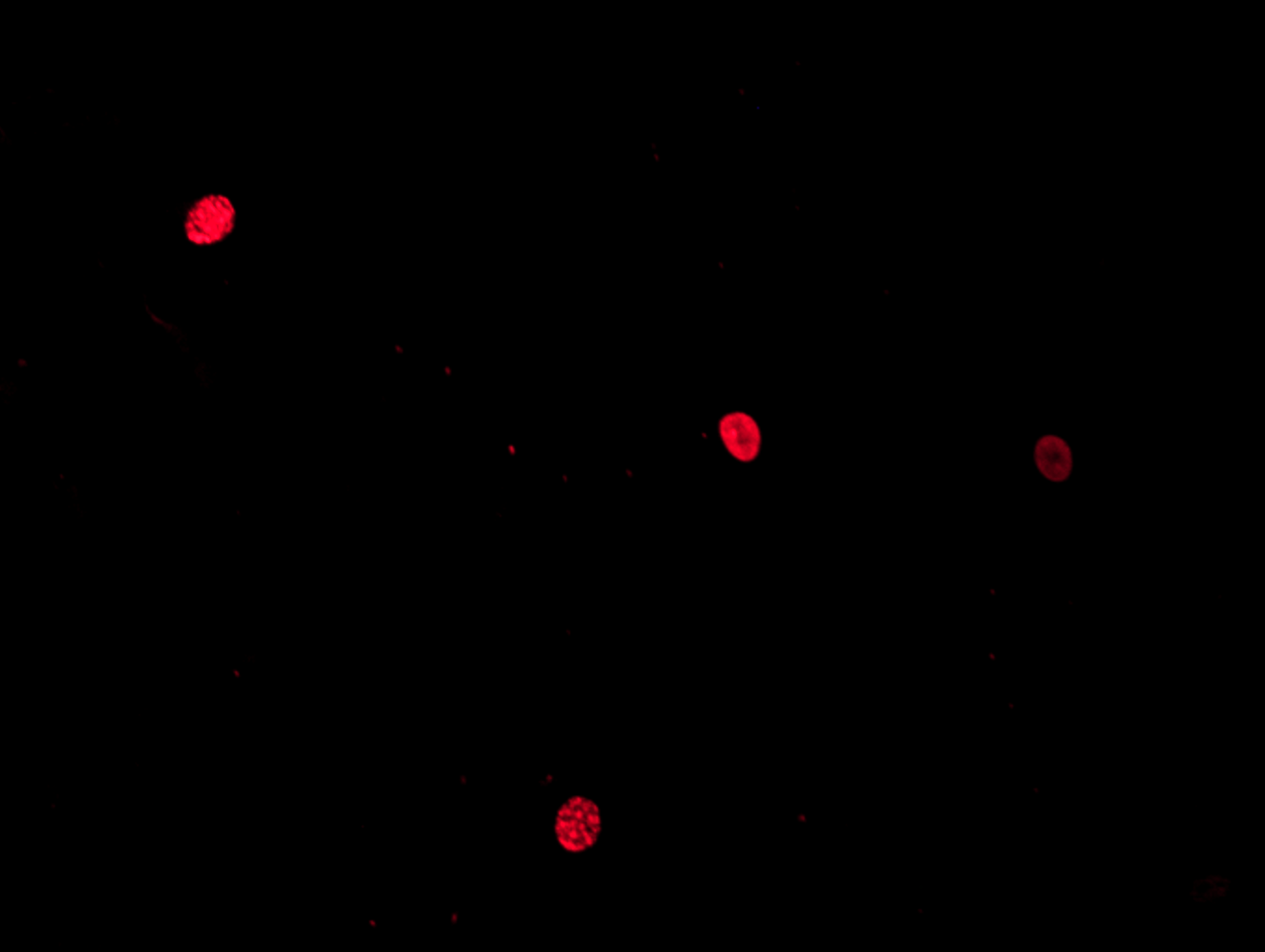

Supplement: Supplementary file 8 — Source Data for Figure 3 [file EMMM-15-e16373-s004.zip › Figure 3/3D.E/3D/DMSO-2.tif]

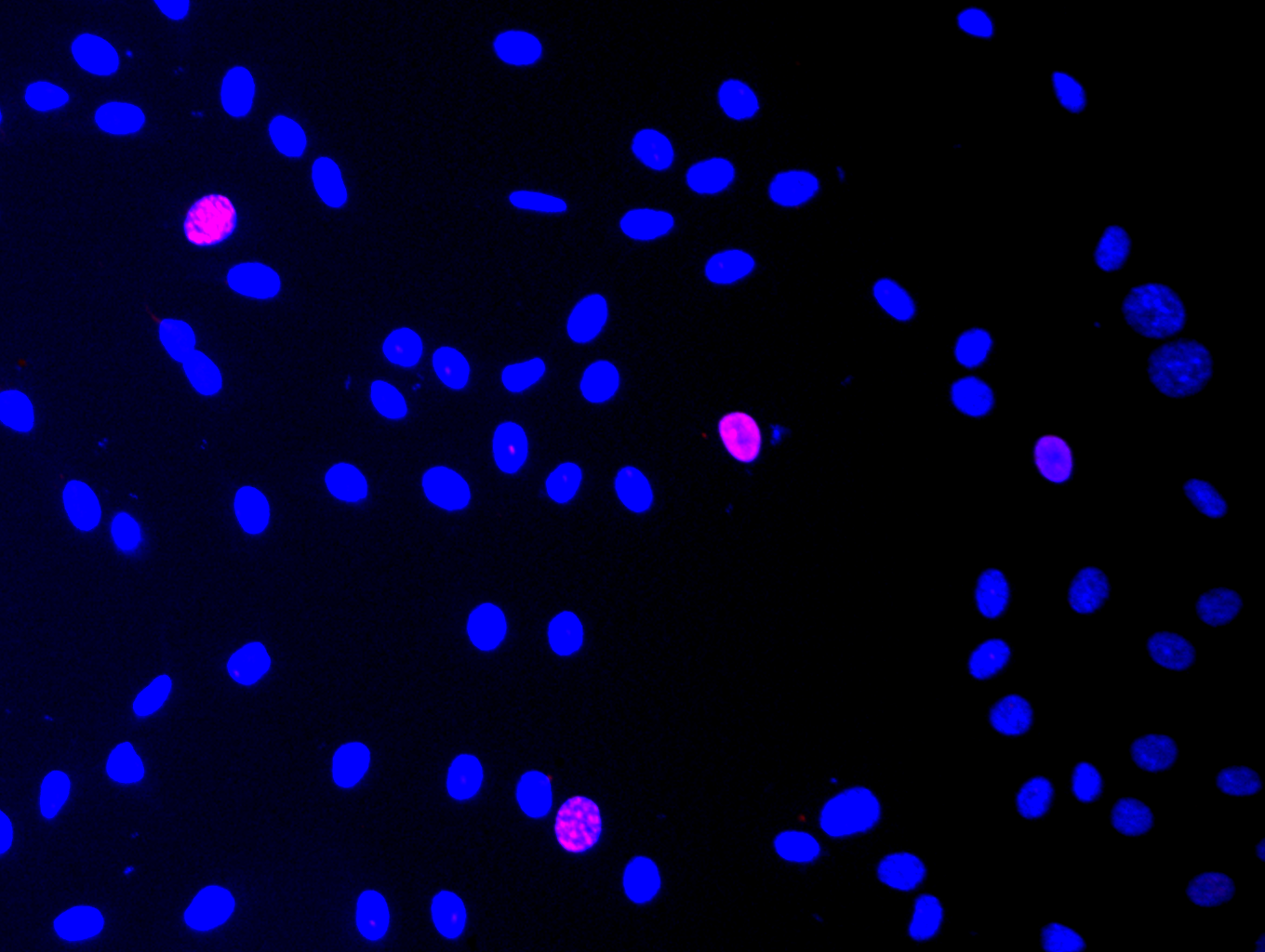

Supplement: Supplementary file 8 — Source Data for Figure 3 [file EMMM-15-e16373-s004.zip › Figure 3/3D.E/3D/DMSO-3.tif]

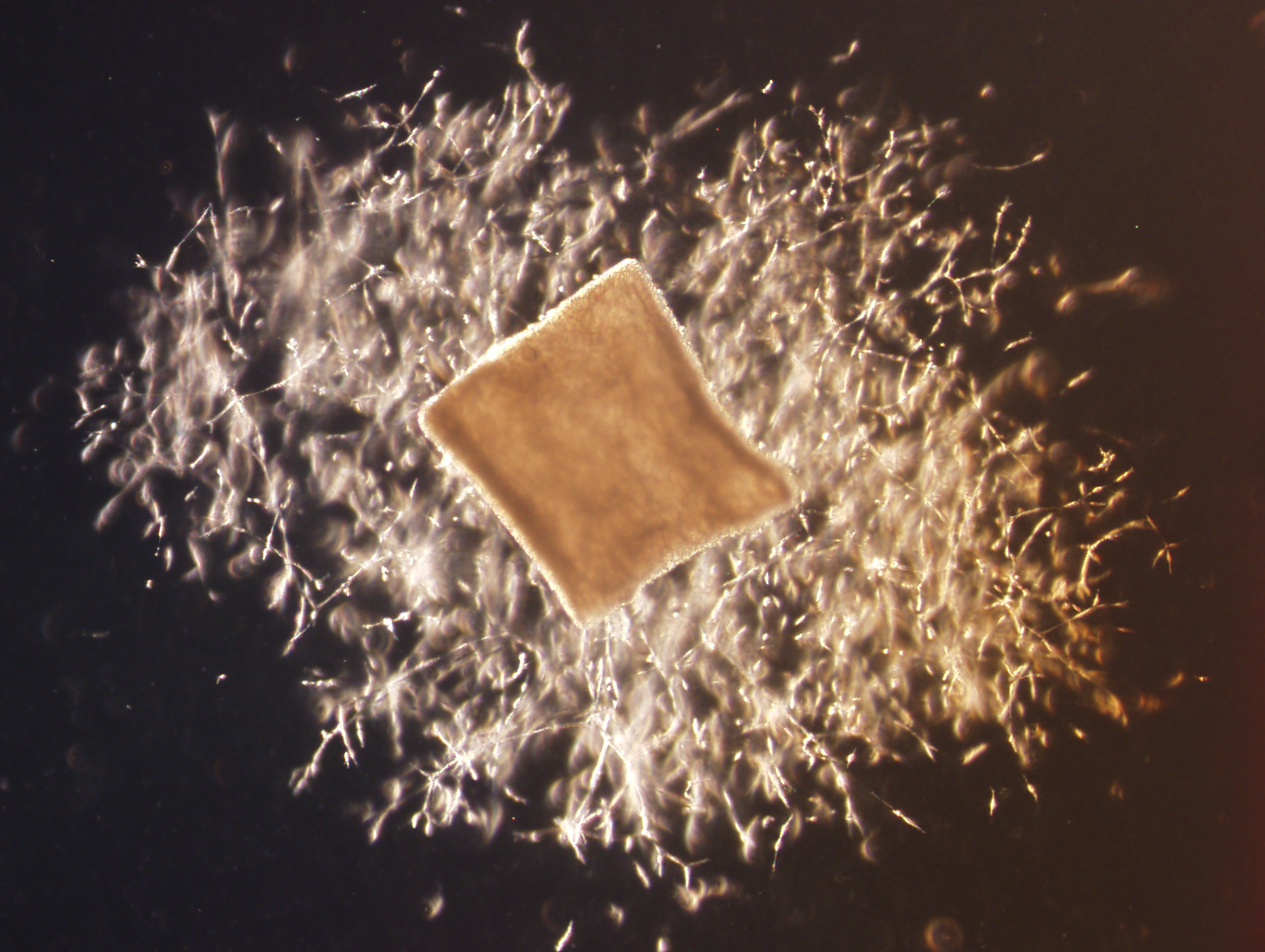

Supplement: Supplementary file 8 — Source Data for Figure 3 [file EMMM-15-e16373-s004.zip › Figure 3/3K.L/PGF.tif]

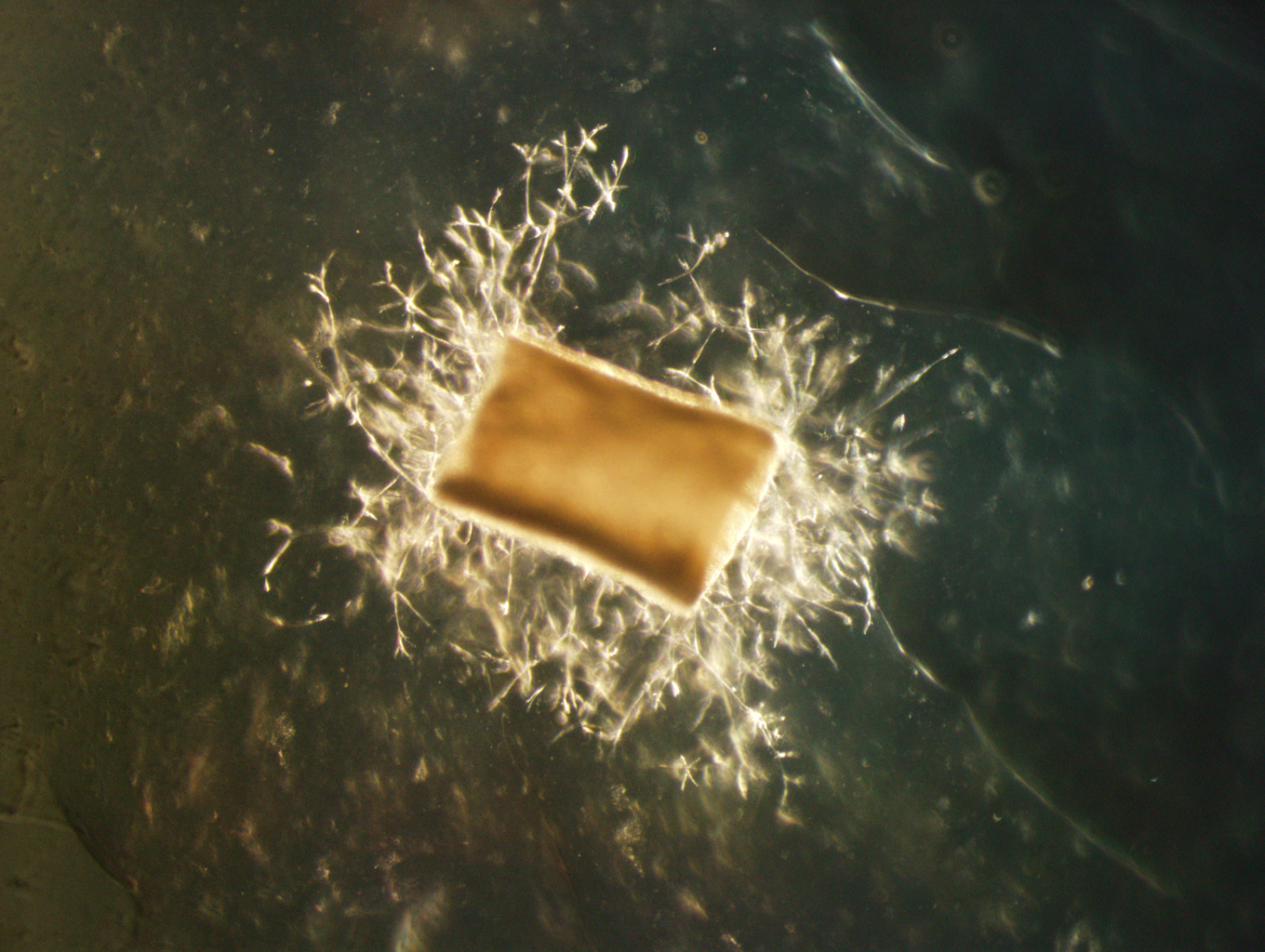

Supplement: Supplementary file 8 — Source Data for Figure 3 [file EMMM-15-e16373-s004.zip › Figure 3/3K.L/DMSO.tif]

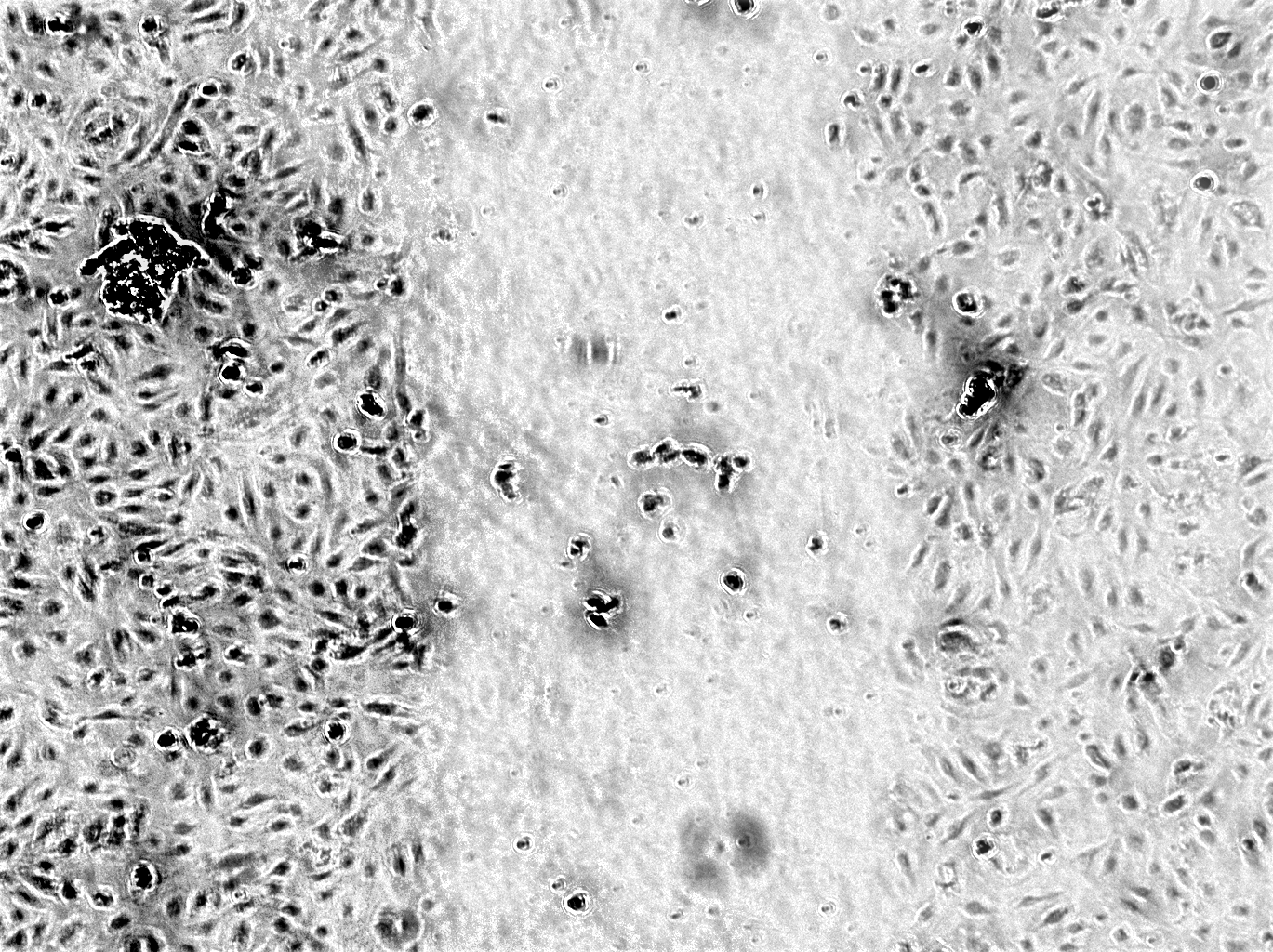

Supplement: Supplementary file 8 — Source Data for Figure 3 [file EMMM-15-e16373-s004.zip › Figure 3/3A.B/PGF.tif]

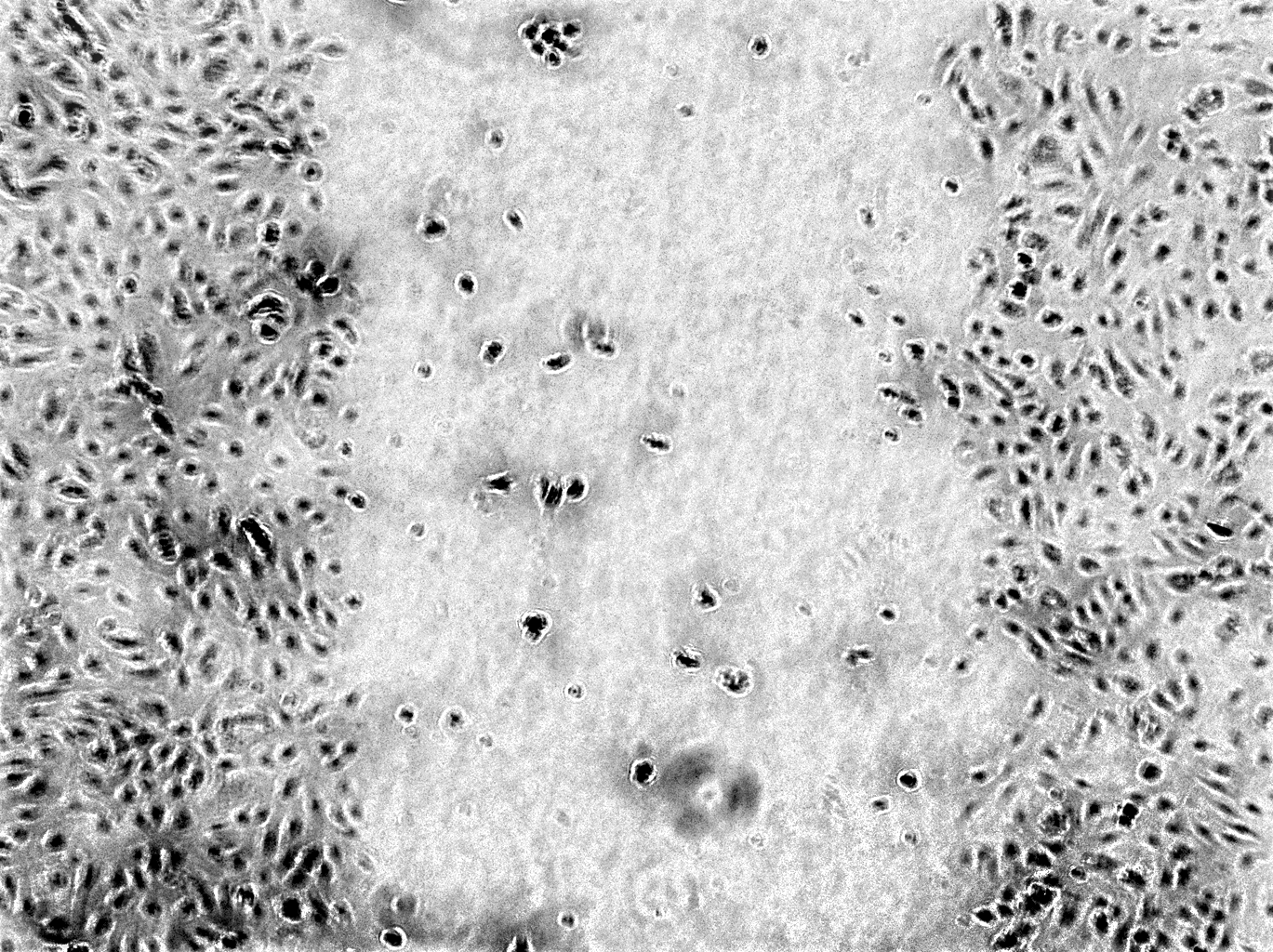

Supplement: Supplementary file 8 — Source Data for Figure 3 [file EMMM-15-e16373-s004.zip › Figure 3/3A.B/DMSO.tif]

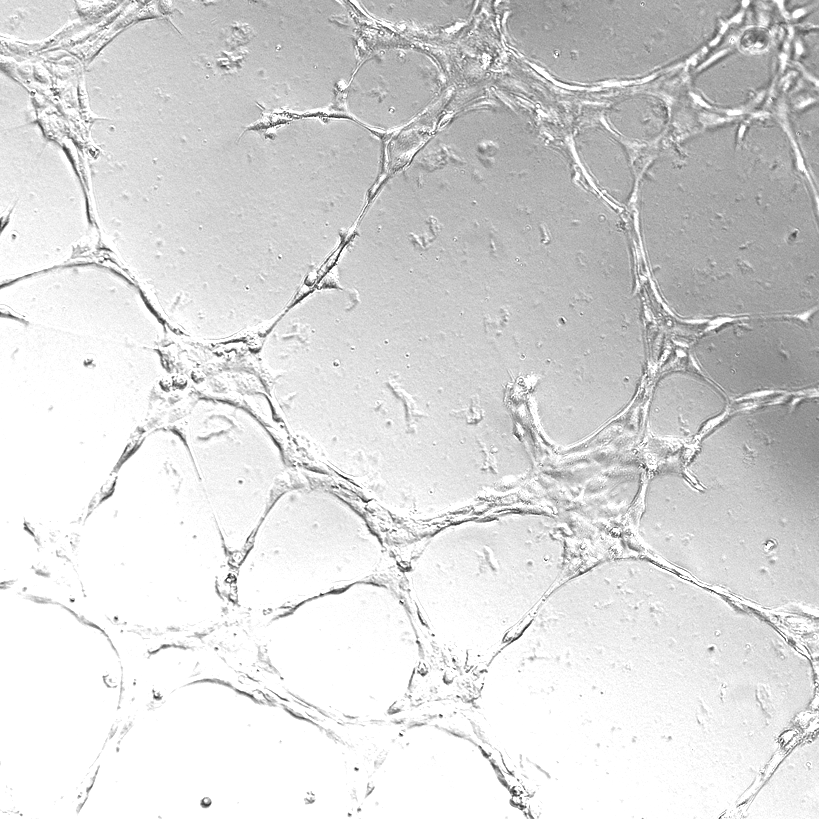

Supplement: Supplementary file 8 — Source Data for Figure 3 [file EMMM-15-e16373-s004.zip › Figure 3/3F.G.H.I.J/DMSO.tif]

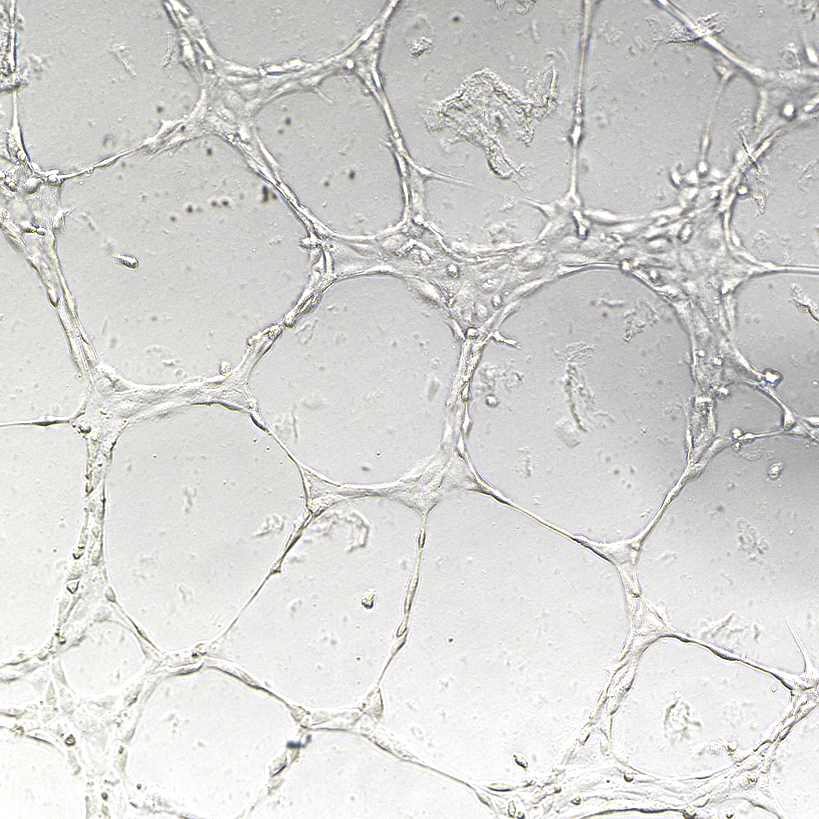

Supplement: Supplementary file 8 — Source Data for Figure 3 [file EMMM-15-e16373-s004.zip › Figure 3/3F.G.H.I.J/PG.tif]

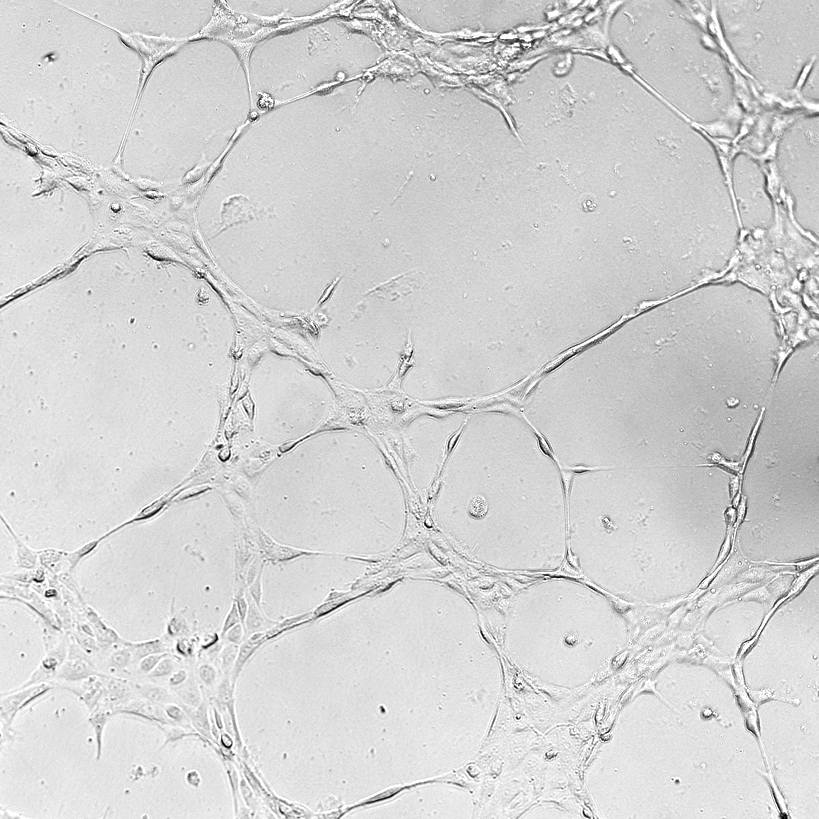

Supplement: Supplementary file 9 — Source Data for Figure 4 [file EMMM-15-e16373-s007.zip › Figure 4/4K.L/DMDM.tif]

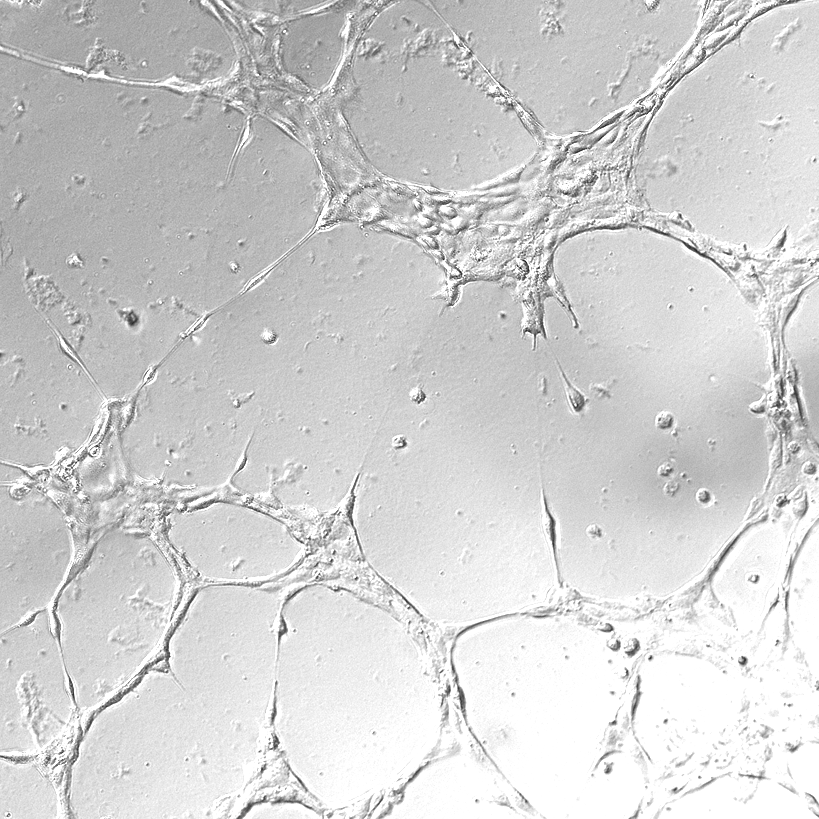

Supplement: Supplementary file 9 — Source Data for Figure 4 [file EMMM-15-e16373-s007.zip › Figure 4/4K.L/SBDM.tif]

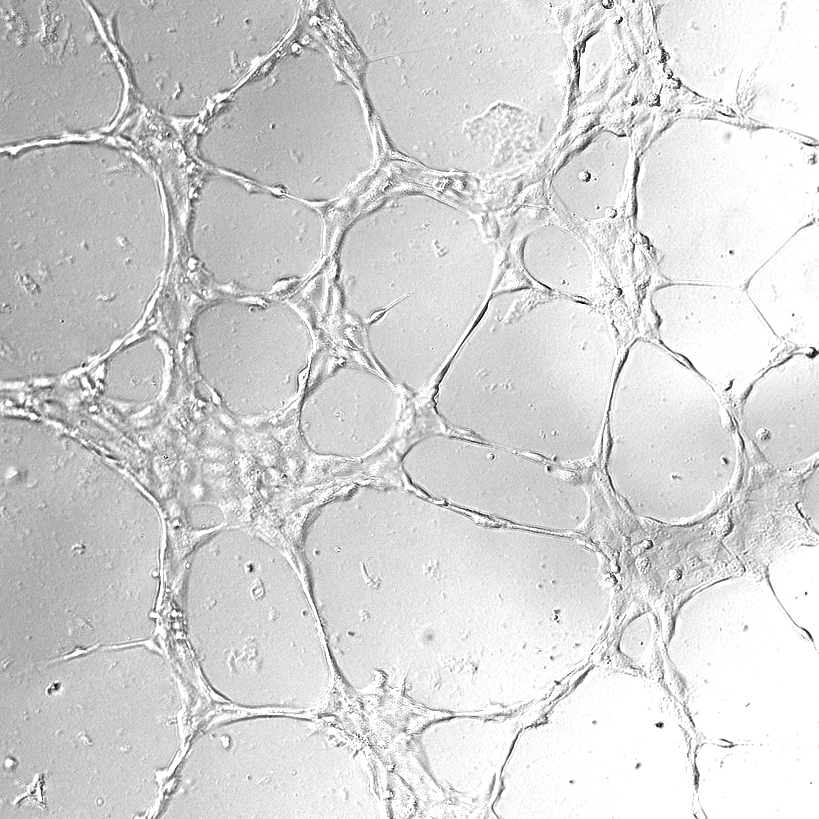

Supplement: Supplementary file 9 — Source Data for Figure 4 [file EMMM-15-e16373-s007.zip › Figure 4/4K.L/DMPG.tif]

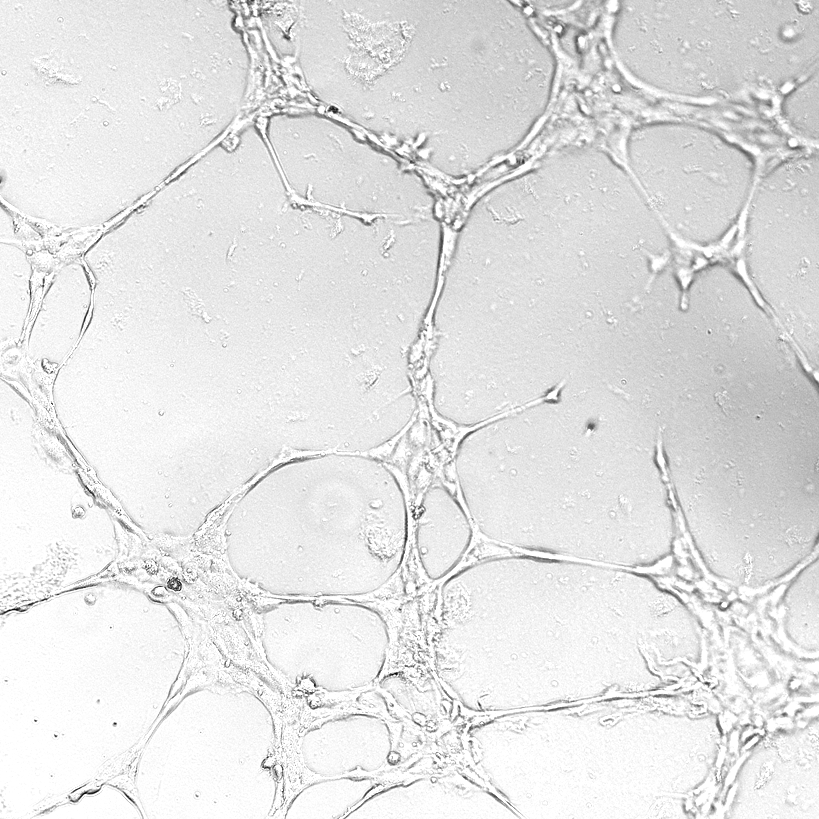

Supplement: Supplementary file 9 — Source Data for Figure 4 [file EMMM-15-e16373-s007.zip › Figure 4/4K.L/SBPG.tif]

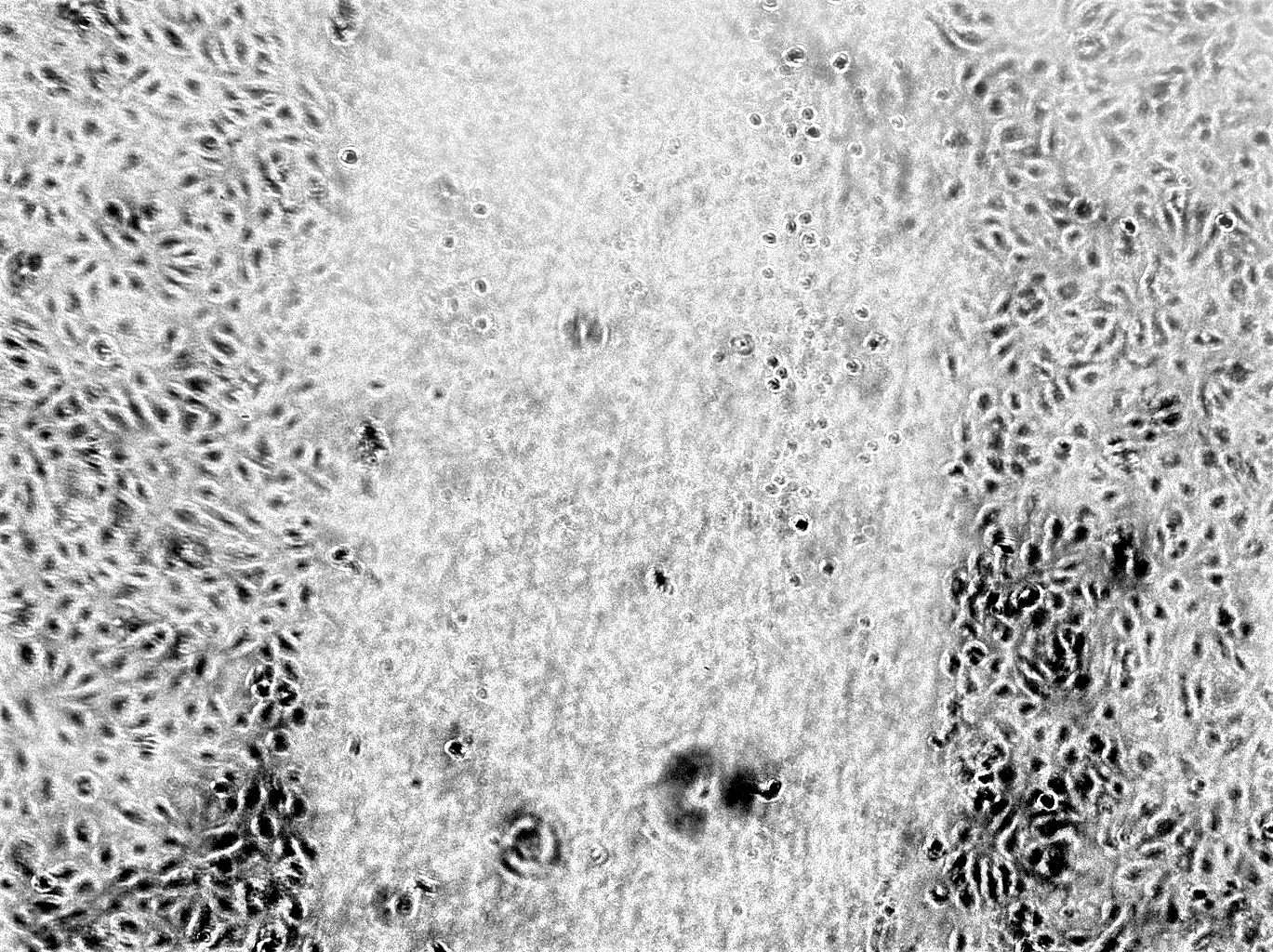

Supplement: Supplementary file 9 — Source Data for Figure 4 [file EMMM-15-e16373-s007.zip › Figure 4/4H.I/DMSO-DMSO.tif]

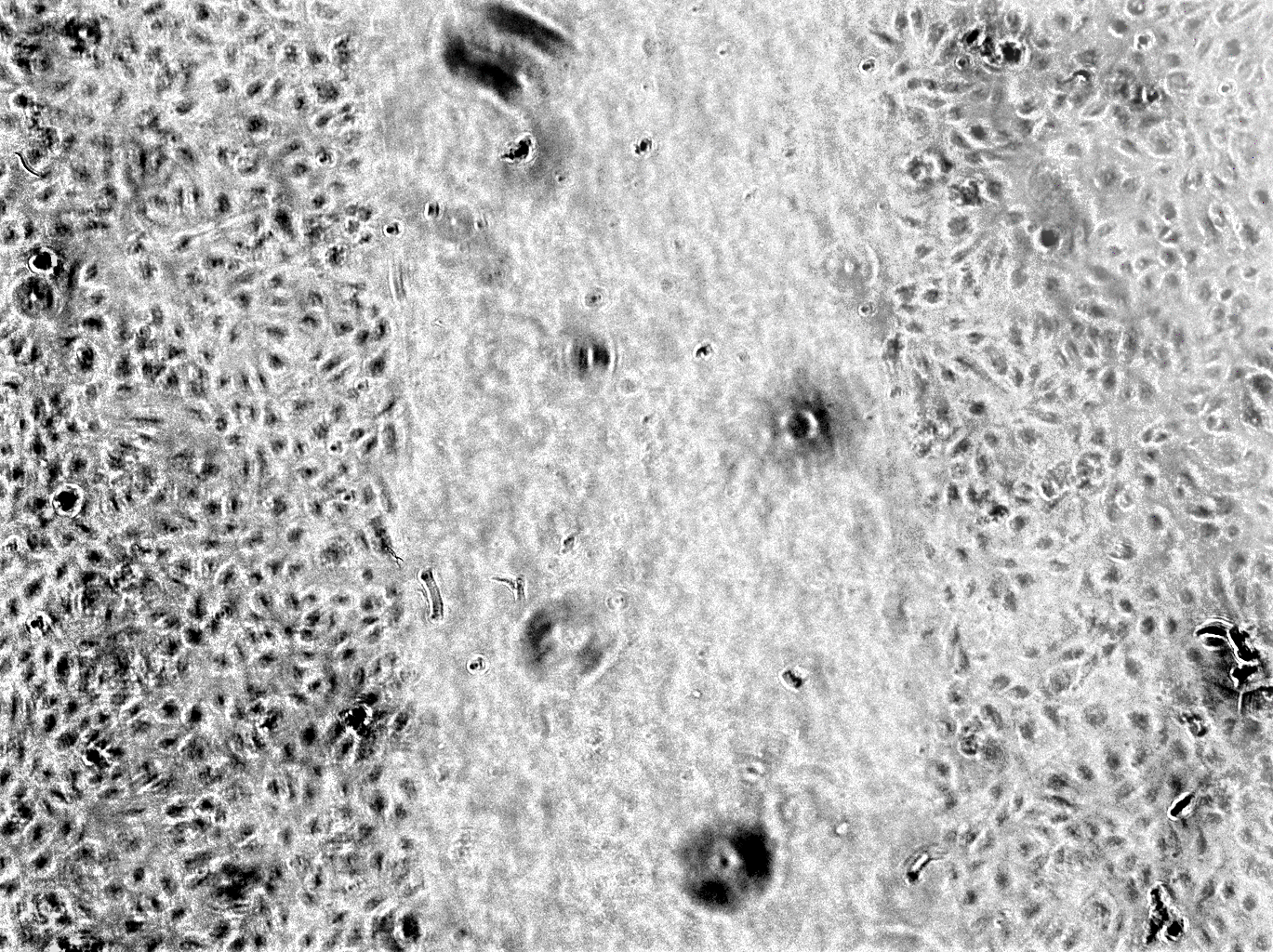

Supplement: Supplementary file 9 — Source Data for Figure 4 [file EMMM-15-e16373-s007.zip › Figure 4/4H.I/DMSO-PGF.tif]

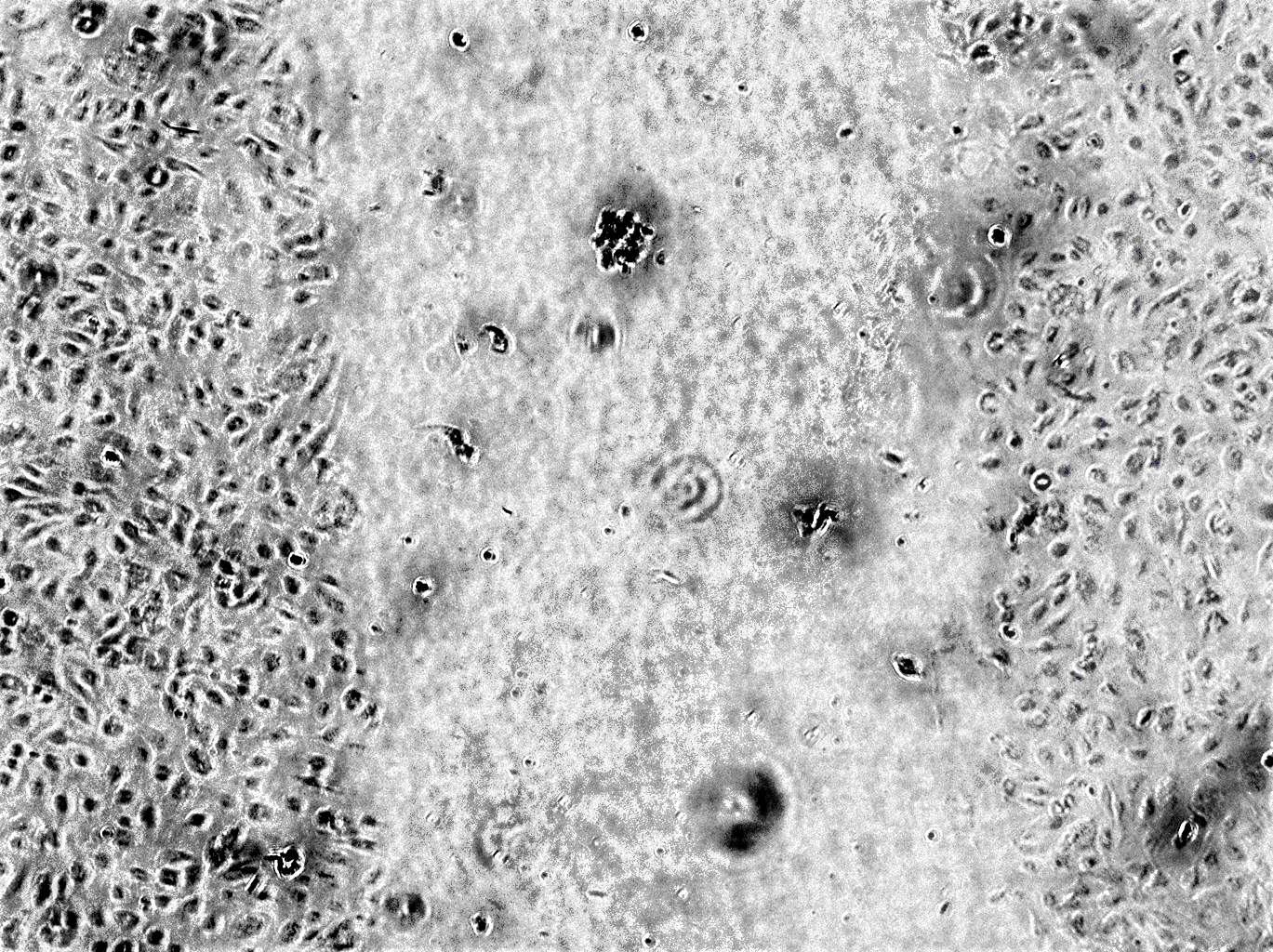

Supplement: Supplementary file 9 — Source Data for Figure 4 [file EMMM-15-e16373-s007.zip › Figure 4/4H.I/PGF-SB265610.tif]

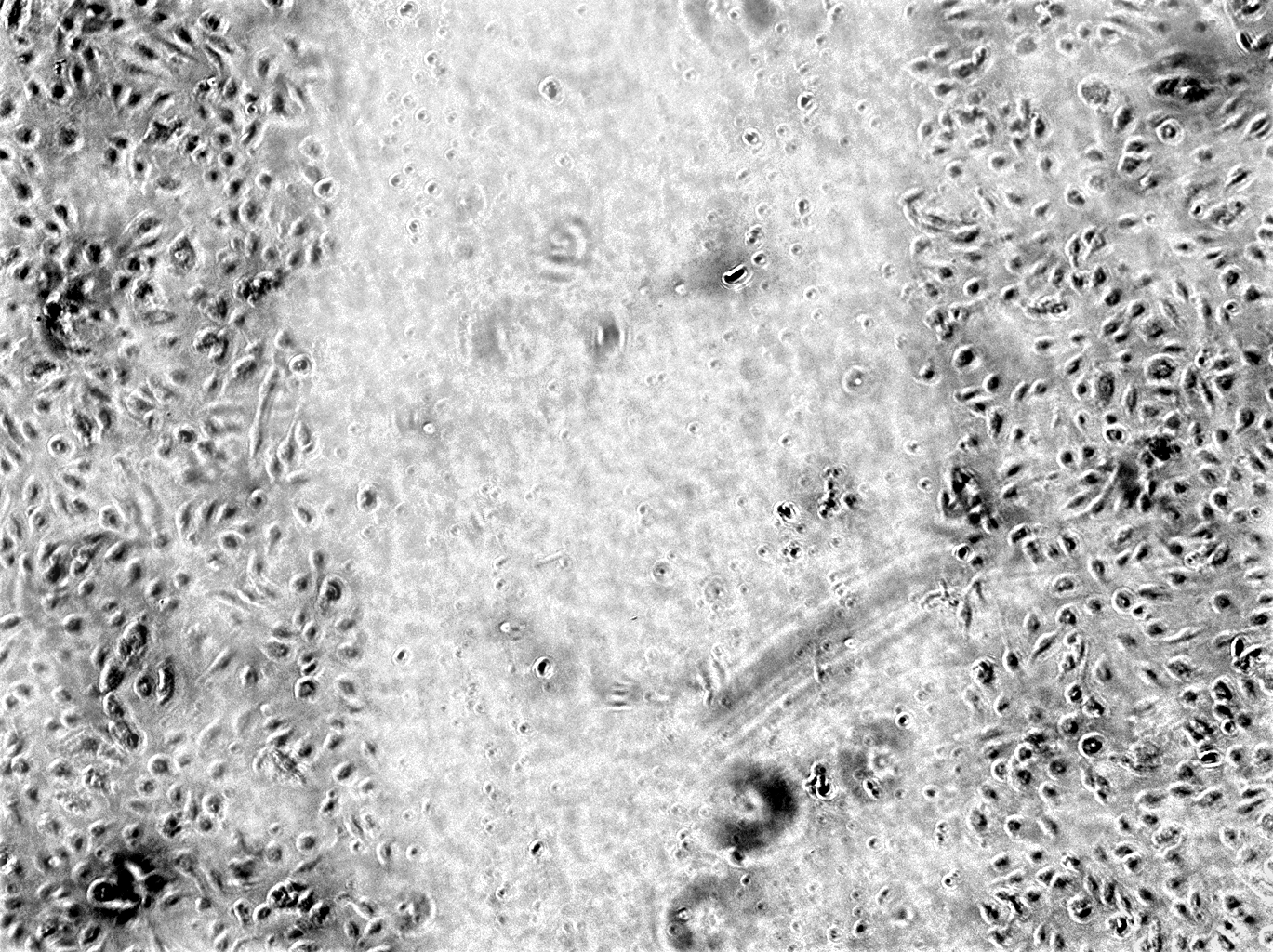

Supplement: Supplementary file 9 — Source Data for Figure 4 [file EMMM-15-e16373-s007.zip › Figure 4/4H.I/DMSO-SB265610.tif]

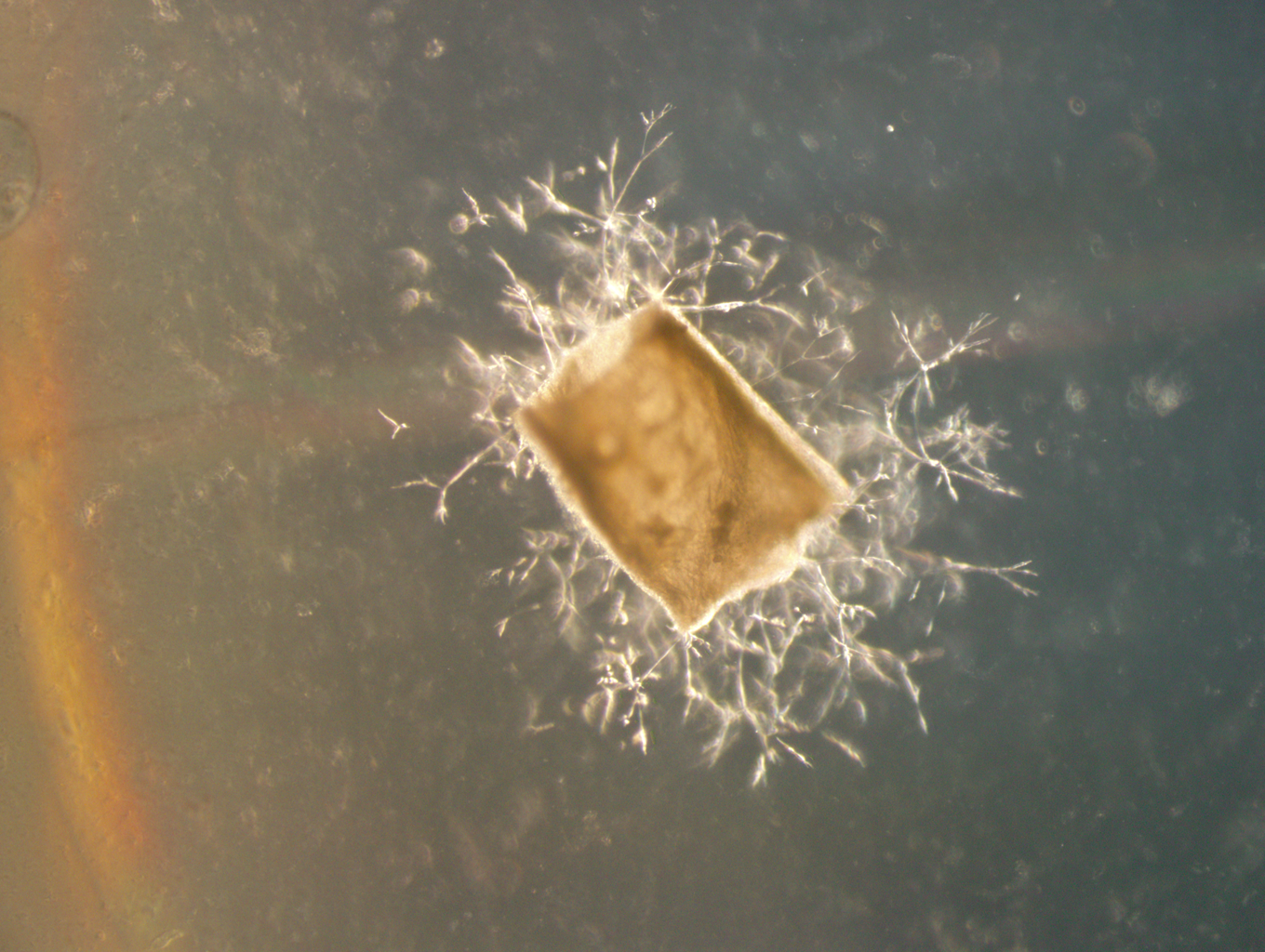

Supplement: Supplementary file 10 — Source Data for Figure 5 [file EMMM-15-e16373-s014.zip › Figure 5/5A.B/SB265610-PGF.tif]

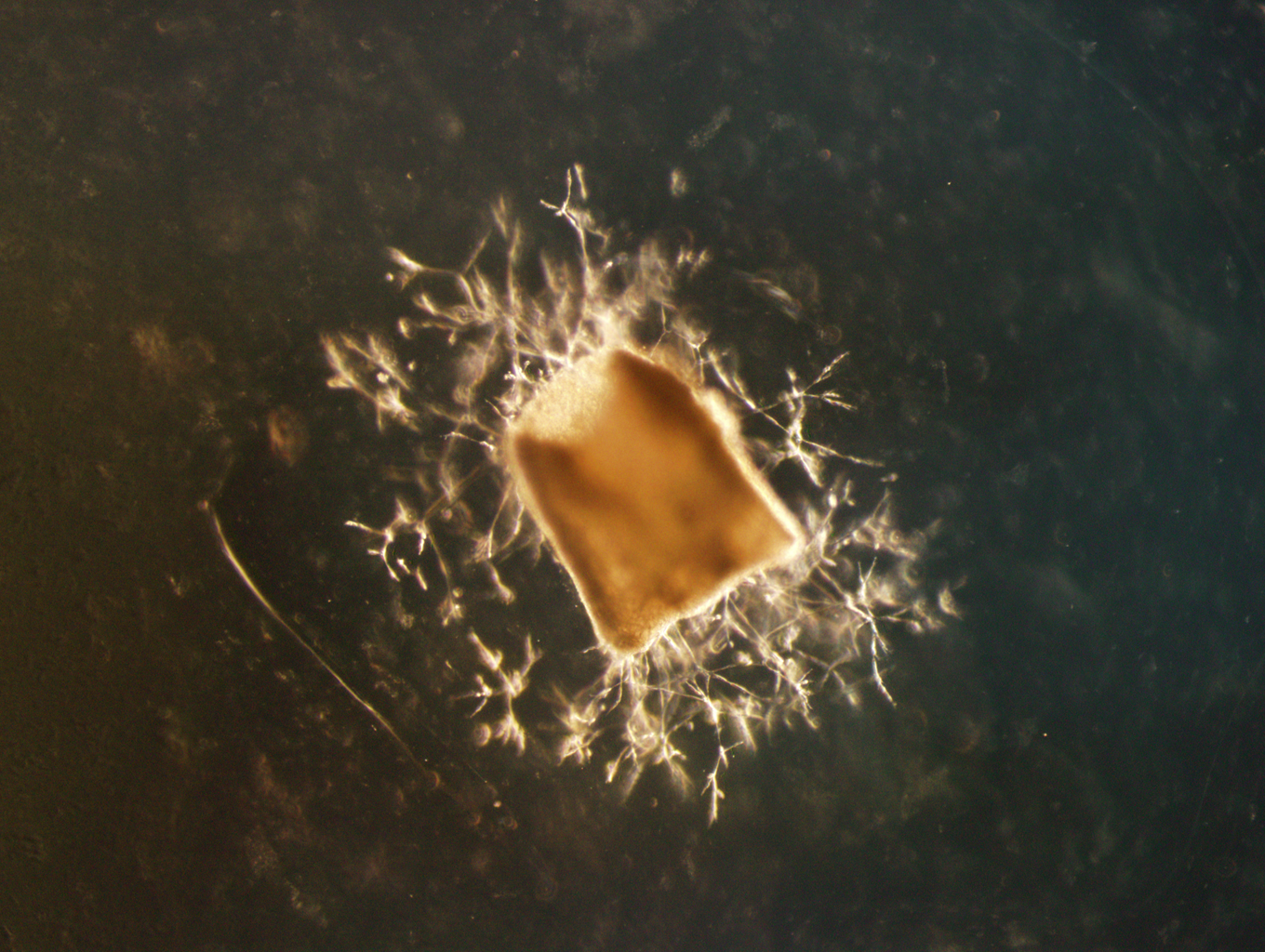

Supplement: Supplementary file 10 — Source Data for Figure 5 [file EMMM-15-e16373-s014.zip › Figure 5/5A.B/DMSO-DMSO.tif]

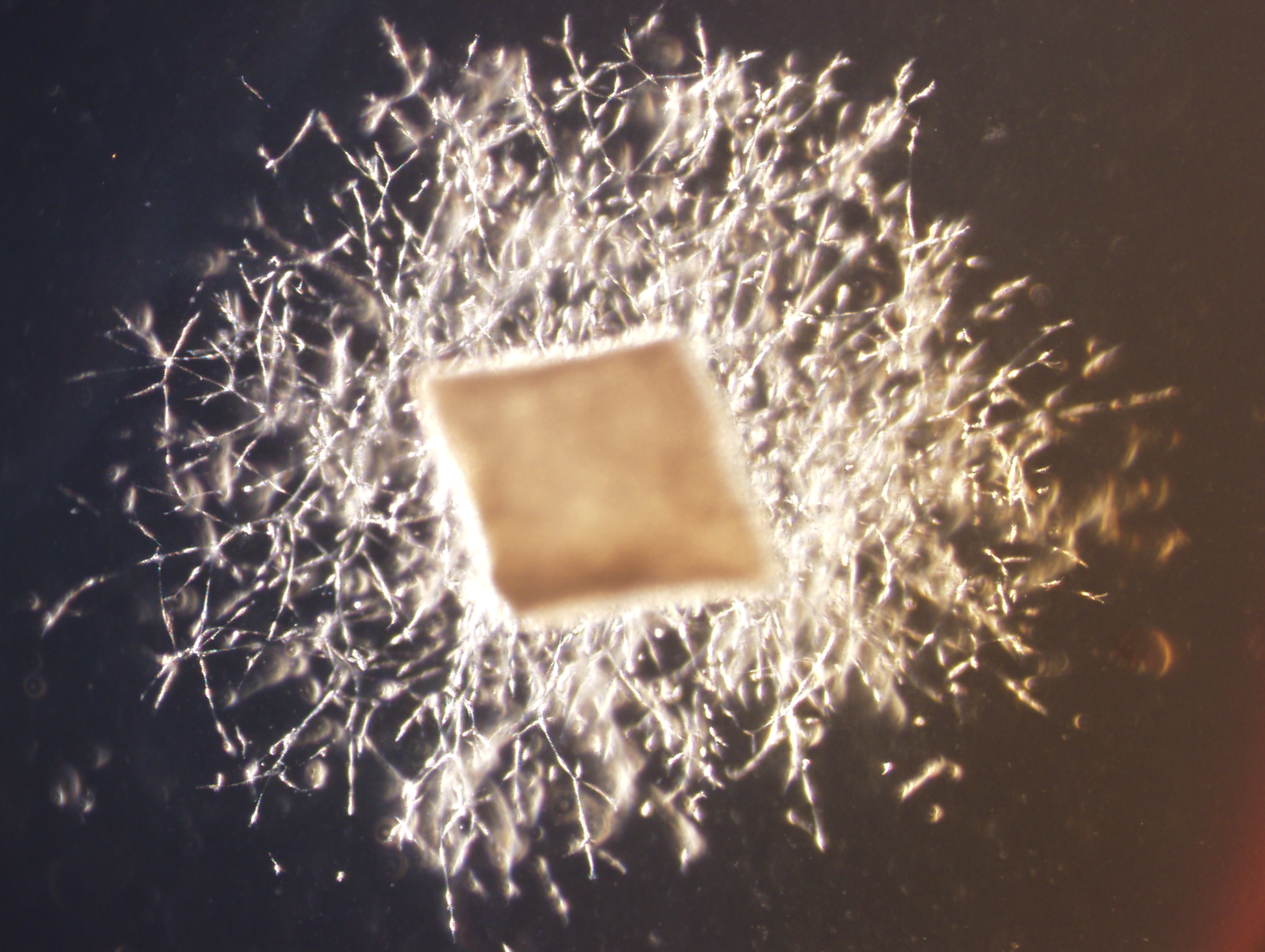

Supplement: Supplementary file 10 — Source Data for Figure 5 [file EMMM-15-e16373-s014.zip › Figure 5/5A.B/DMSO-PGF.tif]

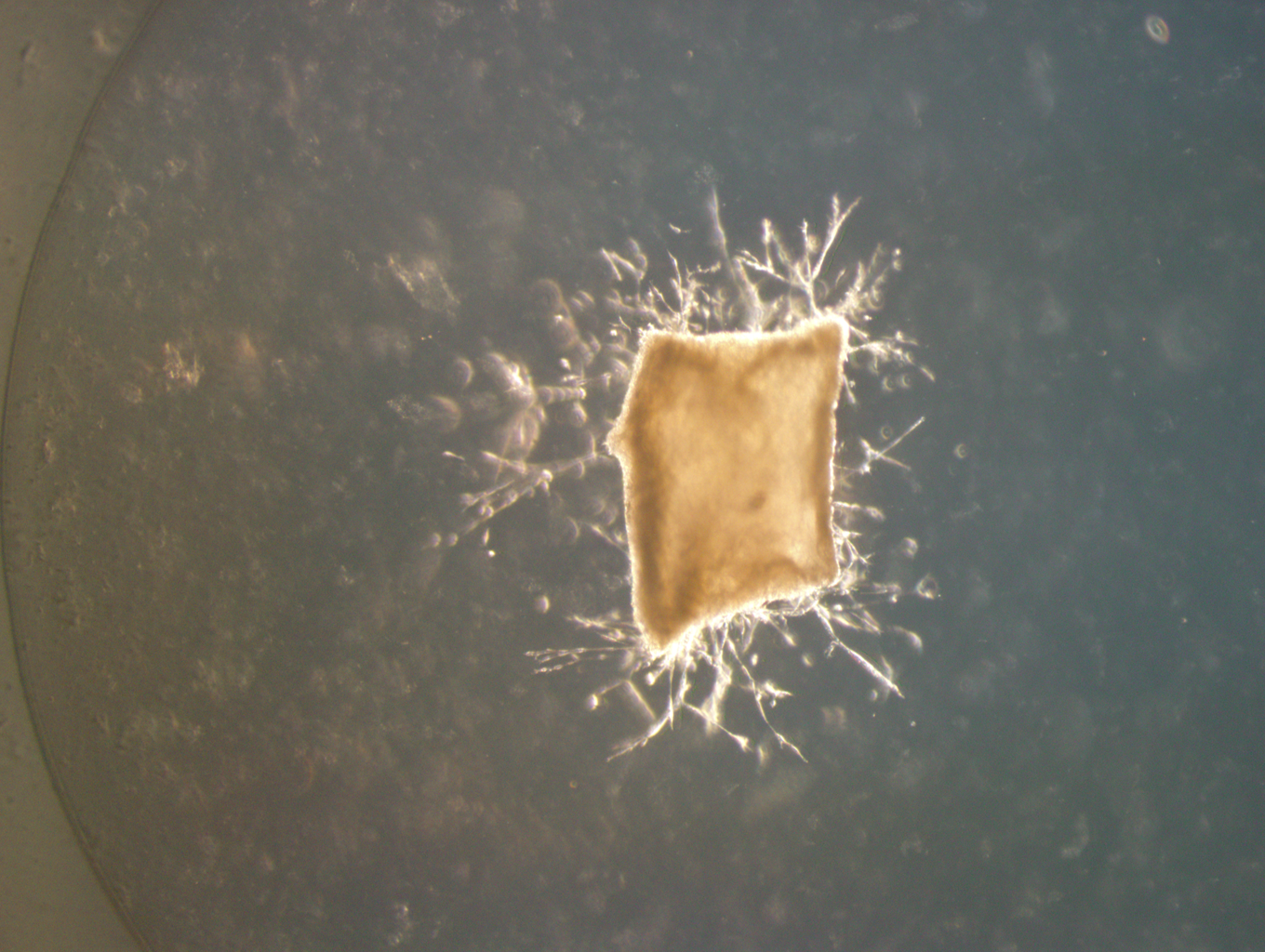

Supplement: Supplementary file 10 — Source Data for Figure 5 [file EMMM-15-e16373-s014.zip › Figure 5/5A.B/DMSO-SB265610.tif]

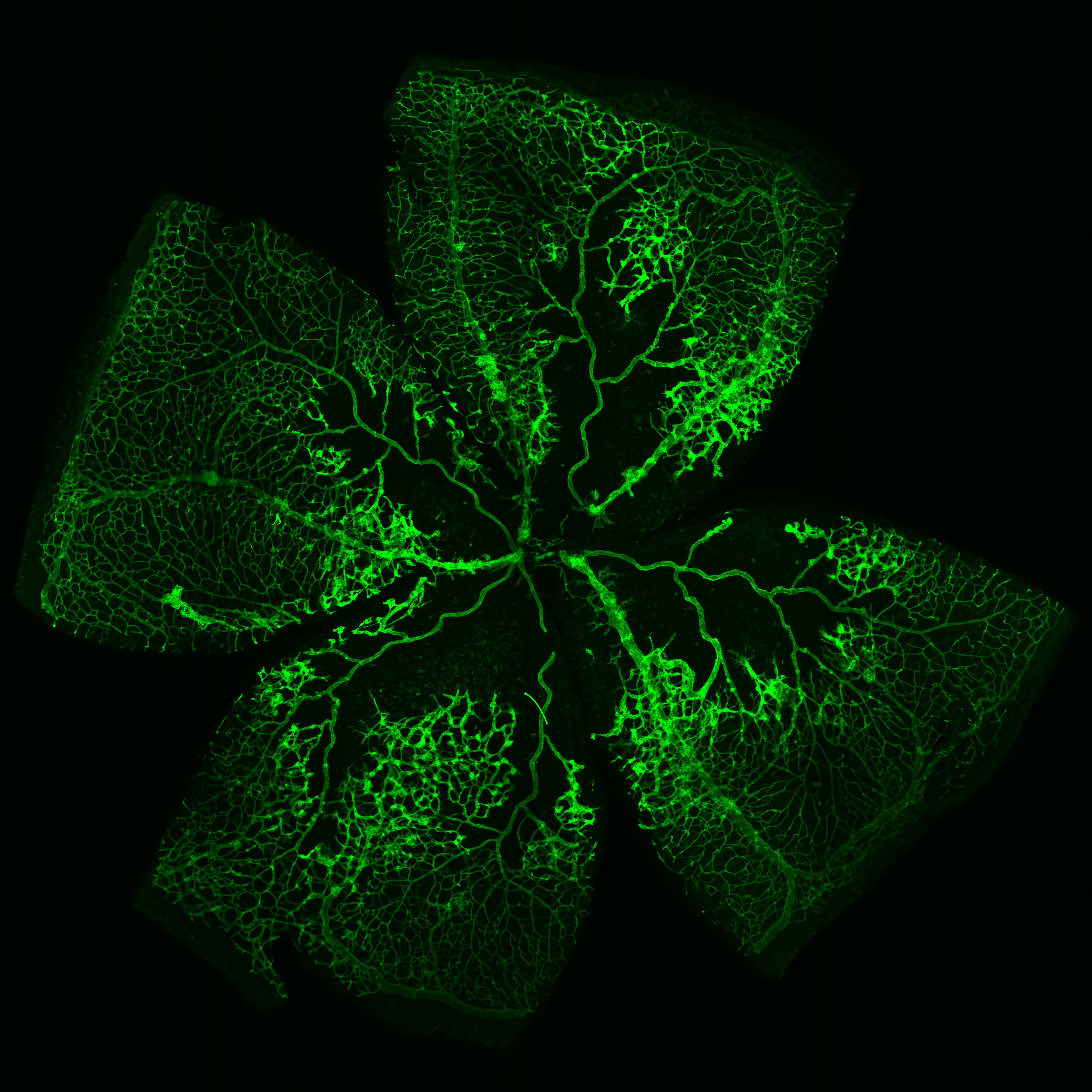

Supplement: Supplementary file 10 — Source Data for Figure 5 [file EMMM-15-e16373-s014.zip › Figure 5/5H.I.J/lenti-con-CKO.tif]

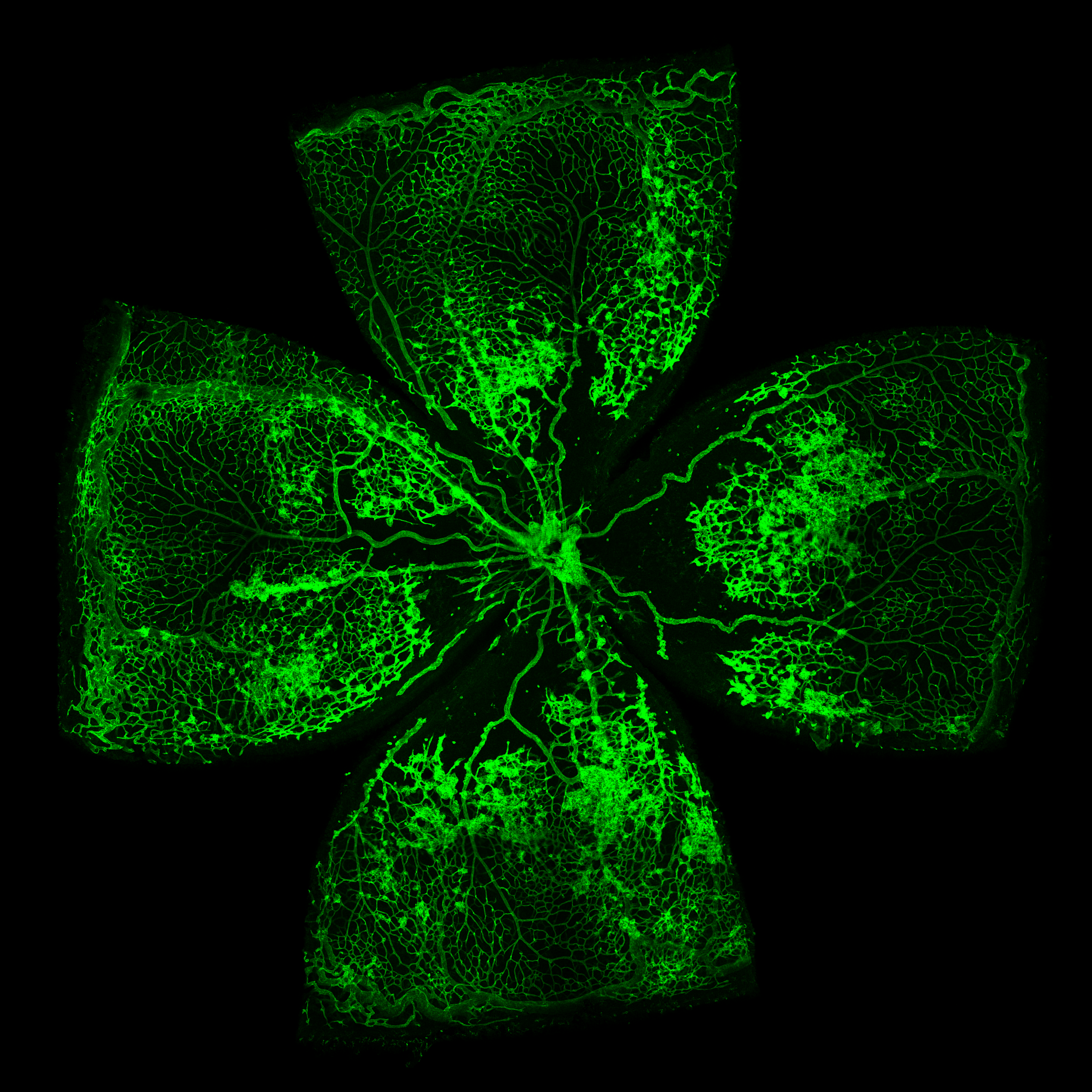

Supplement: Supplementary file 10 — Source Data for Figure 5 [file EMMM-15-e16373-s014.zip › Figure 5/5H.I.J/lenti-cxcl1-CKO.tif]

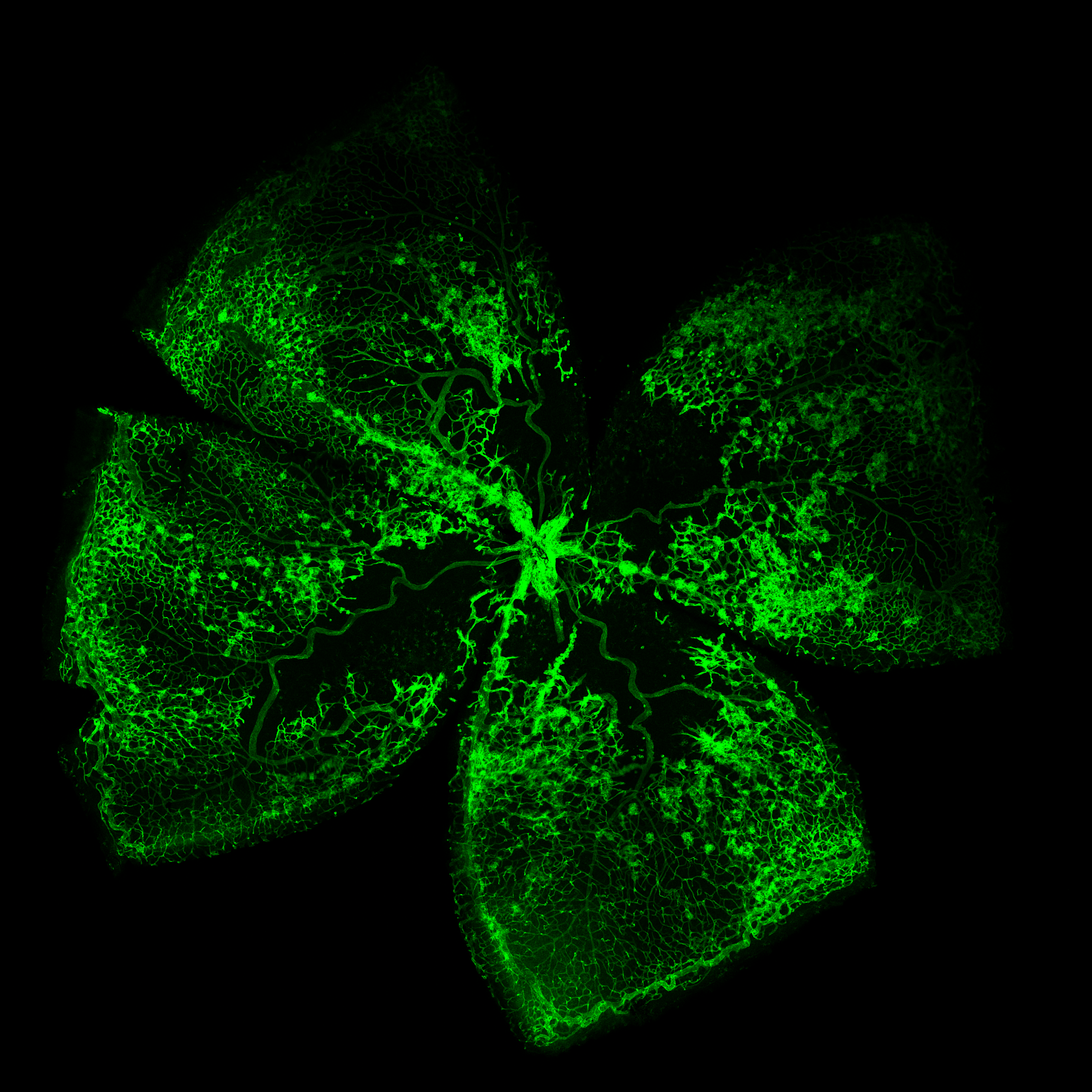

Supplement: Supplementary file 10 — Source Data for Figure 5 [file EMMM-15-e16373-s014.zip › Figure 5/5H.I.J/lenti-cxcl1-control.tif]

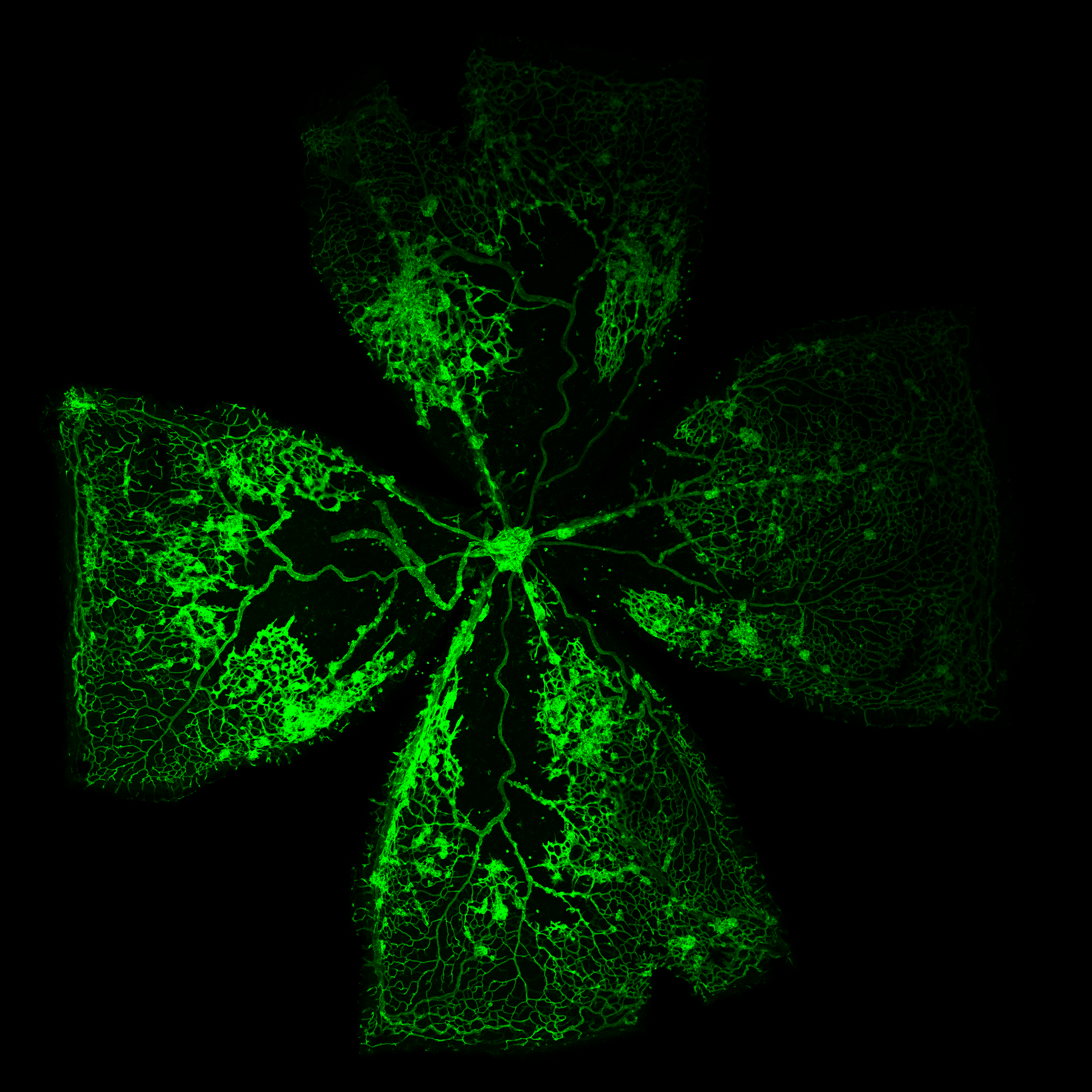

Supplement: Supplementary file 10 — Source Data for Figure 5 [file EMMM-15-e16373-s014.zip › Figure 5/5H.I.J/lenti-con-control.tif]

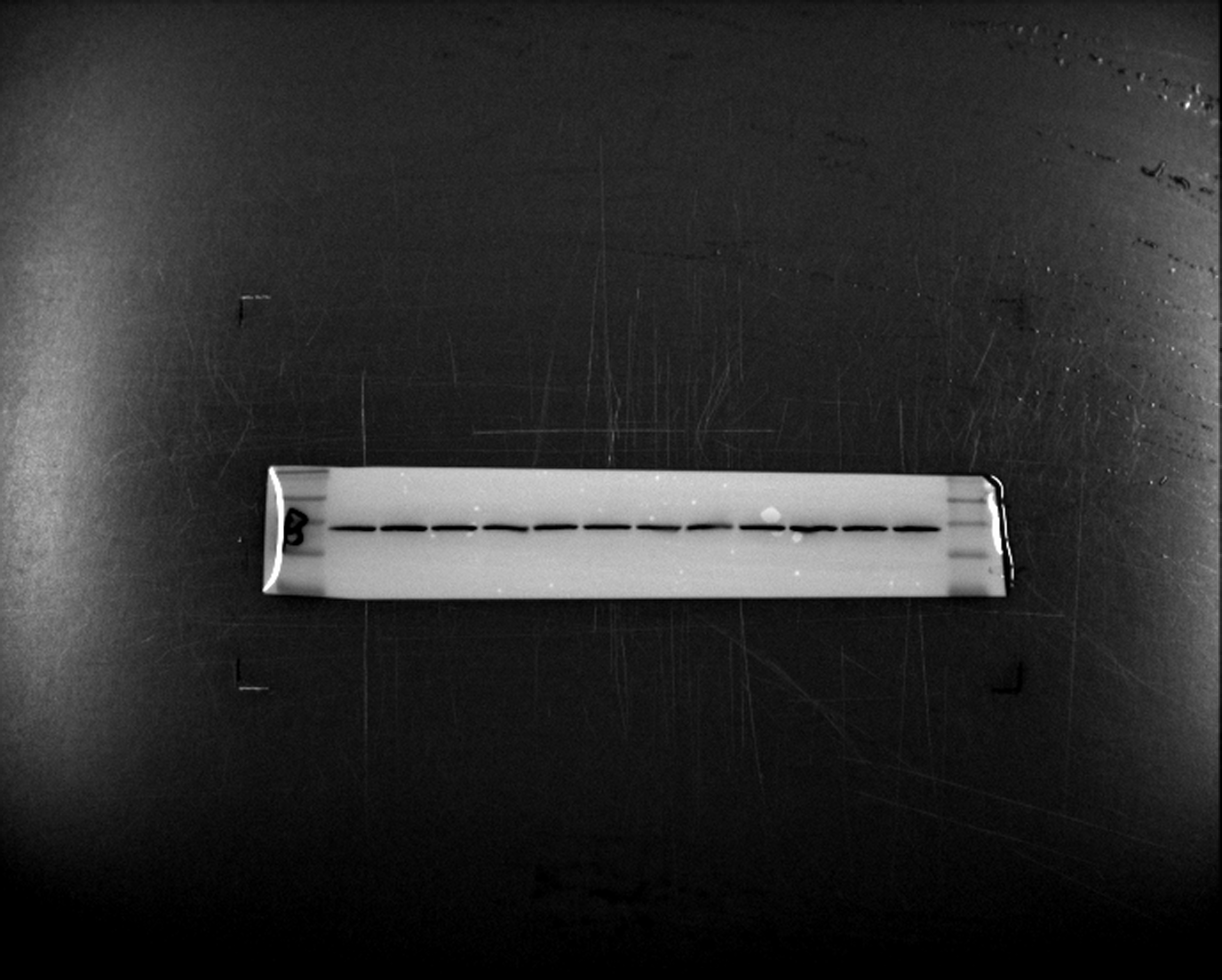

Supplement: Supplementary file 10 — Source Data for Figure 5 [file EMMM-15-e16373-s014.zip › Figure 5/5F.G/6.Tif]

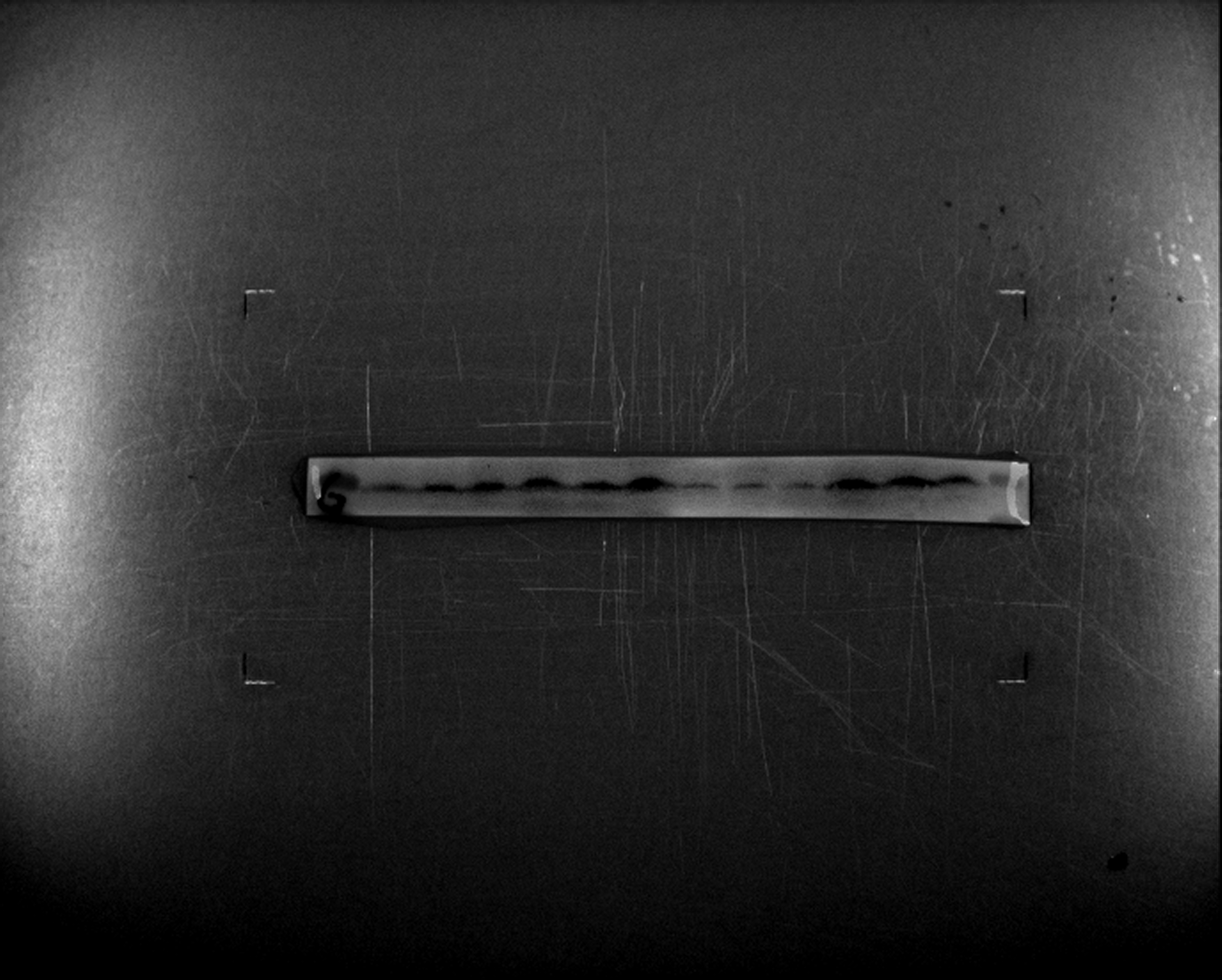

Supplement: Supplementary file 10 — Source Data for Figure 5 [file EMMM-15-e16373-s014.zip › Figure 5/5F.G/24.Tif]

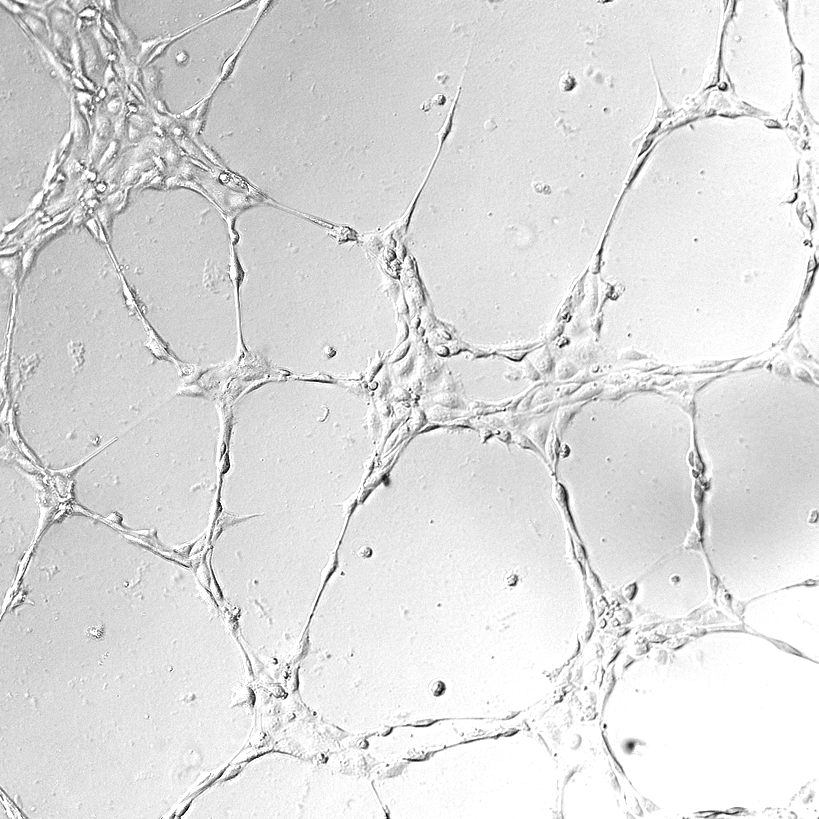

Supplement: Supplementary file 11 — Source Data for Figure 6 [file EMMM-15-e16373-s010.zip › Figure 6/6J.K/lentiPG.tif]

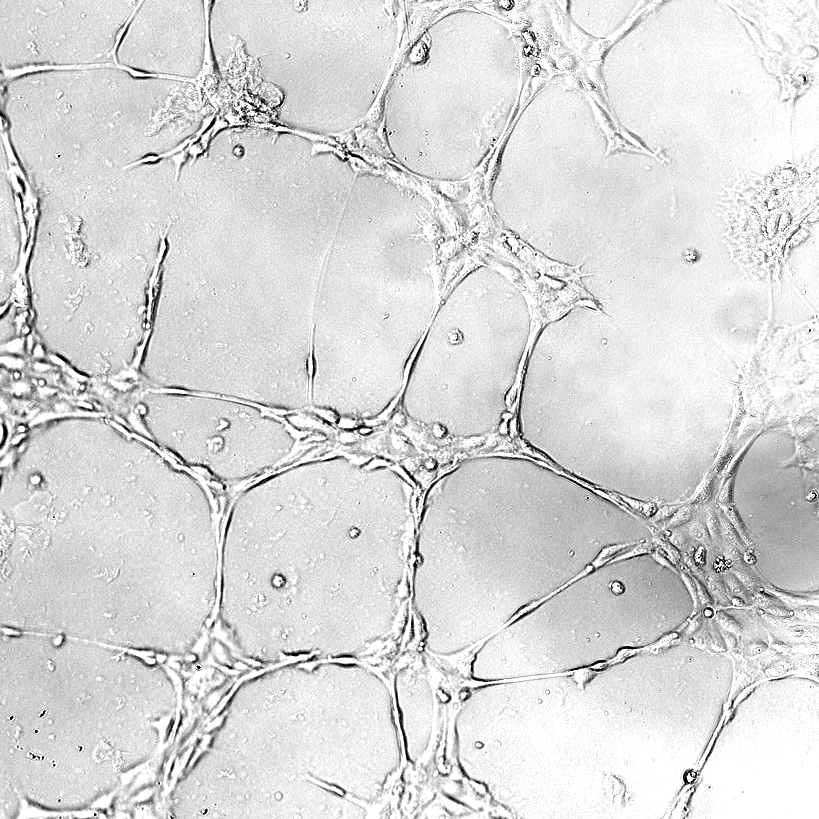

Supplement: Supplementary file 11 — Source Data for Figure 6 [file EMMM-15-e16373-s010.zip › Figure 6/6J.K/conDM.tif]

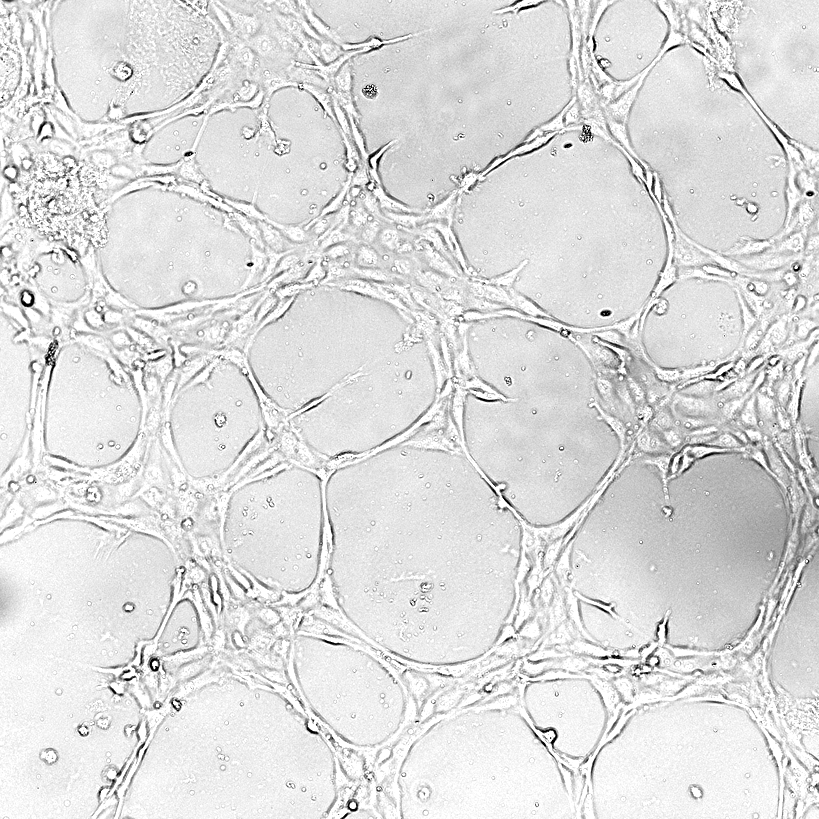

Supplement: Supplementary file 11 — Source Data for Figure 6 [file EMMM-15-e16373-s010.zip › Figure 6/6J.K/conPG.tif]

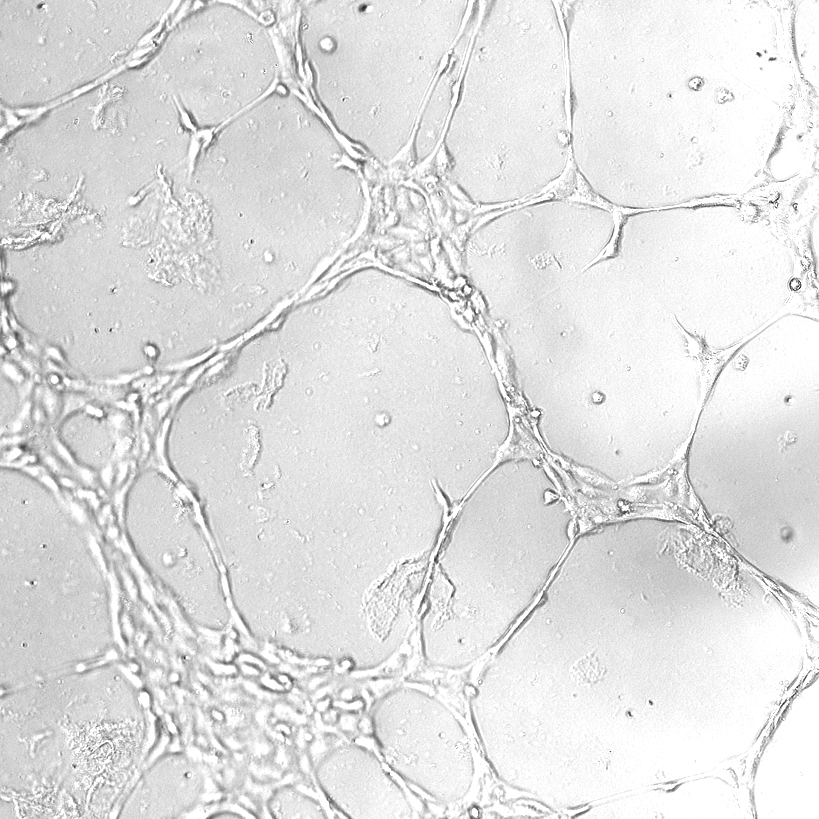

Supplement: Supplementary file 11 — Source Data for Figure 6 [file EMMM-15-e16373-s010.zip › Figure 6/6J.K/lentiDM.tif]

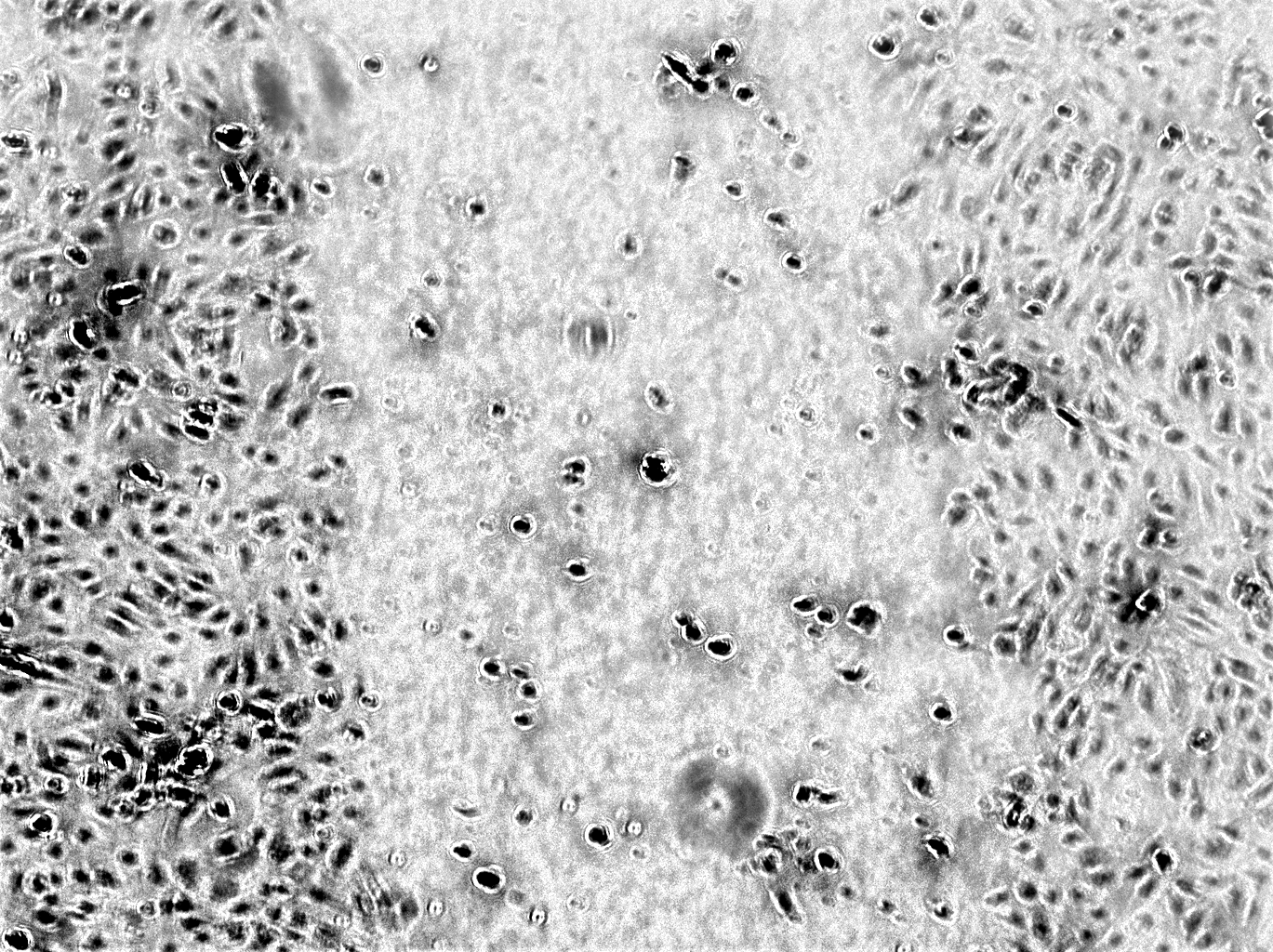

Supplement: Supplementary file 11 — Source Data for Figure 6 [file EMMM-15-e16373-s010.zip › Figure 6/6G.H/DMSO-shFOS.tif]

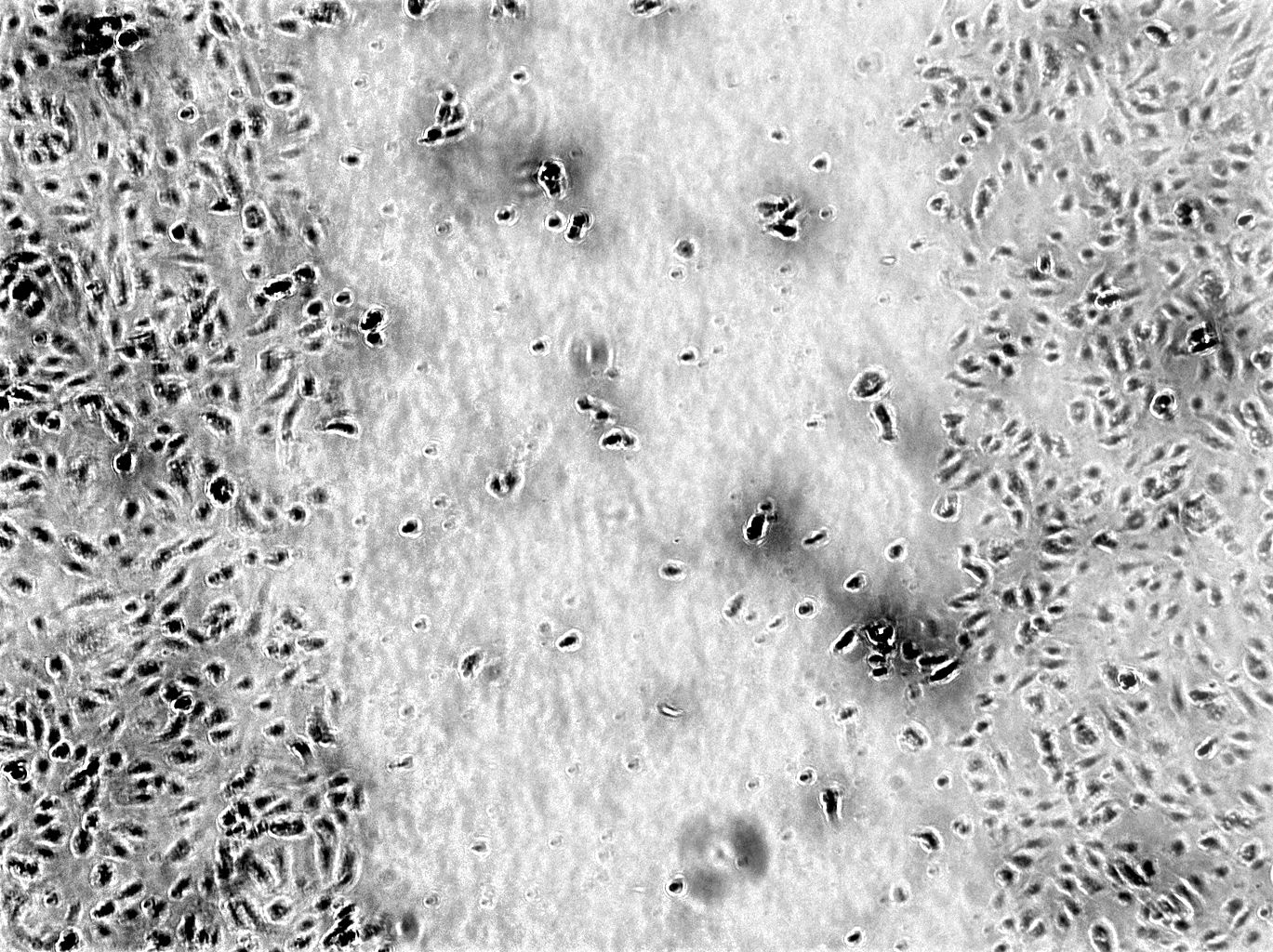

Supplement: Supplementary file 11 — Source Data for Figure 6 [file EMMM-15-e16373-s010.zip › Figure 6/6G.H/PGF-shFOS.tif]

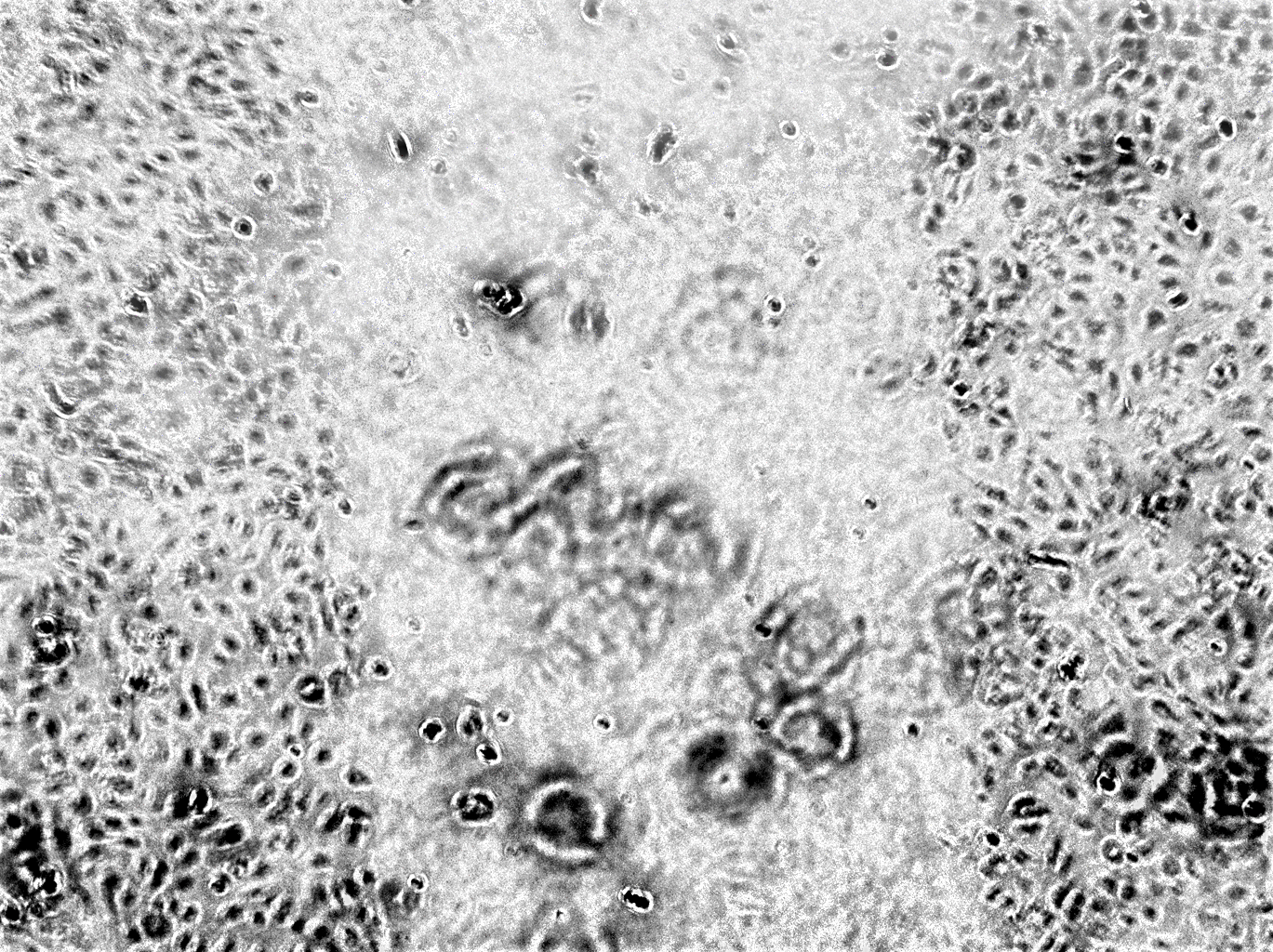

Supplement: Supplementary file 11 — Source Data for Figure 6 [file EMMM-15-e16373-s010.zip › Figure 6/6G.H/DMSO-shSCR.tif]

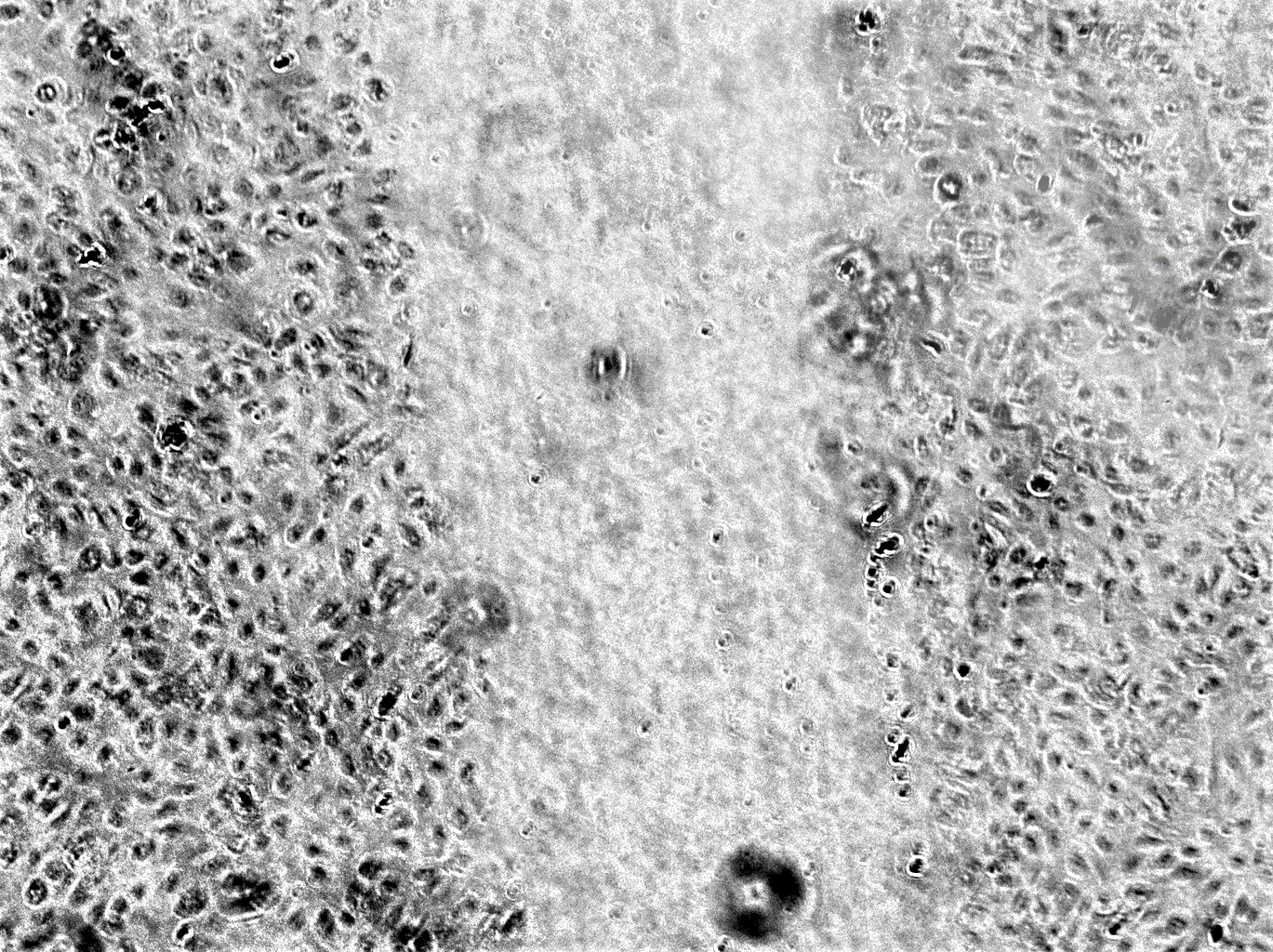

Supplement: Supplementary file 11 — Source Data for Figure 6 [file EMMM-15-e16373-s010.zip › Figure 6/6G.H/PGF-shSCR.tif]

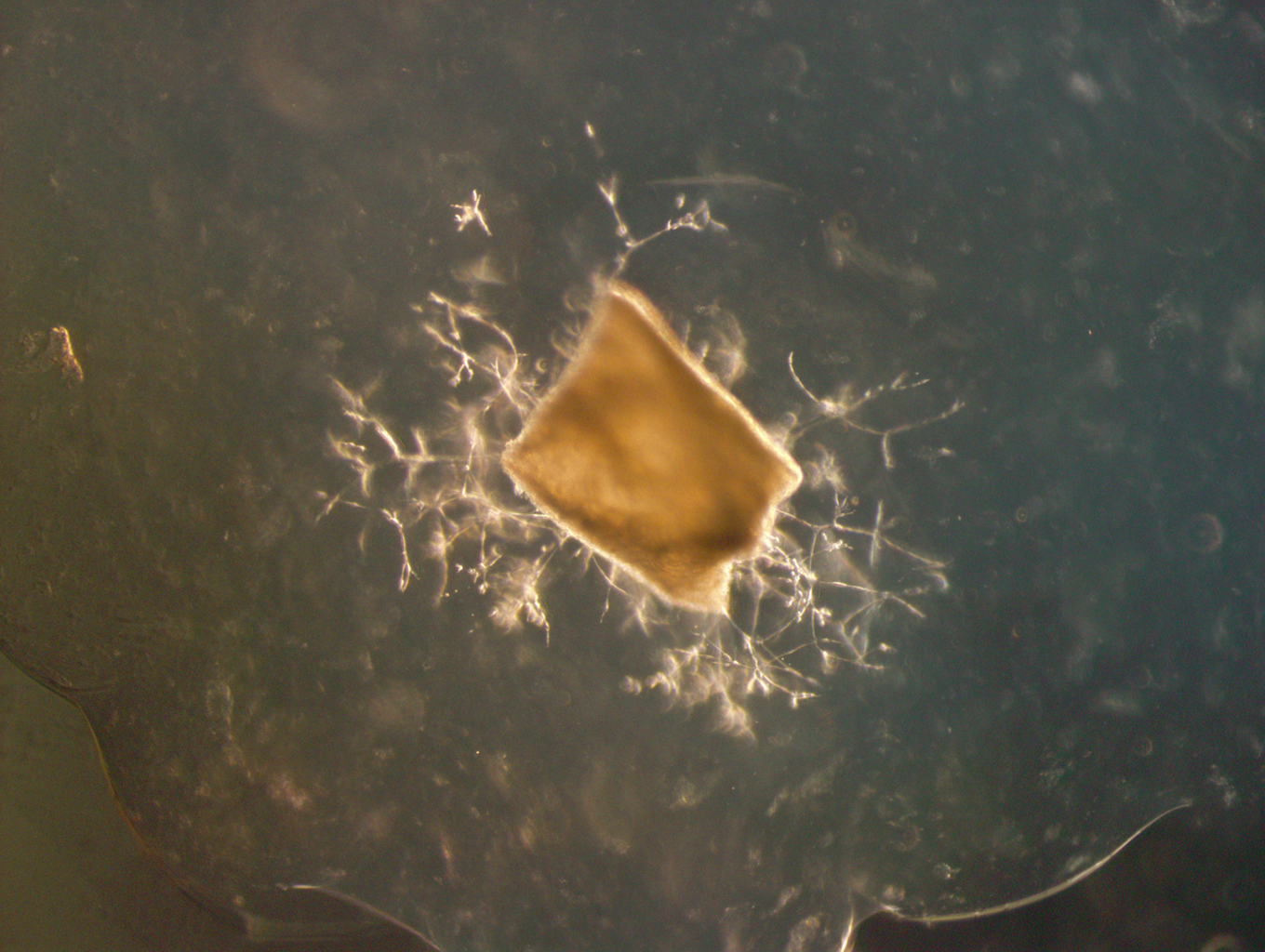

Supplement: Supplementary file 11 — Source Data for Figure 6 [file EMMM-15-e16373-s010.zip › Figure 6/6N.O/DMSO-DMSO.tif]

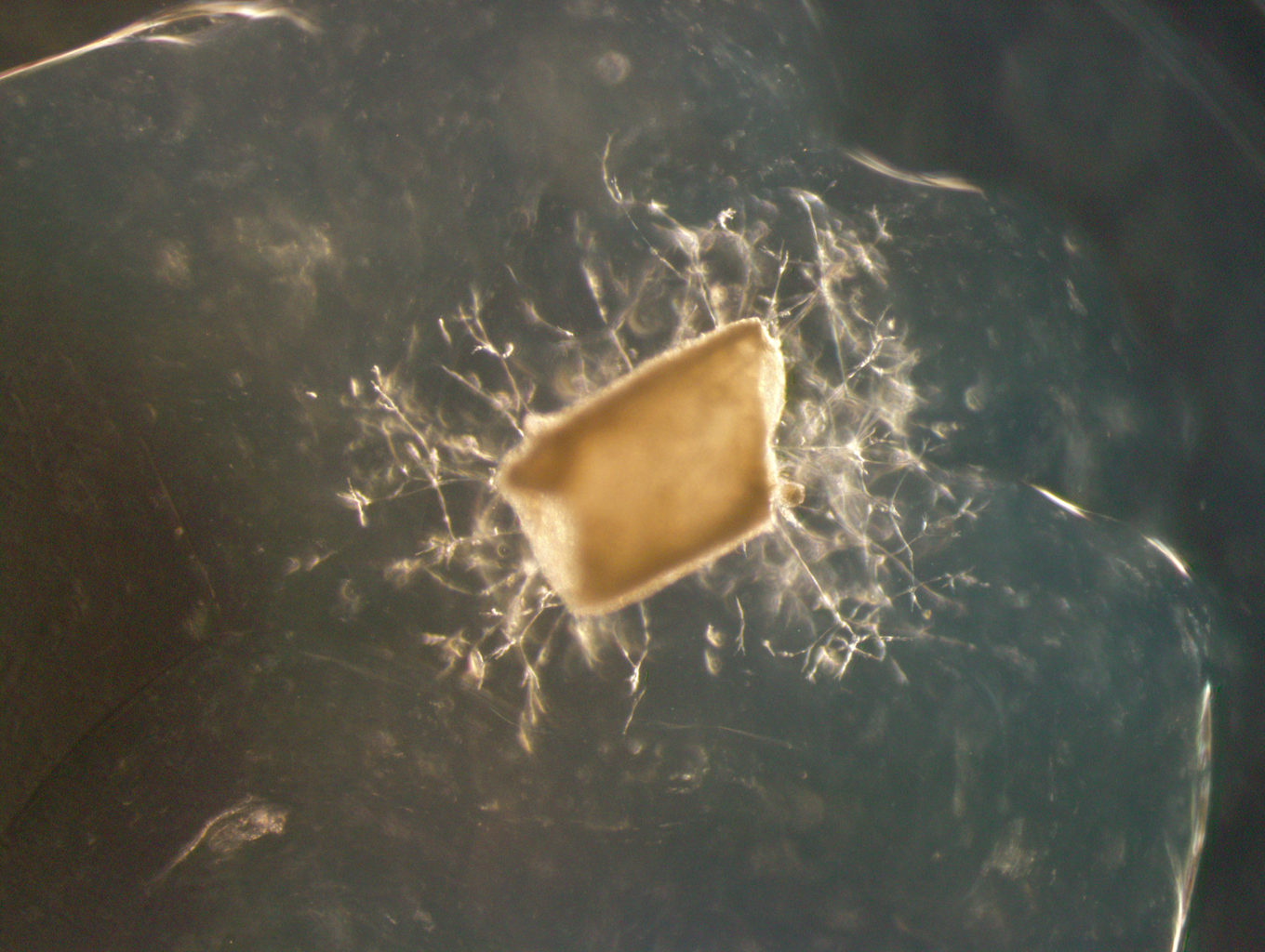

Supplement: Supplementary file 11 — Source Data for Figure 6 [file EMMM-15-e16373-s010.zip › Figure 6/6N.O/DMSO-SR11302.tif]

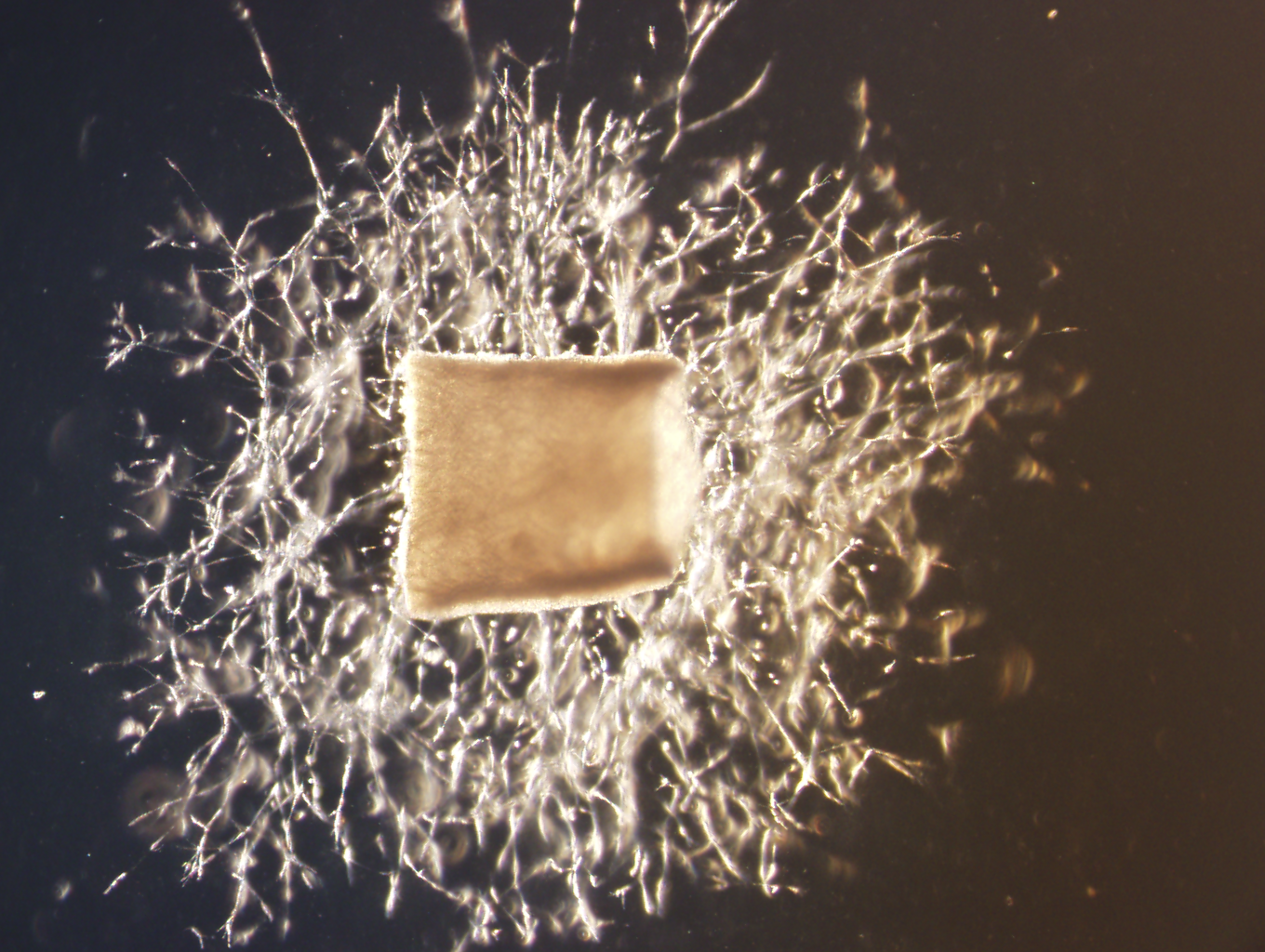

Supplement: Supplementary file 11 — Source Data for Figure 6 [file EMMM-15-e16373-s010.zip › Figure 6/6N.O/DMSO-PGF.tif]

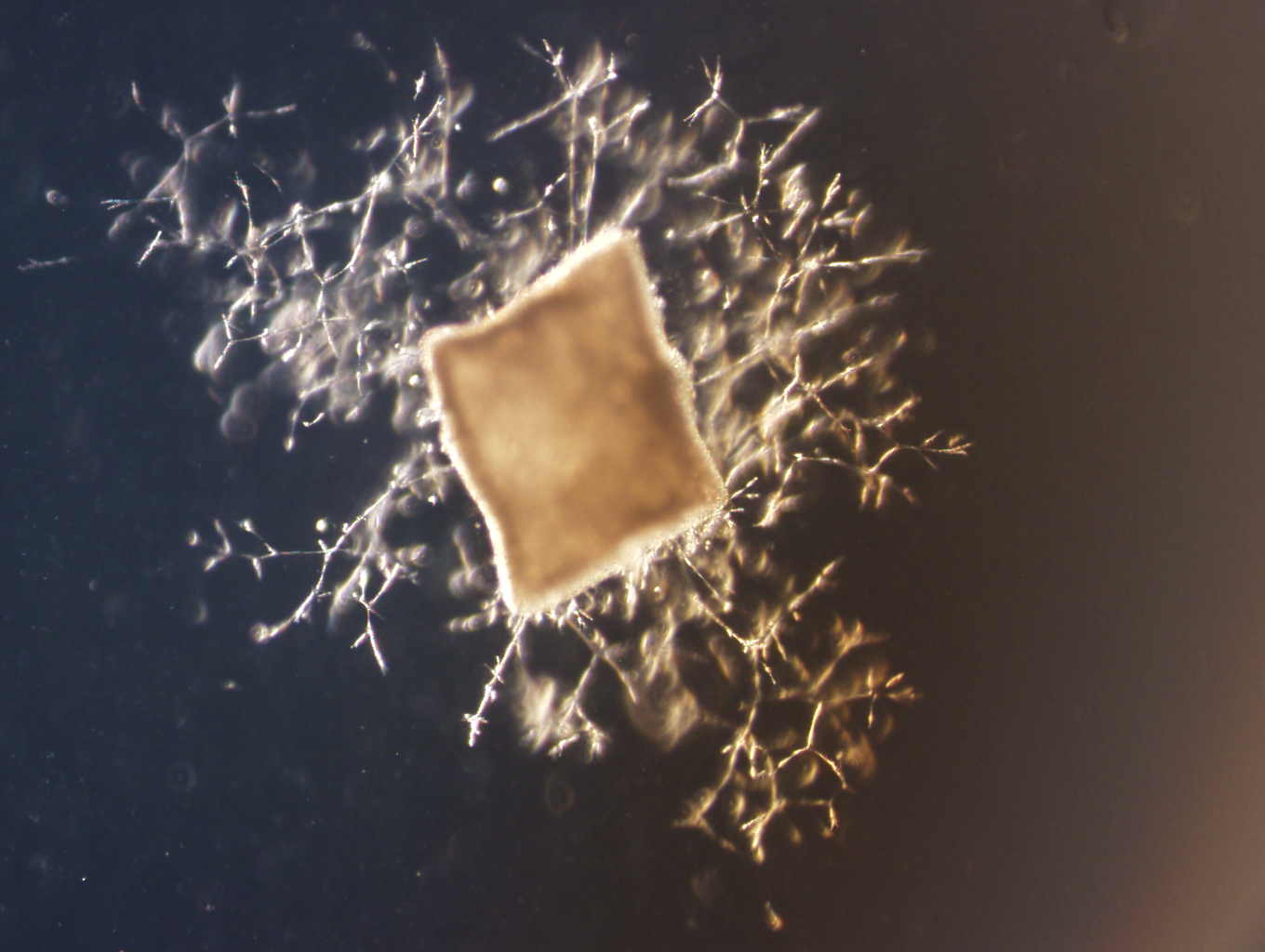

Supplement: Supplementary file 11 — Source Data for Figure 6 [file EMMM-15-e16373-s010.zip › Figure 6/6N.O/PGF-SR11302.tif]

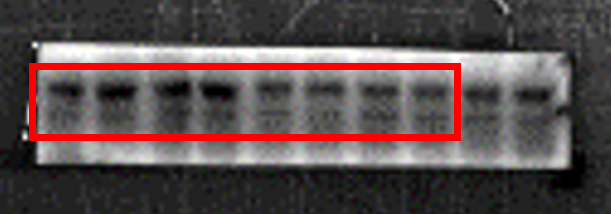

Supplement: Supplementary file 12 — Source Data for Figure 7 [file EMMM-15-e16373-s001.zip › Figure 7/G/CAMK.tif]

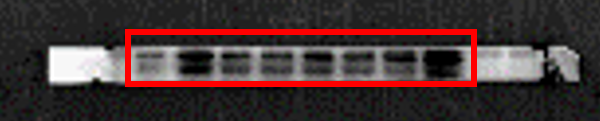

Supplement: Supplementary file 12 — Source Data for Figure 7 [file EMMM-15-e16373-s001.zip › Figure 7/G/pCAMK.tif]

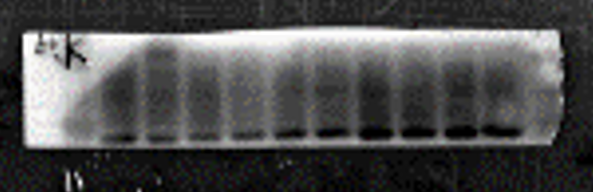

Supplement: Supplementary file 12 — Source Data for Figure 7 [file EMMM-15-e16373-s001.zip › Figure 7/M/ELK-1.tif]

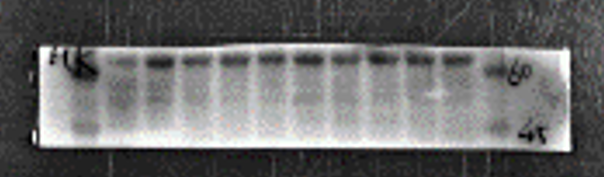

Supplement: Supplementary file 12 — Source Data for Figure 7 [file EMMM-15-e16373-s001.zip › Figure 7/M/Pelk-1.tif]

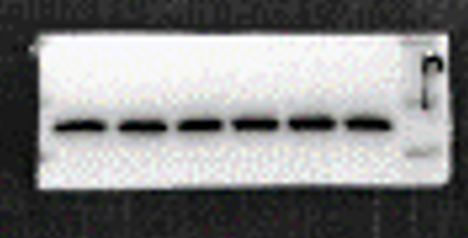

Supplement: Supplementary file 12 — Source Data for Figure 7 [file EMMM-15-e16373-s001.zip › Figure 7/J/P38.tif]

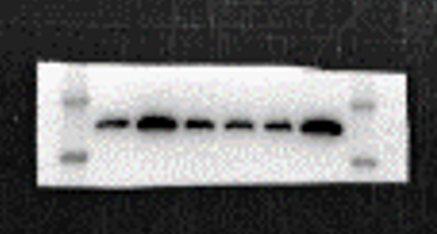

Supplement: Supplementary file 12 — Source Data for Figure 7 [file EMMM-15-e16373-s001.zip › Figure 7/J/P-P38.tif]

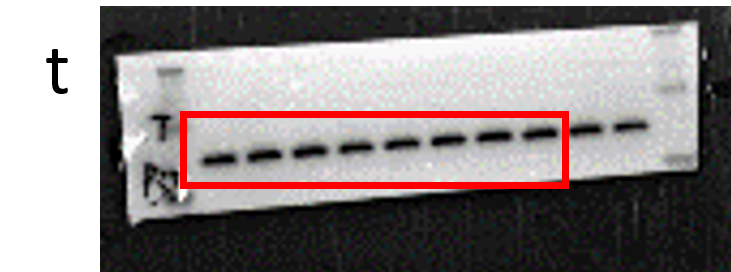

Supplement: Supplementary file 12 — Source Data for Figure 7 [file EMMM-15-e16373-s001.zip › Figure 7/E/t-p38.png]

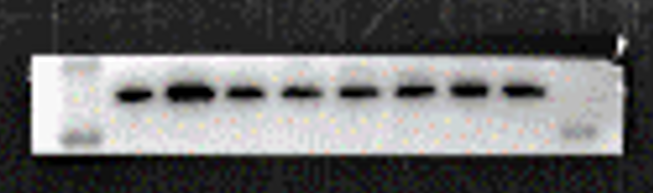

Supplement: Supplementary file 12 — Source Data for Figure 7 [file EMMM-15-e16373-s001.zip › Figure 7/E/p-p38.tif]

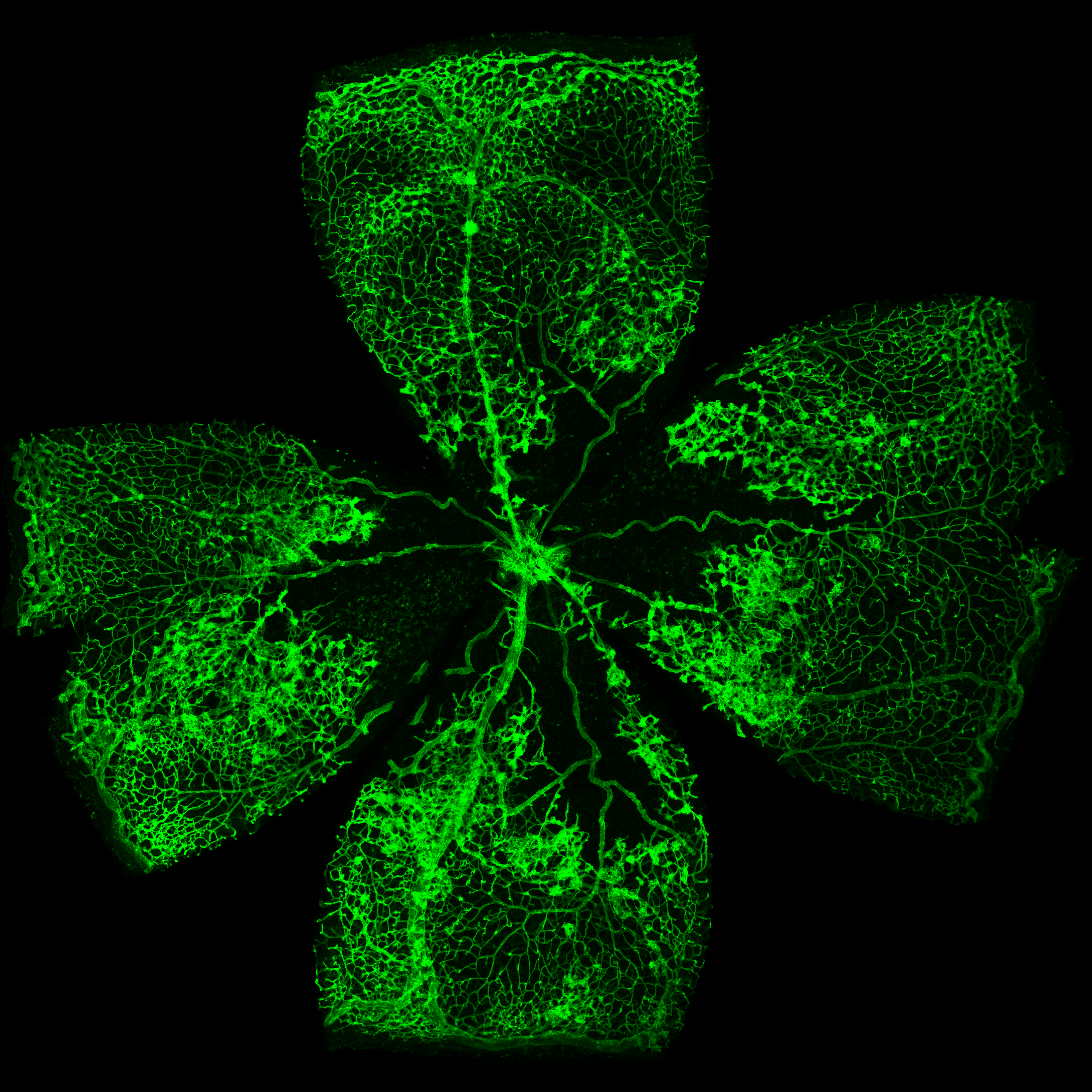

Supplement: Supplementary file 13 — Source Data for Figure 8 [file EMMM-15-e16373-s008.zip › Figure 8/WT-PBS.tif]

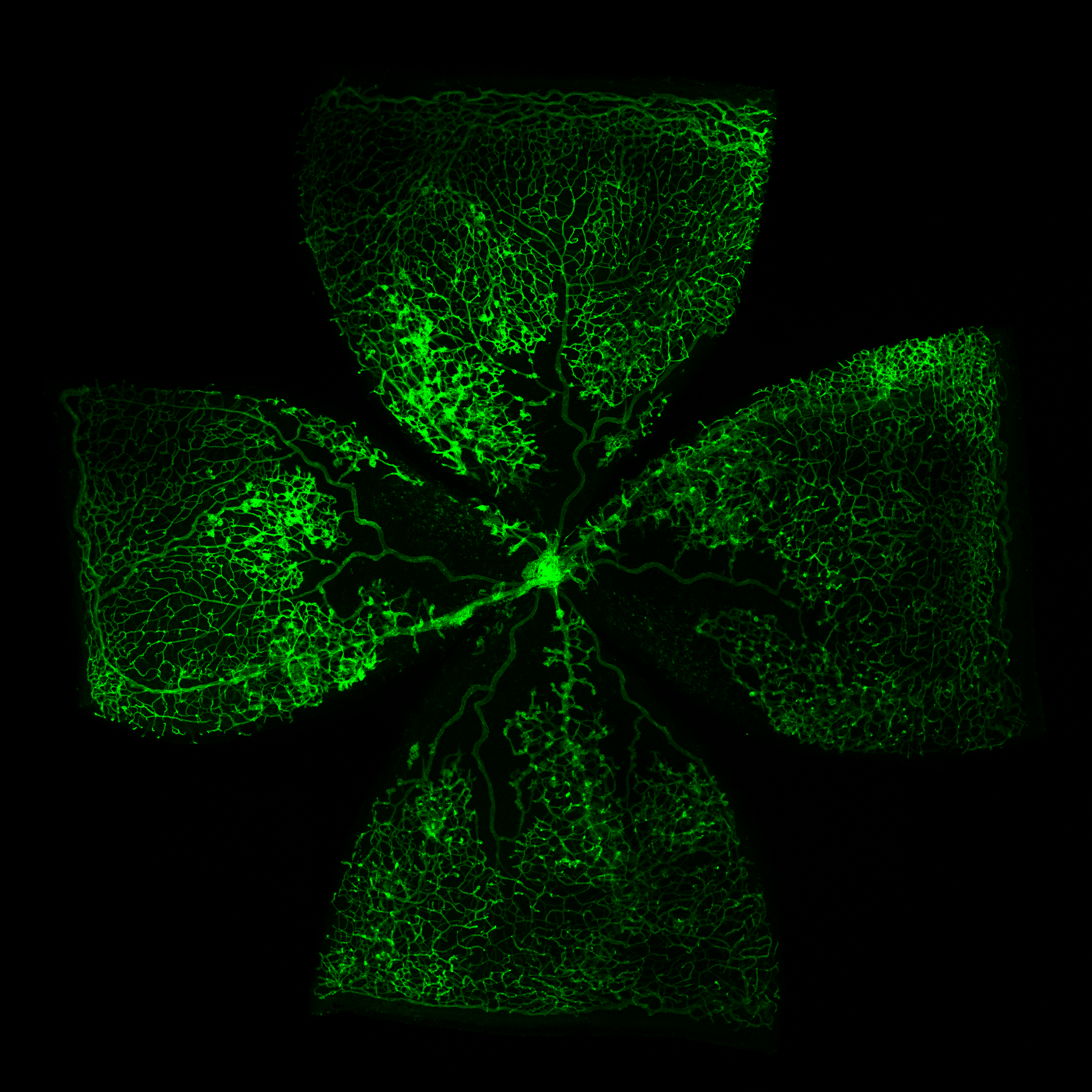

Supplement: Supplementary file 13 — Source Data for Figure 8 [file EMMM-15-e16373-s008.zip › Figure 8/WT-AL8810.tif]

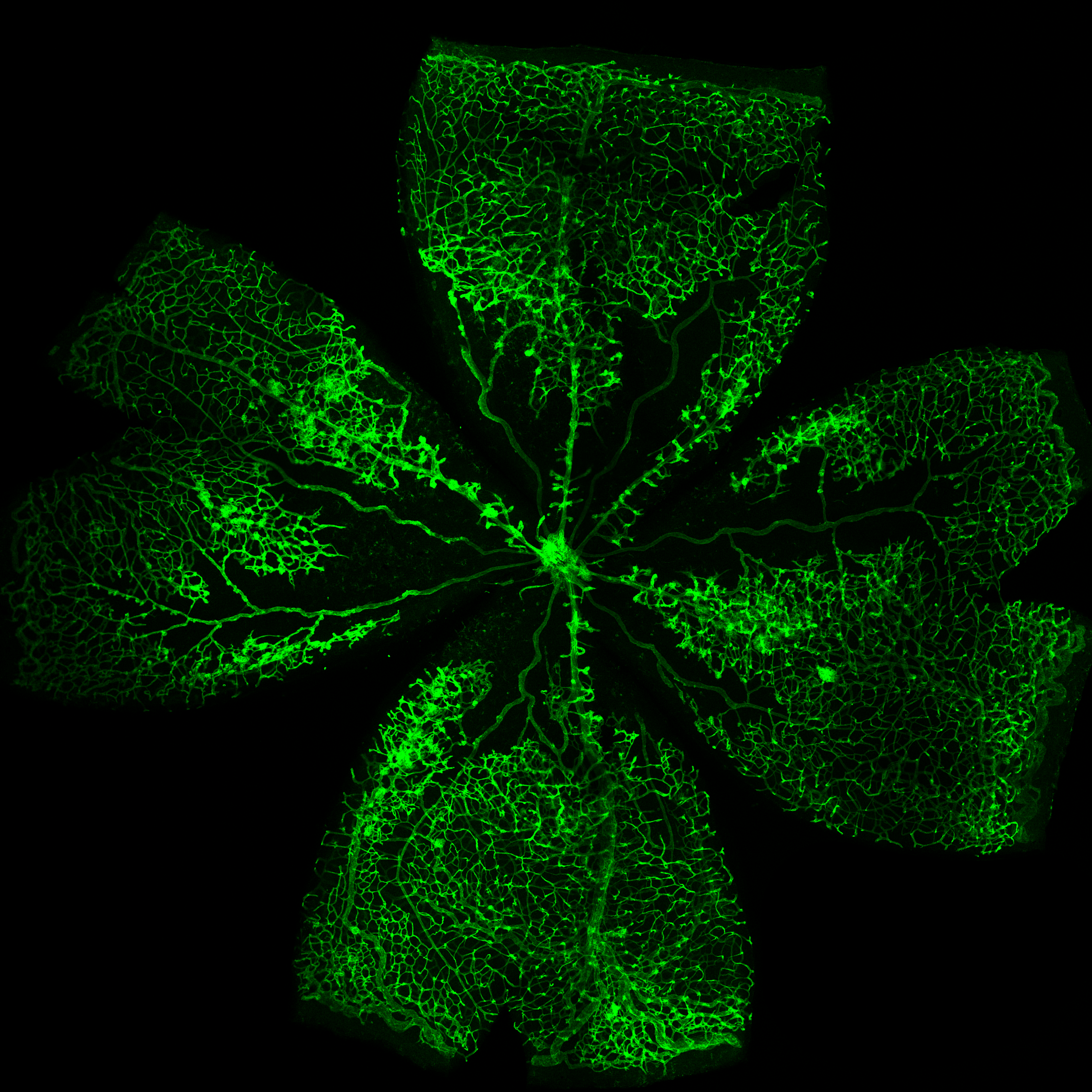

Supplement: Supplementary file 13 — Source Data for Figure 8 [file EMMM-15-e16373-s008.zip › Figure 8/Cxcr2-PBS.tif]

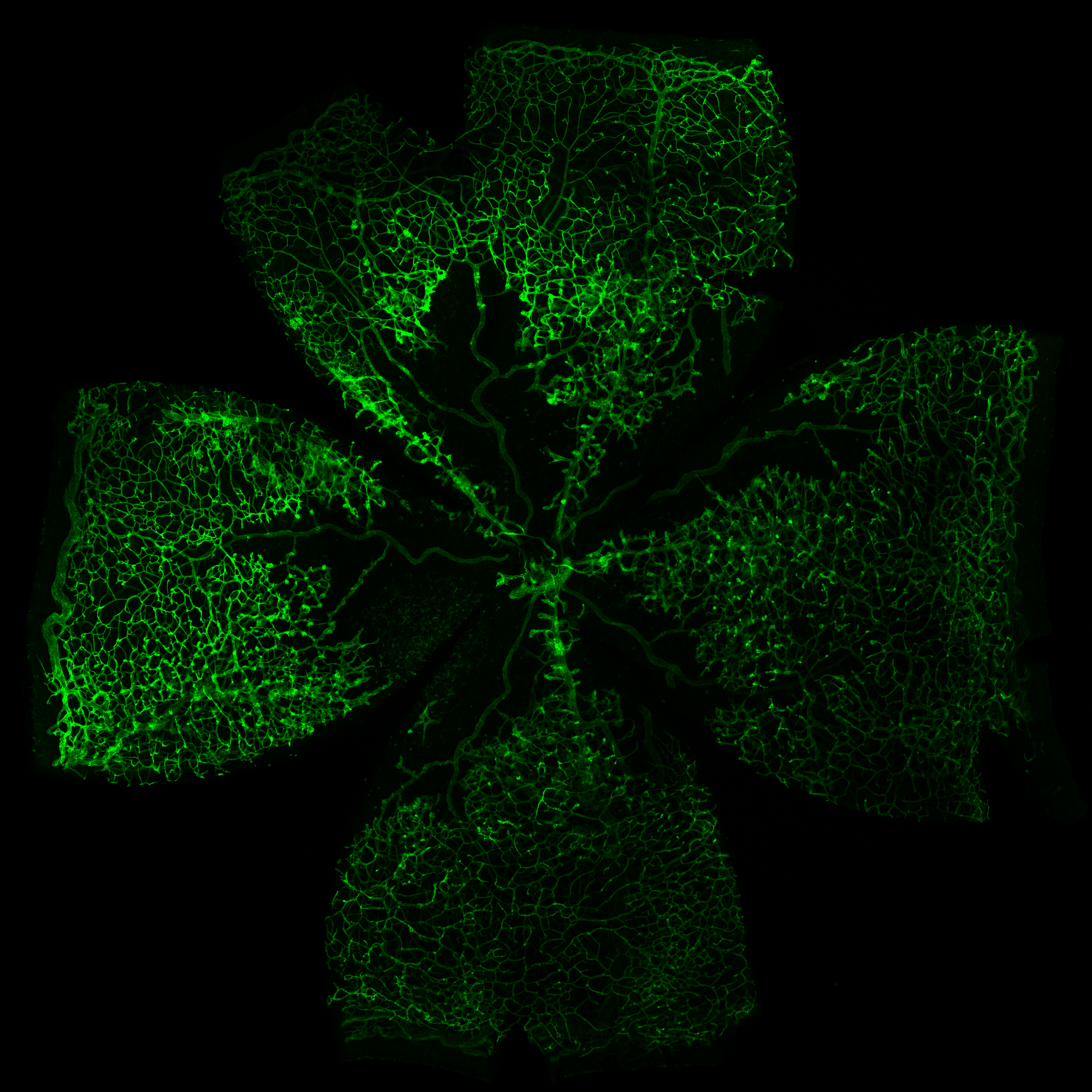

Supplement: Supplementary file 13 — Source Data for Figure 8 [file EMMM-15-e16373-s008.zip › Figure 8/Cxcr2-AL8810.tif]
