# Supplementary material for: PGF2α facilitates pathological retinal angiogenesis by modulating endothelial FOS‐driven ELR + CXC chemokine expression
Source: EMBO Mol Med. 2022 Dec 13;15(1):e16373. doi: 10.15252/emmm.202216373 (PMC9832840; doi:10.15252/emmm.202216373)
Supplement: Supplementary file 14 [file EMMM-15-e16373-s003.pdf]

EMBO Molecular Medicine

PGF2α facilitates pathological retinal angiogenesis by modulating endothelial FOS-driven ELR+ CXC chemokine expression

Ying Yu, Yan Zhao, Yi Lei, Huying Ning, Yaqiang Zhang, Guilin Chen, Chenchen Wang, Qiangyou Wan, Shumin Guo, Qian Liu, Ruotian Xie, Yujuan Zhuo, Shuai Yan, Jing Zhao, Fengjiang Wei, Lu Wang, Xiaohong Wang, Weidong Li, and Hua Yan
DOI: 10.15252/emmm.202216373

Corresponding authors: Ying Yu (yuying@tmu.edu.cn) , Hua Yan (zyyyanhua@tmu.edu.cn)

Review Timeline:

Submission Date:	26th May 22
Editorial Decision:	20th Jun 22
Revision Received:	16th Oct 22
Editorial Decision:	2nd Nov 22
Revision Received:	11th Nov 22
Accepted:	15th Nov 22

Editor: Zeljko Durdevic

Transaction Report:

(Note: With the exception of the correction of typographical or spelling errors that could be a source of ambiguity, letters and reports are not edited. Depending on transfer agreements, referee reports obtained elsewhere may or may not be included in this compilation. Referee reports are anonymous unless the Referee chooses to sign their reports.)

20th Jun 2022

Dear Prof. Yu,

Thank you for the submission of your manuscript to EMBO Molecular Medicine. We have now received feedback from the three reviewers who agreed to evaluate your manuscript. As you will see from the reports below, the referees acknowledge the interest of the study but also raise important concerns that should be addressed in a major revision. Please make sure to pay particular attention to the grammar and syntax and I would recommend running the article by a native English speaker.

Further consideration of a revision that addresses reviewers' concerns in full will entail a second round of review. EMBO Molecular Medicine encourages a single round of revision only and therefore, acceptance or rejection of the manuscript will depend on the completeness of your responses included in the next, final version of the manuscript. For this reason, and to save you from any frustrations in the end, I would strongly advise against returning an incomplete revision.

We would welcome the submission of a revised version within three months for further consideration. Please let us know if you require longer to complete the revision.

Please use this link to login to the manuscript system and submit your revision: https://embomolmed.msubmit.net/cgi-bin/main.plex

I look forward to receiving your revised manuscript.

Yours sincerely,

Zeljko Durdevic

Zeljko Durdevic
Editor
EMBO Molecular Medicine

***** Reviewer's comments *****

Referee #1 (Comments on Novelty/Model System for Author):

The models used are adequate.

Referee #1 (Remarks for Author):

In this manuscript, the authors observed that serum PGF2α was increased in PDR patients, and PGF2α receptor (Ptgfr) was upregulated in retinal endothelial cells of mouse model of OIR. By using endothelial cell-specific Ptgfr knockout mice (Tie2-cre and VE-Cadherin-cre), they further demonstrated that deletion of PGF2α receptor in endothelial cells reduced neovascularization in OIR mice. In addition, they administered two different PGF2α receptor inhibitors to the mice, showed that both of the inhibitors were able to attenuate neovascularization in OIR mice. In cultured HRMECs, OIR mice as well as PDR patients, the authors also investigated potential pathways involved---including activation of PGF2a/PTGFR axis, secretion of ELR+ CXC chemokines and FOS-mediated transcriptional regulation. They concluded that activation of PGF2a/PTGFR axis accelerates pathological retinal angiogenesis, through ELR+ CXC -FOS regulation, independent of VEGF.
In general, the study was well designed and the manuscript was well written. The results presented support the conclusion.

Major concerns:

1. For knock out efficiency, the authors showed that Ptgfr mRNA levels were decreased in the retinal ECs of CKO-T (Figure 2B) and CKO-V (Figure EV4B) mice. Have the authors ever looked at the protein levels of PTGFR in those mice?
2. The authors showed that PTGFR deletion in retinal ECs reduced the expression of the chemokines Cxcl1, Cxcl2, Cxcl3, and Cxcl5 in retinas from OIR mice on postnatal day 16(Figure 5D). Have the authors ever measured the expression levels of VEGF and its receptors in those mice?

Referee #2 (Comments on Novelty/Model System for Author):

Overall, the study brings new understanding to the role of PTGFR signaling in pathological angiogenesis. Novelty is ranked as medium, as other studies have previously reported the role of PTGFR in pathological angiogenesis, albeit not in these specific models. The investigation of possible mechanisms of action adds to the novelty however. Models are adequate for the most part, although the authors should be careful about comparing proliferative diabetic retinopathy and mouse oxygen-induced retinopathy, which have different aetiologies. I have given suggestions about some of the models in my general comments.

Referee #2 (Remarks for Author):

This study by Zhao and colleagues aims to evaluate the role of the PGF2α receptor (Ptgfr) in pathological retinal neovascularization. They show that Ptgfr is upregulated in retinal endothelial cells during oxygen-induced retinopathy and that its disruption in endothelial cells attenuates pathological neovascularization in this model. Mechanistically, they show that PGF2α promotes the proliferation and tube formation of human retinal microvascular endothelial cells through the release of ELR+ CXC chemokines, such as CXCL8 and CXCL2. They also demonstrate that the Gq/CAMK2G/p38/ELK-1/FOS pathway is required for the expression of these chemokines by PGF2α. Upregulated FOS-mediated ELR+ CXC chemokine expression was also observed in retinal ECs from patients with proliferative diabetic retinopathy. Finally, treatment with a PTGFR inhibitor decreased pathological angiogenesis during OIR in a CXCR2-dependent manner. This leads them to claim that inhibition of PTGFR could represent a new avenue for the treatment of pathological retinal neovascularization.
This is an interesting study in which the authors demonstrate a role for the PGF2α/PTGFR signaling axis in proliferative

retinopathy. Overall, the study is well-designed. However, the following issues should be addressed:

Major

- The etiology of diabetic proliferative retinopathy and the mouse model of oxygen-induced retinopathy is vastly different. While I appreciate that the PGF2α/PTGFR signaling axis may be implicated in pathological angiogenesis in both diseases, it is likely that the trigger for increased PGF2α/PTGFR between both pathologies is distinct. This should be addressed in the discussion. If available, public databases from microarrays originating from whole-genome expression in the first month of life in groups of infants with and without ROP could be analysed to assess PTGFR signaling in ROP (accession number GSE32472).
- For lentiviral overexpression of CXCL1, demonstrate expression of the protein: which cell types are infected, and where is CXCl1 expression localized? Why is overexpression not significant in control mice? If expression of CXCL1 lentivirally driven by the EF1α, it would still be expected to have overexpression even in control animals?
- Fos transcriptional activities are also regulated by phosphorylation as a result of the activation of intracellular signaling cascades. As such, the status of FOS phosphorylation should also be assessed in response to changes in PTGFR signaling.
- Authors claim that PTGFR signaling does not affect VEGF expression. However, previous studies have shown that PTGFR can induce VEGF expression; how do authors reconcile these data with their own studies (DOI: 10.1016/j.anireprosci.2018.10.003)?
- The effects of PGF2α on cell cycle (BrdU incorporation) should be performed to complement proliferation assays.

Minor

- There are a few grammatical errors. Proof-read the document.
- Some methods are lacking in the document: for example, protocol for magnetic cells sorting is missing
- I would be careful with the statement that PGF2α induces 'tube' formation with the presented assay; given that treatments are only performed for 6 hours, it is unlikely that tubulogenesis occurs within this timeframe and what is observed is more likely to be cord formation. What happens to these structures after 24h?

Referee #3 (Comments on Novelty/Model System for Author):

1. Technical Quality. Study is very well controlled, and thorough with regard to the range of analyses undertaken.
2. Novelty. I am unaware of any previous studies reporting the involvement of PGF2a in retinal vascular disease, and the authors have done an excellent job in unraveling the signaling mechanisms involved.
3. Medical impact. Identifying the PGF2a axis as a driver of pathological retinal neovascularisation is demonstrated in in vitro models and in the OIR mouse model in this study but not proven in DR. The authors note that they would need to measure PGF2a levels in vitreous from patients with DR, plus some further analysis of patient tissue would be helpful. Also, their observations indicate that the PGF2a axis operates independently of the well known VEGF driven pathway of pathological angiogenesis in DR. Yet many patients who receive anti-VEGFs as therapy show marked benefit. So how much is the PGF2a pathway really contributing?
4. Adequacy of model system: The only mouse model used is the OIR model, in which pathological neovascularisation is induced in the developing postnatal mouse retina by hypoxia. This has little in common with the pathology induced by diabetes, and there is no reference in this study to diabetic mouse models. Moreover, hypoxia drives VEGF expression via HIF1a, which again is not mentioned.

Referee #3 (Remarks for Author):

This is an interesting and well constructed study that merits publication after attention to a few minor points.
1. The study has clinical relevance because of the links with DR, but the only in vivo model used is the OIR postnatal mouse, which is not a model of DR and differs in several respects. In particular, pathology is driven by hypoxia and VEGF induction via HIF1a. Given that VEGF blockers work well in the OIR model, and also in many patients with DR, the authors need to provide some points of discussion to explain why they think targeting PGF2a therapeutically might work, as presumably the VEGF axis would continue to drive pathology.
2. Line 130 - Cdh5 expression does not provide a measure of purity of the endothelial cells. A similar result would surely be observed in the presence of other cell types such as pericytes.
3. The legends to figures EV2, 3, 7 and 8 would benefit from a few lines of explanatory text.
4. Some editorial input on the finished MS might be necessary to improve the written English in places.

Reviewer #1:

In general, the study was well designed and the manuscript was well written. The results presented support the conclusion.

We would like to thank the Reviewer for favorable comments.

Major concerns:

1. For knock out efficiency, the authors showed that Ptgfr mRNA levels were decreased in the retinal ECs of CKO-T (Figure 2B) and CKO-V (Figure EV3B) mice. Have the authors ever looked at the protein levels of PTGFR in those mice?

We totally agree. It would be perfect to examine PTGFR protein expression in the CKO mice. However, there is no reliable PTGFR antibody available for immunohistochemistry and Western blot for mouse tissues, as we tested previously *(Arterioscler Thromb Vasc Biol. 2015;35:1687-95; Diabetes. 2018;67:1748-1760)*

2. The authors showed that PTGFR deletion in retinal ECs reduced the expression of the chemokines Cxcl1, Cxcl2, Cxcl3, and Cxcl5 in retinas from OIR mice on postnatal day 16(Figure 5D). Have the authors ever measured the expression levels of VEGF and its receptors in those mice?

This is a very good question. We did analyze the expression of VEGFA and its receptors in $PGF_{2\alpha}$-treated HRMECs via RNA-seq and RT-RTPCR, and failed to detect significant alterations of VEGFA and its receptors in $PGF_{2\alpha}$-treated HRMECs; And we mentioned in the results; please see **Figure 4A, Appendix Figure S2A-B, Page 9, lines 184-186.**

As suggested, we also compared the expression levels of VEGFA and its receptors between CKO mice and littermate controls. Again, PTGFR deficiency did not dramatically influence retinal expression of VEGFA and its receptors in OIR mice (**Appendix Figure S2C-S2D**). We amended the results; please see **Page 9, lines 184-186.**

Reviewer #2

This is an interesting study in which the authors demonstrate a role for the PGF2α/PTGFR signaling axis in proliferative retinopathy. Overall, the study is well-designed.

We thank the reviewer for the favorable comments.

Major

1. The etiology of diabetic proliferative retinopathy and the mouse model of oxygen-induced retinopathy is vastly different. While I appreciate that the PGF2α/PTGFR signaling axis may be implicated in pathological angiogenesis in both diseases, it is likely that the trigger for increased PGF2α/PTGFR between both pathologies is distinct. This should be addressed in the discussion. If available, public databases from microarrays originating from whole-genome expression in the first month of life in groups of infants with and without ROP could be analyzed to assess PTGFR signaling in ROP (accession number GSE32472).

We agree the reviewer's opinion. Oxygen-induced retinopathy (OIR) is not a perfect animal model for proliferative diabetic retinopathy (PDR), because of no metabolite disorders involved in. Since high oxygen can induce pathological alternations of retina blood vessel as PDR, and OIR model is routinely used to study PDR (*Nat Protoc* 2009; 4:1565; *J Exp Med* 2017; 214:1029; *Proc Natl Acad Sci U S A* 2015; 112:12163; *Proc Natl Acad Sci U S A* 2015;112:10401). It is possible that the intrinsic microenveroment to activate $PGF_{2\alpha}$/PTGFR axis in PDR is quite different from that in OIR. As suggested, we amended the suggestion, please see **Page 16, Lines 344-347.**

As suggested, we analyzed PTGFR-mediated signaling in blood leukocytes from preterm infants (data from **peripheral blood leukocytes**, GSE32472; *PLoS One* 2013; 8: e78585). Not like in retinas in PDR patients (Figure 4D-G and Figure 6B), the induction of $PGF_{2\alpha}$/ PTGFR axis -mediated signaling is not detected in peripheral blood leukocytes from

preterm infants, probably because PTGFR is predominately expressed in ECs, not inflammatory cells (**Figure 1F-G**), As such, we expanded the discussion; please see **Page 17; Lines 355-360**.

2. For lentiviral overexpression of CXCL1, demonstrate expression of the protein: which cell types are infected, and where is CXCl1 expression localized? Why is overexpression not significant in control mice? If expression of CXCL1 lentivirally driven by the EF1α, it would still be expected to have overexpression even in control animals?

EF1α is a constitutive promoter of human origin that is widely used to drive ectopic gene expression in various tissues (*Anticancer Res. 2002; 22(6A):3325-30)*, and the pLenti-GIII-EF1α vector was supplied by ABM Lentivectors (Cat #, LV205018). We examined the CXCL1 expression in retina tissues from Lenti-GIII-EF1α-*Cxcl1* (Lenti- *Cxcl1*) virus-injected mice, and observed an increase trend (p=0.285) of CXCL1 protein in Lenti- *Cxcl1* virus-infected WT retinas, despite robust induction of *Cxcl1* mRNA expression **(Reviewer Figure 1).** The discrepancy is probably due to high level of *CXCL1* expression in retinas induced by oxygen in OIR WT mice (**Figure 5C-D**). As such, we amended the statistical value in the Figure (**new Figure 5 G**).

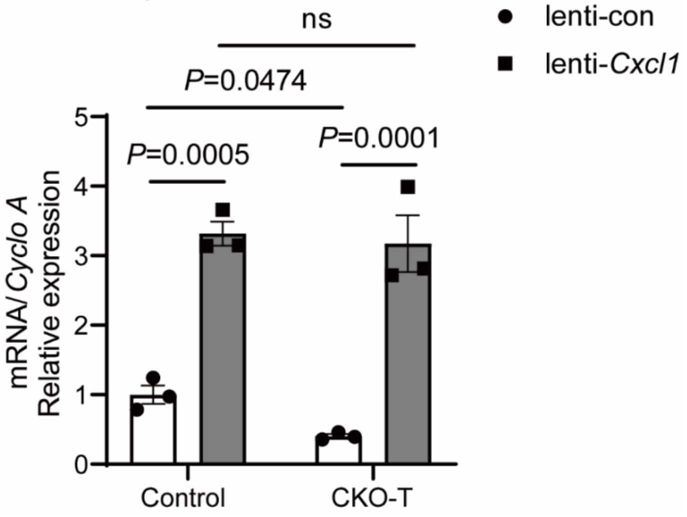

Reviewer Figure 1: Retinal *Cxcl1* mRNA expression levels in *Cxcl1*-expressing lentivirus-injected CKO mice. Quantitation of retinal *Cxcl1* mRNA expression (n=3). n.s. stands for "not significant." Data were analyzed by two-way ANOVA with Tukey's multiple comparisons test.

3.Fos transcriptional activities are also regulated by phosphorylation as a result of the activation of intracellular signaling cascades. As such, the status of FOS phosphorylation should also be assessed in response to changes in PTGFR signaling.

This is a good question. Phosphorylated T232 of Fos is associated with the transcriptional activity of translocation of nucleus (*Nature* 1994; 371: 171-175). As suggested, we analyzed the Fos phosphorylation levels in $PGF_{2\alpha}$-treated HRMECs; and observed increased of phosphorylated and total Fos protein in $PGF_{2\alpha}$-treated HRMECs; please see **Appendix Figure S4B-D**.

4.Authors claim that PTGFR signaling does not affect VEGF expression. However, previous studies have shown that PTGFR can induce VEGF expression; how do authors reconcile these data with their own studies (DOI: 10.1016/j.anireprosci.2018.10.003)?

Thanks for the concern. We failed to detect notable changes of VEGF expression in $PGF_{2\alpha}$-treated HRMECs using both RNA-seq and RT-PCR assay (**Figure 4A; Appendix Figure S2A-B**), and in $PTGFR^{-/-}$ retinas (**Appendix Figure S2C-D**). In bovine endometrial epithelial cells, however, PTGFR activation increases VEGF expression (*Anim Reprod Sci* 2018; 199: 30-39). These observations suggest PTGFR may mediate different signaling in different type cells. As requested, we expanded the discussion and cited the reference; see **Page 19, Lines 412-415.**

5. The effects of PGF2α on cell cycle (BrdU incorporation) should be performed to complement proliferation assays.

As requested, we amended, please see **Figure 3D and 3E**, **Page 8, Lines 163-164**

Minor

- There are a few grammatical errors. Proof-read the document.

Thank you. The manuscript was edited by professional agent (www. Editage.com).

- Some methods are lacking in the document: for example, protocol for magnetic cells sorting is missing.

We amended. Please see Method section **(Page 29, Paragraph 3)**.

- I would be careful with the statement that PGF2α induces 'tube' formation with the presented assay; given that treatments are only performed for 6 hours, it is unlikely that tubulogenesis occurs within this timeframe and what is observed is more likely to be cord formation. What happens to these structures after 24h?

As suggested, we repeated the tube formation assay by extending the observation time from 6 hours to 12 hours and 24 hours. We found marked increase of tubule formation in $PGF_{2\alpha}$-treated HRMECs at 12 hours. As such, we replaced with the new **Figure 3F-3J**, **Figure 4K-L and Figure 6J-6K,** and modified the method and the results; please see **Page 8, Lines 164-167; Page 10, Line 198-200; Page 11, Lines 232-234; Page 26, Line 558.**

Reviewer #3

This is an interesting and well constructed study that merits publication after attention to a few minor points.

We would like to thank the Reviewer for favorable comments.

1. The study has clinical relevance because of the links with DR, but the only in vivo model used is the OIR postnatal mouse, which is not a model of DR and differs in several respects. In particular, pathology is driven by hypoxia and VEGF induction via HIF1a. Given that VEGF blockers work well in the OIR model, and also in many patients with DR, the authors need to provide some points of discussion to explain why they think targeting PGF2a therapeutically might work, as presumably the VEGF axis would continue to drive pathology.

We agree. Despite anti-VEGF therapy is widely used proliferative retinopathy, a large portion of patient still exhibit a poor or no response to VEGF-targeting drugs (*Drug Des Devel Ther* 2016; 10: 1857-1867). These clinical observations suggest the involvement of other signaling pathways in the promotion of retinal angiogenesis via the increased local

production of various pro-angiogenic factors or pro-inflammatory lipid mediators (*Front Immunol* 2020; 11: 581288). In this study, we observed $PGF_{2\alpha}$/PTGFR axis accelerates experimental proliferative retinopathy through the secretion of ELR^+ CXC chemokines via FOS/ CAMK2G/ P38 signal pathway, which is independent of VEGF/VEGFR signaling (**Appendix Figure S2A-D).** Therefore, we mentioned in the Introduction and Discussion; please see **Page 4, Lines 68-74; Page 17, Lines 355-360; Page 20, Lines 419-421)**

2. Line 130 - Cdh5 expression does not provide a measure of purity of the endothelial cells. A similar result would surely be observed in the presence of other cell types such as pericytes.

Thank you for the correction. As suggested, we performed magnetic-activated cell sorting (MACS) approach to purify endothelial cells (ECs) from retinas. We expanded the methods and results (**Figure 1G**), please see **Page 7, Lines 134-136 and Page 29, Paragraph 3.**

3. The legends to figures EV2, 3, 7 and 8 would benefit from a few lines of explanatory text.

We did. Please see new **Figure EV1, EV2**; and **Appendix Figure S3, 4 (**Due to EV figure limitation, we moved Figure EV7-8 to Appendix Figure S3, 4).

4. Some editorial input on the finished MS might be necessary to improve the written English in places.

Thanks for the suggestion. The manuscript was edited by professional agent (www. Editage.com).

2nd Nov 2022

Dear Prof. Yu,

Thank you for the submission of your revised manuscript to EMBO Molecular Medicine. I am pleased to inform you that we will be able to accept your manuscript pending the following final amendments:

1) In the main manuscript file, please do the following:
- In M&M, provide the antibody dilutions that were used for each antibody.
- In M&M, please include statement that in addition to the WMA Declaration of Helsinki the experiments using human samples also conformed to the principles set out in the Department of Health and Human Services Belmont Report.
- Data availability: In addition to the accession number please provide full URL for deposited datasets. Please be aware that all datasets should be made freely available upon acceptance, without restriction. Use the following format to report the accession number of your data:

The datasets produced in this study are available in the following databases:
[data type]: [full name of the resource] [accession number/identifier] ([doi or URL or identifiers.org/DATABASE:ACCESSION])

Please check "Author Guidelines" for more information.
https://www.embopress.org/page/journal/17574684/authorguide#availabilityofpublishedmaterial
2) Appendix: Please add "Table of Content" on the title page and place all figure legends under the respective figures.
3) Synopsis:
- Synopsis image: Please provide a separate high-resolution jpeg file 550 px-wide x (250-400)-px high.
- Please check your synopsis text and image before submission with your revised manuscript. Please be aware that in the proof stage minor corrections only are allowed (e.g., typos).
4) For more information: This space should be used to list relevant web links for further consultation by our readers. Could you identify some relevant ones and provide such information as well? Some examples are patient associations, relevant databases, OMIM/proteins/genes links, author's websites, etc...
5) As part of the EMBO Publications transparent editorial process initiative (see our Editorial at http://embomolmed.embopress.org/content/2/9/329), EMBO Molecular Medicine will publish online a Review Process File (RPF) to accompany accepted manuscripts. This file will be published in conjunction with your paper and will include the anonymous referee reports, your point-by-point response and all pertinent correspondence relating to the manuscript. Let us know whether you agree with the publication of the RPF and as here, if you want to remove or not any figures from it prior to publication. Please note that the Authors checklist will be published at the end of the RPF.
6) Please provide a point-by-point letter INCLUDING my comments as well as the reviewer's reports and your detailed responses (as Word file).

I look forward to reading a new revised version of your manuscript as soon as possible.

Yours sincerely,

Zeljko Durdevic

Zeljko Durdevic
Editor
EMBO Molecular Medicine

***** Reviewer's comments *****

Referee #1 (Comments on Novelty/Model System for Author):

The authors have addressed my concerns by their point-by point responses.

Referee #1 (Remarks for Author):

The authors have addressed my concerns by their point-by point responses.

Referee #3 (Comments on Novelty/Model System for Author):

This version of this manuscript has been extensively revised to take into account comments from the reviewers. The authors have responded carefully to these comments and undertaken significant additional studies. In some instances absence of reagents or models has prevented work from being undertaken, but then conclusions or interpretation have been moderated accordingly. At a personal level I still see places where improvements could be made to grammar and syntax, but readers will have no problem understanding the text.

The authors addressed the remaining editorial issues.

We are pleased to inform you that your manuscript is accepted for publication and is now being sent to our publisher to be included in the next available issue of EMBO Molecular Medicine.

EMBO Press Author Checklist

Corresponding Author Name: Niklas Mattsson-Carlgren	
Journal Submitted to: EMBO Molecular Medicine	
Manuscript Number: EMM-2022-16359	

USEFUL LINKS FOR COMPLETING THIS FORM
The EMBO Journal - Author Guidelines
EMBO Reports - Author Guidelines
Molecular Systems Biology - Author Guidelines
EMBO Molecular Medicine - Author Guidelines

Reporting Checklist for Life Science Articles (updated January 2022)
This checklist is adapted from Materials Design Analysis Reporting (MDAR) Checklist for Authors. MDAR establishes a minimum set of requirements in transparent reporting in the life sciences (see Statement of Task: 10.31222/osf.io/9sm4x). Please follow the journal's guidelines in preparing your manuscript.
Please note that a copy of this checklist will be published alongside your article.

Abridged guidelines for figures
1. Data
The data shown in figures should satisfy the following conditions:
- the data were obtained and processed according to the field's best practice and are presented to reflect the results of the experiments in an accurate and unbiased manner.
- ideally, figure panels should include only measurements that are directly comparable to each other and obtained with the same assay.
- plots include clearly labeled error bars for independent experiments and sample sizes. Unless justified, error bars should not be shown for technical replicates.
- if n<5, the individual data points from each experiment should be plotted. Any statistical test employed should be justified.
- Source Data should be included to report the data underlying figures according to the guidelines set out in the authorship guidelines on Data Presentation.

2. Captions
Each figure caption should contain the following information, for each panel where they are relevant:
- a specification of the experimental system investigated (eg cell line, species name).
- the assay(s) and method(s) used to carry out the reported observations and measurements.
- an explicit mention of the biological and chemical entity(ies) that are being measured.
- an explicit mention of the biological and chemical entity(ies) that are altered/varied/perturbed in a controlled manner.
- the exact sample size (n) for each experimental group/condition, given as a number, not a range;
- a description of the sample collection allowing the reader to understand whether the samples represent technical or biological replicates (including how many animals, litters, cultures, etc.).
- a statement of how many times the experiment shown was independently replicated in the laboratory.
- definitions of statistical methods and measures:
  - common tests, such as t-test (please specify whether paired vs. unpaired), simple χ2 tests, Wilcoxon and Mann-Whitney tests, can be unambiguously identified by name only, but more complex techniques should be described in the methods section;
  - are tests one-sided or two-sided?
  - are there adjustments for multiple comparisons?
  - exact statistical test results, e.g., P values = x but not P values < x;
  - definition of 'center values' as median or average;
  - definition of error bars as s.d. or s.e.m.

Please complete ALL of the questions below.
Select "Not Applicable" only when the requested information is not relevant for your study.

Materials

Newly Created Materials	Information included in the manuscript?	In which section is the information available? (Reagents and Tools Table, Materials and Methods, Figures, Data Availability Section)
New materials and reagents need to be available; do any restrictions apply?	Not Applicable	

Antibodies	Information included in the manuscript?	In which section is the information available? (Reagents and Tools Table, Materials and Methods, Figures, Data Availability Section)
For **antibodies** provide the following information:<br>- Commercial antibodies: RRID (if possible) or supplier name, catalogue number and or/clone number<br>- Non-commercial: RRID or citation	Yes	Supplier name is provided for OLINK assays

DNA and RNA sequences	Information included in the manuscript?	In which section is the information available? (Reagents and Tools Table, Materials and Methods, Figures, Data Availability Section)
**Short novel DNA or RNA including primers, probes**: provide the sequences.	Not Applicable	

Cell materials	Information included in the manuscript?	In which section is the information available? (Reagents and Tools Table, Materials and Methods, Figures, Data Availability Section)
**Cell lines:** Provide species information, strain. Provide accession number in repository **OR** supplier name, catalog number, clone number, and/**OR** RRID.	Not Applicable	
**Primary cultures:** Provide species, strain, sex of origin, genetic modification status.	Not Applicable	
Report if the cell lines were recently **authenticated** (e.g., by STR profiling) and tested for mycoplasma contamination.	Not Applicable	

Experimental animals	Information included in the manuscript?	In which section is the information available? (Reagents and Tools Table, Materials and Methods, Figures, Data Availability Section)
**Laboratory animals or Model organisms:** Provide species, strain, sex, age, genetic modification status. Provide accession number in repository **OR** supplier name, catalog number, clone number, **OR** RRID.	Not Applicable	
**Animal observed in or captured from the field:** Provide species, sex, and age where possible.	Not Applicable	
Please detail **housing and husbandry conditions**.	Not Applicable	

Plants and microbes	Information included in the manuscript?	In which section is the information available? (Reagents and Tools Table, Materials and Methods, Figures, Data Availability Section)
**Plants:** provide species and strain, ecotype and cultivar where relevant, unique accession number if available, and source (including location for collected wild specimens).	Not Applicable	
**Microbes:** provide species and strain, unique accession number if available, and source.	Not Applicable	

Human research participants	Information included in the manuscript?	In which section is the information available? (Reagents and Tools Table, Materials and Methods, Figures, Data Availability Section)
If collected and within the bounds of privacy constraints report on age, sex and gender or ethnicity for all study participants.	Yes	

Core facilities	Information included in the manuscript?	In which section is the information available? (Reagents and Tools Table, Materials and Methods, Figures, Data Availability Section)
If your work benefited from core facilities, was their service mentioned in the acknowledgments section?	Not Applicable	

Design

Study protocol	Information included in the manuscript?	In which section is the information available? (Reagents and Tools Table, Materials and Methods, Figures, Data Availability Section)
If study protocol has been **pre-registered, provide DOI in the manuscript**. For clinical trials, provide the trial registration number **OR** cite DOI.	Not Applicable	
Report the **clinical trial registration number** (at ClinicalTrials.gov or equivalent), where applicable.	Yes	Materials and Methods

Laboratory protocol	Information included in the manuscript?	In which section is the information available? (Reagents and Tools Table, Materials and Methods, Figures, Data Availability Section)
Provide DOI OR other citation details if **external detailed step-by-step protocols** are available.	Not Applicable	

Experimental study design and statistics	Information included in the manuscript?	In which section is the information available? (Reagents and Tools Table, Materials and Methods, Figures, Data Availability Section)
Include a statement about **sample size** estimate even if no statistical methods were used.	Yes	Materials and Methods
Were any steps taken to minimize the effects of subjective bias when allocating animals/samples to treatment (e.g. **randomization procedure**)? If yes, have they been described?	Not Applicable	
Include a statement about **blinding** even if no blinding was done.	Yes	Materials and Methods
Describe **inclusion/exclusion criteria** if samples or animals were excluded from the analysis. Were the criteria pre-established?<br><br>If sample or data points were omitted from analysis, report if this was due to attrition or intentional exclusion and provide justification.	Yes	Materials and Methods
For every figure, are **statistical tests** justified as appropriate? Do the data meet the assumptions of the tests (e.g., normal distribution)? Describe any methods used to assess it. Is there an estimate of variation within each group of data? Is the variance similar between the groups that are being statistically compared?	Yes	Figures

Sample definition and in-laboratory replication	Information included in the manuscript?	In which section is the information available? (Reagents and Tools Table, Materials and Methods, Figures, Data Availability Section)
In the figure legends: state number of times the experiment was **replicated** in laboratory.	Not Applicable	
In the figure legends: define whether data describe **technical or biological replicates**.	Not Applicable	

Ethics

Ethics	Information included in the manuscript?	In which section is the information available? (Reagents and Tools Table, Materials and Methods, Figures, Data Availability Section)
Studies involving **human participants**: State details of **authority granting ethics approval** (IRB or equivalent committee(s), provide reference number for approval.	Yes	Material and Methods
Studies involving **human participants**: Include a statement confirming that **informed consent** was obtained from all subjects and that the experiments conformed to the principles set out in the WMA Declaration of Helsinki and the Department of Health and Human Services Belmont Report.	Yes	Material and Methods
Studies involving **human participants:** For publication of **patient photos**, include a statement confirming that consent to publish was obtained.	Not Applicable	
Studies involving experimental **animals**: State details of **authority granting ethics approval** (IRB or equivalent committee(s), provide reference number for approval. Include a statement of compliance with ethical regulations.	Not Applicable	
Studies involving **specimen and field samples:** State if relevant **permits** obtained, provide details of authority approving study; if none were required, explain why.	Not Applicable	

Dual Use Research of Concern (DURC)	Information included in the manuscript?	In which section is the information available? (Reagents and Tools Table, Materials and Methods, Figures, Data Availability Section)
Could your study fall under dual use research restrictions? Please check biosecurity documents and list of **select agents and toxins** (CDC): https://www.selectagents.gov/sat/list.htm	Not Applicable	
If you used a select agent, is the security level of the lab appropriate and reported in the manuscript?	Not Applicable	
If a study is subject to dual use research of concern regulations, is the name of the **authority granting approval and reference number** for the regulatory approval provided in the manuscript?	Not Applicable	

Reporting

The MDAR framework recommends adoption of discipline-specific guidelines, established and endorsed through community initiatives. Journals have their own policy about requiring specific guidelines and recommendations to complement MDAR.

Adherence to community standards	Information included in the manuscript?	In which section is the information available? (Reagents and Tools Table, Materials and Methods, Figures, Data Availability Section)
State if relevant guidelines or checklists (e.g., **ICMJE, MIBBI, ARRIVE, PRISMA**) have been followed or provided.	Not Applicable	
For **tumor marker prognostic studies,** we recommend that you follow the **REMARK** reporting guidelines (see link list at top right). See author guidelines, under 'Reporting Guidelines'. Please confirm you have followed these guidelines.	Not Applicable	
For **phase II and III randomized controlled trials**, please refer to the **CONSORT** flow diagram (see link list at top right) and submit the CONSORT checklist (see link list at top right) with your submission. See author guidelines, under 'Reporting Guidelines'. Please confirm you have submitted this list.	Not Applicable	

Data Availability

Data availability	Information included in the manuscript?	In which section is the information available? (Reagents and Tools Table, Materials and Methods, Figures, Data Availability Section)
Have **primary datasets** been deposited according to the journal's guidelines (see 'Data Deposition' section) and the respective accession numbers provided in the Data Availability Section?	Yes	Materials and Methods
Were **human clinical and genomic datasets** deposited in a public access-controlled repository in accordance to ethical obligations to the patients and to the applicable consent agreement?	Not Applicable	
Are **computational models** that are central and integral to a study available without restrictions in a machine-readable form? Were the relevant accession numbers or links provided?	Not Applicable	
If publicly available data were reused, provide the respective **data citations in the reference list**.	Not Applicable	
